# Fish and maize: Bayesian mixing models of fourteenth- through seventeenth-century AD ancestral Wendat diets, Ontario, Canada

**DOI:** 10.1038/s41598-019-53076-7

**Published:** 2019-11-13

**Authors:** Robert S. Feranec, John P. Hart

**Affiliations:** 1 Research and Collections Division, New York State Museum, Albany, New York USA; 2 Research and Collections Division, New York State Museum, Albany, New York USA

**Keywords:** Palaeoecology, Stable isotope analysis

## Abstract

Freshwater and marine fish have been important components of human diets for millennia. The Great Lakes of North America, their tributaries and smaller regional freshwater bodies are important Native American fisheries. The ethnohistorical record, zooarchaeological remains, and isotopic values on human bone and tooth collagen indicate the importance of fish in fourteenth- through seventeenth-century ancestral Wendat diets in southern Ontario, which is bordered by three of the Great Lakes. Maize (*Zea mays* ssp. *mays*) was the primary grain of Native American agricultural systems in the centuries prior to and following sustained European presence. Here we report new Bayesian dietary mixing models using previously published δ^13^C and δ^15^N values on ancestral Wendat bone and tooth collagen and tooth enamel. The results confirm previous estimates from δ^13^C values that ancestral Wendat diets included high proportions of maize but indicate much higher proportions of fish than has previously been recognized. The results also suggest that terrestrial animals contributed less to ancestral Wendat diets than is typically interpreted based on zooarchaeological records.

## Introduction

**Figure 1:**
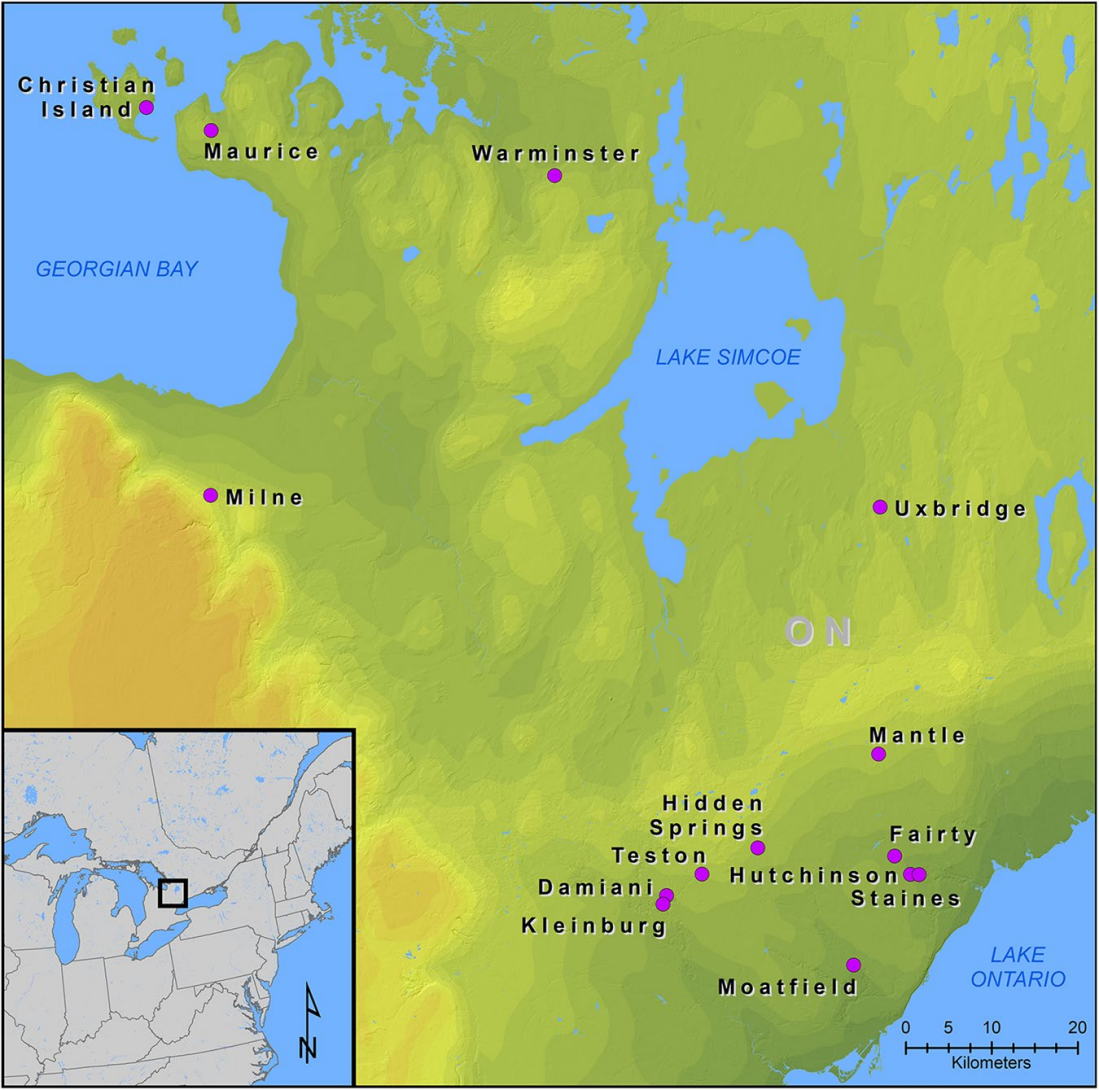
Locations of archaeological sites with samples used in the analyses. Figure 1 does not contain copyrighted material. The map was produced in ArcGIS v 10.6 at the New York State Museum, Albany, NY by compiling GIS data (shapefiles) obtained from publicly available data from Statistics Canada, the U.S. Census, and the United States Geological Survey.

Various lines of evidence indicate freshwater and marine fish have been important components of human diets for millennia^1–3^. Fish biomarkers extracted from pottery fabric and encrusted food residues have shown that fish were cooked in the early pottery in several areas of the world^4–7^. Fish skeletal elements and scales are routinely recovered from prehistoric archaeological sites worldwide when fine-scale recovery is implemented^8–11^. Isotopic analyses of human bone frequently indicate fish consumption^12,13^. Ethnohistoric and ethnographic accounts attest to the dietary importance of fish in the recent past^14,15^. These lines of evidence indicate that the Great Lakes of North America, their tributaries, and surrounding smaller freshwater bodies were important Native American fisheries^16^. Southern Ontario, Canada, borders Lake Ontario, Lake Erie, Lake Huron and its Georgian Bay, and the St. Lawrence River (Fig. 1), and fish were important dietary components of Native Americans occupying this area^17^.

Maize (*Zea mays* ssp. *mays*) was the primary grain of Native American agricultural systems in eastern North America in the centuries prior to and after European arrivals^18^. Phytoliths and starch recovered from directly AMS-dated charred cooking residues adhering to the interiors of pottery sherds indicate that maize was introduced into the lower Great Lakes region, presumably including southern Ontario, by ca. cal. 300 BC^19–21^. Macrobotanical remains in the form of charred maize kernels and cob fragments become evident in the southern Ontario archaeological record by ca. cal. AD 500^22^ and become ubiquitous by ca. cal. AD 900–1000^18^.

Southern Ontario was home to the historical Iroquoian Wendat (Huron) Confederacy, which began to form during the sixteenth century AD^23^. Over the course of the fourteenth through the beginning of the seventeenth centuries AD, ancestral Wendat communities moved northward from the north shore of Lake Ontario, and eventually converged on an area between the south shore of Georgian Bay to the north and Lake Simcoe to the south (Fig. 1)^23^. During these centuries, communities coalesced into large villages and towns and formed several nations, which in turn formed the Wendat Confederacy^24^. Multiple lines of evidence indicate that these communities relied substantially on maize for food, and that maize-based agriculture facilitated the coalescence of communities and the convergence of Wendat communities into a geographically restricted area^25^.

Maize is a plant that uses the C_4_ photosynthetic pathway with archaeological δ^13^C values in northeastern North America ranging from −15.1 to −7.4‰, while most native terrestrial plants and agricultural crops in the region utilize the C_3_ photosynthetic pathway with archaeological δ^13^C values in northeastern North America ranging from −28.6 to −23.3‰^26–30^. As a result, consumers of maize and their consumers have δ^13^C values that are higher than consumers of predominantly C_3_ photosynthetic pathway plants and their consumers. δ^13^C analyses of human bone collagen and apatite suggest that maize consumption began to increase gradually in southern Ontario by ca. cal. AD 500^27^. Using δ^13^C values on human bone and teeth, Pfeiffer *et al*.^28,29^ suggest that by the cal. fourteenth to fifteenth centuries AD, maize was a dominant source of calories for ancestral Wendat individuals, remained so in the sixteenth century AD, and increased in the seventeenth century AD. Using a linear mixing model with δ^13^C values they estimate that maize contributed >50% of ancestral Wendat diets during those centuries, and perhaps as much as 65%^28^. However, they suggest that elevated δ^13^C values may also reflect the consumption of freshwater fish because fish bone from ancestral Wendat archaeological sites have δ^13^C values that range mostly between −21‰ and −16‰^28^. Pfeiffer *et al*.^28^ report a mean dog collagen δ^13^C value of −11.1 ± 0.9‰ and suggest its consumption may have affected human values. Less attention has been paid to stable nitrogen isotopes (δ^15^N) in terms of ancestral Wendat diets, given that the primary concern of isotopic studies in southern Ontario has been to trace maize consumption histories and has thus focused on δ^13^C values.

Consumers of animal flesh have higher δ^15^N values than do plants and primary consumers of plants. Omnivores with higher proportions of terrestrial plants in their diets will have lower δ^15^N than omnivores with higher proportions of animal flesh in their diets. Of note is that despite the dominance of maize in the diet, δ^15^N values in Pfeiffer *et al*.’s^28^ ancestral Wendat samples are highly elevated. Because maize, like other terrestrial plants, is assumed to have δ^15^N values < 3.0‰, Pfeiffer *et al*.^28^ suggest diets that included large amounts of terrestrial resources should produce δ^15^N values ≤ 10‰. Given that δ^15^N values of ancestral Wendat individuals are generally >10‰, Pfeiffer *et al*., like others^27,30^, suggest that ancestral Wendat diets included high-trophic-level freshwater fish.

There is ample evidence from the archaeological and ethnohistoric records that freshwater fish was an important contributor to ancestral and historical Wendat diets^15,17,31^. Based on the ethnohistorical record, Heidenrich^15^ estimated that fish accounted for 9% of seventeenth century AD daily Wendat diets. Fish bone generally comprises large proportions of faunal assemblages on seventeenth-century and earlier ancestral Wendat sites on which fine-scale recovery was used, and this evidence suggests varied fishing strategies based on time of year and settlement location^32,33^. While ancestral Wendat δ^15^N values suggest fish were important components of diets, no quantitative assessments have been made based on those values^28,31,34^. On the basis of δ^15^N values Pfeiffer *et al*.^28^ suggest that proportions of fish in diets were highest during the fourteenth and seventeenth centuries AD, when ancestral Wendat communities were closest to the north shore of Lake Ontario and the south shore of Georgian Bay, respectively. They suggest dietary fish proportions decreased during the fifteenth and sixteenth centuries AD when communities were located at greater distances from these two fisheries. Hawkins *et al*.^17^ suggest their analysis of a large dataset of fish remains supports Pfeiffer *et al*.’s conclusion, and the contribution of fish to diets was particularly low during the sixteenth century AD before recovering to substantial contributions during the seventeenth century AD.

Based on differences in δ^13^C and δ^15^N values in bone collagen and dentine collagen samples, Pfeiffer *et al*.^28^ suggest that there were differences between adult and child diets, respectively. Specifically, they suggest maize comprised larger proportions of child than adult diets, while animal protein comprised smaller proportions of child than adult diets.

Potentially complicating interpretations of δ^15^N values is the role of white-tailed deer (*Odocoileus virginianus*) in subsistence^34^. While deer is sometimes considered to have been an important component of ancestral Wendat diets^28^, deer bone frequently does not comprise high percentages of faunal remain assemblages^35^. Seventeenth-century AD ethnohistoric records indicate that deer was not a major component of Wendat diets—deer were scarce in the area occupied by the Wendat at that time^15^. To the contrary, good deer habitat occurs to the south where ancestral Wendat communities lived prior to consolidation south of Georgian Bay in the seventeenth century AD^35^.

Here we apply a Bayesian dietary mixing model, MixSIAR^36^, to calculate the proportions of food sources in ancestral Wendat diets. MixSIAR allows multiple isotopic tracers (e.g., δ^13^C and δ^15^N) and uses a Markov Chain Monte Carlo simulation to model the probability of proportions of food sources in a consumer’s diet based on the isotopic values of the food sources (e.g., maize, fish) and the consumer (human). As opposed to identifying point estimates of the proportions of certain foods in diets (e.g., maize) from the analysis of single isotopic tracers, Bayesian mixing models, including MixSIAR used here, incorporate the uncertainty in the isotopic values of the food sources, the consumer, and the trophic enrichment factor (TEF) between the food sources and the consumer in the model to ultimately produce a probability of proportions of food sources in a consumer’s diet. An assumption of these models is that the isotopic tracer values of the food sources are representative of the diets of the sampled humans.

We use δ^13^C and δ^15^N values in human dentine and bone collagen and tooth enamel from Pfeiffer *et al*.’s^28,29,34^ ancestral Wendat research, recently obtained isotope values for maize from ancestral Wendat sites, and a large database of terrestrial animal (n = 404) and freshwater fish (n = 170) bone collagen isotope data from southern Ontario archaeological sites, which we compiled from several sources^28,37–40^, to model fourteenth through seventeenth century AD ancestral Wendat diets. We specifically test four hypotheses generated by the results of Pfeiffer *et al*.’s^28^ interpretations of their stable isotope analyses and Hawkins *et al*.’s^17^ interpretation of ancestral Wendat fish consumption based on faunal remains:

H_1_: The proportions of maize in diets was consistent from the fourteenth through sixteenth centuries AD and increased in the seventeenth century AD.

H_2_: Diets incorporated proportionately more fish during the fourteenth and seventeenth centuries AD than during the intervening centuries, with fish being particularly minor dietary components in the sixteenth century AD diets.

H_3_: Fish exploitation was largely concentrated on high trophic-level species.

H_4_: Children, as reflected in dentine collagen and tooth enamel isotope values, had higher proportions of maize in their diets than did adults as reflected in bone collagen isotope values.

This article provides results of the first application of multi-tracer Bayesian dietary modeling on a large series of Native American isotopic values from eastern North America. The combined modeling of δ^13^C and δ^15^N values results in the first quantitative estimates of fish proportions in ancestral Wendat diets based on archaeological isotopic data.

## Results

**Figure 2:**
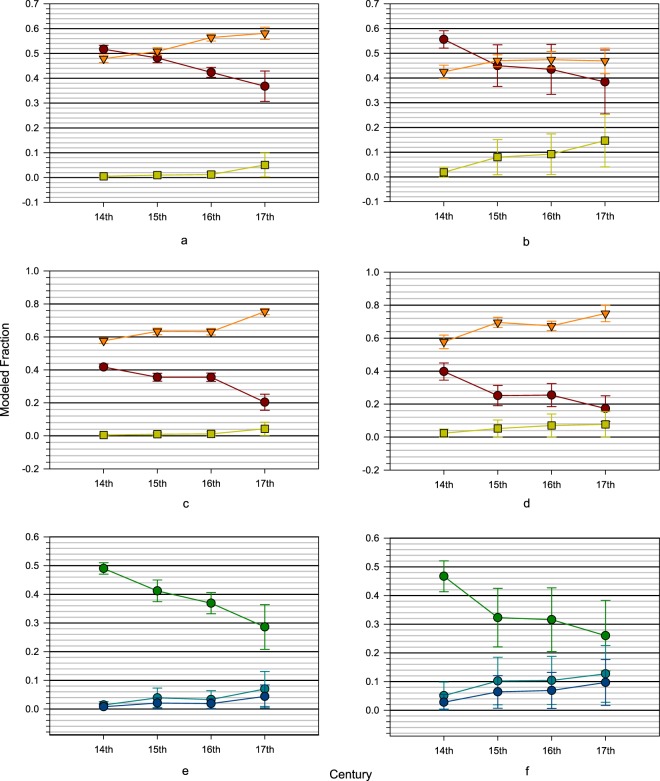
Mean values of three-source models by century for (**a**) dentine collagen, (**b**) bone collagen, (**c**) enamel-dentine collagen, (**d**) enamel-bone collagen. Red circles = fish, orange triangles = maize, green squares = terrestrial animals. Mean values of three fish categories in the five-source models by century for (**e**) dentine collagen and (**f**) bone collagen. Green circles = high δ^15^N fish, light blue circles = medium δ^15^N fish, and dark blue circles = low δ^15^N fish. Error bars are 1 standard deviation.

All model output and code are presented in the Supplementary Information File. Results of the three-source (i.e., maize, fish, and terrestrial species) MixSIAR models by century are summarized in Fig. 2 and Table 1. Isotopes are differentially incorporated into tissues such that the isotopic values in collagen (bone or dentine) derive mainly from ingested protein, while values in tooth enamel derive from the whole diet^41–44^. This routing of isotopes may influence interpretations of diet in populations if they are based solely on one tissue type. Additionally, these tissues may reflect different periods in life depending on when the tissue forms. Tooth enamel and dentine collagen form during tooth development and do not readily remodel, so the δ^13^C and δ^15^N values are reflective of intake at younger ages. Bone collagen, however, does remodel and, in general, the isotopic values reflect intake of an older individual. By comparing values from different tissues, such as tooth enamel and bone collagen, a more comprehensive understanding of dietary source proportions is derivable.

**Table 1:** Three-source model estimates by tissue and resource group.

Tissues (δ^13^C-δ^15^N)	Resource Group	Century (AD)	n	Mean	95% Range
Dentine Collagen-Dentine Collagen	Fish	Fourteenth	78	0.517 ± 0.016	0.484–0.548
		Fifteenth	34	0.482 ± 0.019	0.442–0.516
		Sixteenth	34	0.424 ± 0.020	0.378–0.458
		Seventeenth	17	0.368 ± 0.061	0.222–0.455
	Maize	Fourteenth	78	0.479 ± 0.015	0.448–0.507
		Fifteenth	34	0.508 ± 0.015	0.479–0.536
		Sixteenth	34	0.564 ± 0.014	0.535–0.592
		Seventeenth	17	0.581 ± 0.025	0.530–0.626
	Other terrestrial	Fourteenth	78	0.005 ± 0.005	0.000–0.018
		Fifteenth	34	0.010 ± 0.010	0.000–0.037
		Sixteenth	34	0.012 ± 0.012	0.000–0.044
		Seventeenth	17	0.051 ± 0.048	0.001–0.175
Bone Collagen-Bone Collagen	Fish	Fourteenth	21	0.556 ± 0.035	0.486–0.619
		Fifteenth	15	0.450 ± 0.084	0.243–0.558
		Sixteenth	10	0.435 ± 0.101	0.403–0.572
		Seventeenth	6	0.384 ± 0.129	0.097–0.593
	Maize	Fourteenth	21	0.425 ± 0.027	0.367–0.475
		Fifteenth	15	0.470 ± 0.024	0.423–0.517
		Sixteenth	10	0.474 ± 0.033	0.403–0.533
		Seventeenth	6	0.469 ± 0.052	0.350–0.555
	Other terrestrial	Fourteenth	21	0.019 ± 0.020	0.000–0.069
		Fifteenth	15	0.080 ± 0.071	0.000–0.037
		Sixteenth	10	0.092 ± 0.082	0.002–0.310
		Seventeenth	6	0.147 ± 0.106	0.006–0.395
Tooth Enamel-Dentine Collagen	Fish	Fourteenth	78	0.418 ± 0.018	0.383–0.455
		Fifteenth	34	0.356 ± 0.024	0.307–0.402
		Sixteenth	34	0.356 ± 0.025	0.302–0.404
		Seventeenth	17	0.204 ± 0.049	0.077–0.270
	Maize	Fourteenth	78	0.577 ± 0.017	0.542–0.609
		Fifteenth	34	0.634 ± 0.020	0.593–0.671
		Sixteenth	34	0.632 ± 0.020	0.588–0.668
		Seventeenth	17	0.754 ± 0.019	0.717–0.791
	Other terrestrial	Fourteenth	78	0.005 ± 0.005	0.000–0.019
		Fifteenth	34	0.010 ± 0.011	0.000–0.040
		Sixteenth	34	0.012 ± 0.013	0.000–0.046
		Seventeenth	17	0.043 ± 0.040	0.001–0.150
Tooth Enamel-Bone Collagen	Fish	Fourteenth	21	0.398 ± 0.052	0.293–0.506
		Fifteenth	15	0.252 ± 0.062	0.095–0.348
		Sixteenth	10	0.255 ± 0.070	0.081–0.358
		Seventeenth	6	0.173 ± 0.078	0.020–0.323
	Maize	Fourteenth	21	0.578 ± 0.041	0.479–0.642
		Fifteenth	15	0.696 ± 0.031	0.631–0.753
		Sixteenth	10	0.675 ± 0.028	0.615–0.726
		Seventeenth	6	0.750 ± 0.050	0.632–0.827
	Other terrestrial	Fourteenth	21	0.024 ± 0.025	0.001–0.096
		Fifteenth	15	0.052 ± 0.048	0.001–0.180
		Sixteenth	10	0.070 ± 0.058	0.003–0.219
		Seventeenth	6	0.077 ± 0.058	0.003–0.211

Mean values for the models evince similar trends, that is, the proportion of maize consumed increases while the proportion of fish consumed decreases over time. The dentine collagen-based model exhibits an increase in maize proportions through time, a steady decrease in fish proportions, and a slight increase in terrestrial animal proportions in the seventeenth century AD. The bone collagen model exhibits an increase in maize proportions from the fourteenth to fifteenth century AD followed by a plateau, an overall decrease in fish proportions, but with a plateau in the fifteenth and sixteenth centuries AD, and an increase in terrestrial animal proportions but with a plateau in the fifteenth and sixteenth centuries AD. Overall the dentine collagen model mean values exhibit higher maize and lower terrestrial animal proportions than the means of the bone collagen model. As would be expected these results are substantially the same when the results of individual site models are placed in relative chronological order (Supplementary File S1.4.3).

In both tooth enamel models (Fig. 2c,d), maize proportions increase with a plateau during the fifteenth and sixteenth centuries, while fish proportions decrease with a plateau in the fifteenth and sixteenth centuries. The tooth enamel carbonate models calculate higher mean proportions of maize and lower proportions of fish in Wendat diets, over the four centuries when comparing the collagen (bone and dentine) to tooth enamel model results (Fig. 2). This implies that the collagen models slightly under represent the proportion of maize in the diet. These results indicate we can reject H_1_—the dentine model exhibits a consistent increase in maize, the bone collagen model does not show an increase in the seventeenth century AD, and the tooth enamel models show an overall increase in maize with a plateau between the fifteenth and sixteenth centuries AD.

The means of all models evince decreases in fish proportions through time. None of the models indicate an increase in fish proportions in the seventeenth century AD contrary to Pfeiffer *et al*.’s^28^ interpretation of δ^15^N values and Hawkins *et al*.’s^17^ interpretation of fish remains. As a result, H_2_ can be rejected. Results for fish proportion means from the five-source models are summarized in Fig. 2 and Table 2. We cannot reject H_3_ given that high δ^15^N fish have the highest dietary proportion estimates for fish throughout the sequence in both models.

**Table 2:** Five–source model estimates by resource by century.

Resource	Century (AD)	Dentine Collagen			Bone Collagen		
		n	Mean	95% Range	n	Mean	95% Range
High δ^15^N Fish	Fourteenth	78	0.490 ± 0.020	0.448–0.524	21	0.467 ± 0.054	0.344–0.551
	Fifteenth	34	0.412 ± 0.038	0.322–0.474	15	0.323 ± 0.102	0.085–0.489
	Sixteenth	34	0.369 ± 0.037	0.278–0.427	10	0.316 ± 0.111	0.069–0.502
	Seventeenth	17	0.286 ± 0.078	0.110–0.412	6	0.260 ± 0.123	0.032–0.490
Medium δ^15^N Fish	Fourteenth	78	0.014 ± 0.012	0.001–0.047	21	0.051 ± 0.047	0.002–0.176
	Fifteenth	34	0.039 ± 0.034	0.001–0.126	15	0.102 ± 0.083	0.003–0.306
	Sixteenth	34	0.033 ± 0.031	0.001–0.117	10	0.104 ± 0.084	0.003–0.312
	Seventeenth	17	0.070 ± 0.061	0.001–0.226	6	0.127 ± 0.099	0.005–0.364
Low δ^15^N Fish	Fourteenth	78	0.008 ± 0.008	0.000–0.028	21	0.028 ± 0.027	0.001–0.097
	Fifteenth	34	0.021 ± 0.019	0.000–0.072	15	0.064 ± 0.057	0.002–0.206
	Sixteenth	34	0.019 ± 0.018	0.001–0.067	10	0.069 ± 0.063	0.002–0.232
	Seventeenth	17	0.044 ± 0.040	0.001–0.146	6	0.097 ± 0.080	0.004–0.300
Maize	Fourteenth	78	0.483 ± 0.014	0.454–0.510	21	0.433 ± 0.024	0.385–0.477
	Fifteenth	34	0.515 ± 0.013	0.488–0.541	15	0.463 ± 0.021	0.418–0.505
	Sixteenth	34	0.567 ± 0.014	0.539–0.593	10	0.460 ± 0.035	0.381–0.516
	Seventeenth	17	0.573 ± 0.025	0.520–0.617	6	0.439 ± 0.063	0.280–0.532
Terrestrial animals	Fourteenth	78	0.005 ± 0.005	0.000–0.017	21	0.020 ± 0.018	0.001–0.068
	Fifteenth	34	0.013 ± 0.012	0.000–0.046	15	0.049 ± 0.044	0.002–0.169
	Sixteenth	38	0.012 ± 0.011	0.000–0.042	10	0.051 ± 0.048	0.001–0.172
	Seventeenth	17	0.028 ± 0.028	0.001–0.105	6	0.076 ± 0.067	0.002–0.250

Based on the preceding, it is evident that child and adult diets were different in terms of maize and terrestrial animal proportions, but not for fish. As a result, H_4_ cannot be rejected–child and adult diets did differ in the respective samples.

## Discussion

Previous δ^13^C analyses of human bone and teeth and the ethnohistorical record suggest maize was a major component of ancestral Wendat diets, perhaps comprising upwards of 65% of the diet. Bayesian analysis shows that maize dietary proportions increased in importance from the fourteenth to seventeenth centuries AD, at least in adults. As evidenced by the dentine collagen model, maize appears to also have been an increasingly important component of child diets through the sequence. The bone- and dentine-collagen models suggest that about 50% of the protein derived from maize, and the tooth enamel models imply mean maize proportions over 60% throughout most of the analyzed sequence supporting the assessment that maize at times was the major component of ancestral Wendat diets. One estimate of fish consumption of 9% is based on seventeenth-century ethnohistorical records^15^. The Bayesian three-source models indicate fish proportions decreased through time, but the mean calculations for proportion both the dentine collagen and enamel models of fish in the diet was always above 9% across the fourteenth through seventeenth centuries. In adult diets decreased dietary fish proportion was replaced by terrestrial animals. The three-source model using enamel δ^13^C follow the same trends for child diets with increasing maize and decreasing fish proportions. The decreased proportions of fish in child diets was replaced primarily by increased maize, combined with terrestrial animals in the seventeenth century AD in both the dentine collagen and enamel models. Previous δ^15^N analyses suggest high-trophic-level fish, that typically have high δ^15^N values, were an important component of ancestral Wendat diets. The five-source collagen models show that the high δ^15^N fish group, which includes salmonids, is the dominant fish source in all bone and dentine collagen model iterations.

These results suggest that maize and fish were regular components of ancestral Wendat diets throughout the sequence. Both constituted large proportions of the diet contributing to the formation of dentine and bone collagen as well as tooth enamel. Other foods, such as terrestrial animals contributed substantially less. Deer flesh apparently was not the primary source of animal protein, rather, fish was the primary source, probably due to an easier access to fish compared to deer. Large fish like salmons can be passively trapped from spring to fall with wooden hoop nets while deer were captured using fence and pens and needed a significant number of experienced people to build the structures and drive the deer into them^15^. The increased proportion of terrestrial animals in the seventeenth-century AD bone collagen model may reflect deer consumption, which would be at odds with poor deer habitat in the region south of Georgian Bay. In earlier centuries, with villages located in favorable deer habitat, the mean dietary proportion of terrestrial animals contributing to collagen formation was <10%, while during the fourteenth century, i.e. when villages were more concentrated close to Lake Ontario and closer larger fish sources, the mean value is ~2%. This latter value may also be explained if deer harvesting was seasonal as recorded in the ethnohistoric record and deer flesh was limited to immediate consumption or associated feasts. Similarly, the ethnohistoric record indicates dog flesh, which has high δ^13^C, was generally eaten ceremonially and at feasts rather than frequently^15^. This occasional consumption would not influence human isotopic values.

As with previous isotopic analyses of human tissue in southern Ontario^27–30^, our analysis is limited by small sample sizes. However, the present analysis contributes to our understandings of ancestral Wendat diets by allowing us to assign quantitative estimates of fish and terrestrial animal dietary proportions for the individuals represented in the available samples. As in other areas of the world, while changes occurred over the centuries considered here, freshwater fish was a critical component of these individuals’ diets, while terrestrial protein was of substantially less importance. These results should inform future inferences about ancestral Wendat diets based on zooarchaeological analyses.

## Methods

For the Bayesian models, we used the MixSIAR GUI v. 3.1 in R v. 3.5.2^45^. Stable carbon (δ^13^C) and nitrogen (δ^15^N) isotope values from southern Ontario archaeological sources were used as the isotopic tracers. Data for animal bone recovered primarily from southern Ontario archaeological sites dating from AD 900–1700 (source) (Supplementary Information File S.2) were compiled from published sources^28,37–40^. Nine of the assayed fish bones are from the St. Lawrence Valley in Quebec and six are nineteenth-century sites in southern Ontario^38^. Data for humans (consumers) were obtained from Pfeiffer *et al*.^28^ (Supplementary Information File S3). To utilize the human tooth enamel data in the Bayesian models that employ collagen stable isotope data for sources and trophic enrichment factors, we converted the tooth enamel δ^13^C values using a Δ^13^C_enamel-collagen_ value of −5.7‰ (Supplementary Information File S1.2). (Zhu and Sealy, 2018). For the δ^15^N values, we used the dentine and bone collagen δ^15^N of the same teeth. Maize δ^13^C and δ^15^N values were obtained from ancestral Wendat site samples, reported here for the first time (Table S.4.1) and are the first published δ^15^N values on maize in the region. Three-source and five-source models were run and presented on data from each site separately, then run and presented combining data from sites into their respective centuries as in Pfeiffer *et al*.^28^. Since Hawkins *et al*. (2019) found no zooarchaeological evidence of Atlantic salmon (*Salmo salar*) in 17^th^ c. Wendat localities, we ran additional models of the 17^th^ century data in MixSIAR removing samples of this species from the fish source (3 source model) and the high δ^15^N fish source (5 source model). Results of these additional models showed extremely limited changes in the modelled proportions of sources consumed (Supplementary Information File S1.4.1.1, S1.4.6.1) The three-source model groups included maize (n = 14), all fish (n = 170), and all terrestrial prey (n = 404). The five-source-model groups had the all fish group split into three different groups based on statistically significant differences in δ^15^N values among fish taxa. The statistically significant differences in δ^15^N values also capture known ecological differences in the analyzed fish species with the high δ^15^N fish encompassing offshore fish species, while the medium and low δ^15^N fish groups contain nearshore fish species. Therefore, the five-source model groups included, maize (n = 14), all terrestrial prey (n = 404), high δ^15^N fish (n = 104), medium δ^15^N fish (n = 43), and low δ^15^N fish (n = 23) (see Supplementary Information File S1.1). Terrestrial prey included the following species: bear (*Ursus americanus*, n = 50), beaver (*Castor canadensis*, n = 3), deer (*Odocoileus virginianus*, n = 191), fox (*Vulpes vulpes*, n = 6), groundhog (*Marmota monax*, n = 18), muskrat (*Ondatra zibithecus*, n = 3), porcupine (*Erethizon dorsatum*, n = 3), rabbit/hare (*Leporidae*, n = 8), raccoon (*Procyon lotor*, n = 47), squirrel (*Sciurus carolinensis*, n = 14), and turkey (*Meleagris gallopavo*, n = 61). While obviously not capturing all potential variation in isotope values for the various taxa and isotopic groupings, the source value dataset is large enough to avoid modeling issues arising from small source sample sizes^46,47^ in the isotopic groups used here. Additionally, we found no statistically significant differences between salmon data from Ontario Ancestral Wendat sites and the remaining salmon samples implying baseline values from different watersheds were similar for the fish sources in the models (Supplementary Information File S.1.1). Because the maize δ^15^N values were higher than expected we carried out a series of experiments to determine if charring affects maize isotopic values and adjusted the maize δ^15^N values used in the Bayesian modeling accordingly (Supplementary Information File S.4). Sources were input as means and standard deviations. Collagen to collagen source (food) to consumer (human) trophic enrichment factors (TEF) used in the collagen models were +1.1‰ ± 0.2‰ for δ^13^C and +3.8‰ ± 1.1‰ δ^15^N^48,49^. The maize (source) to consumer (human) TEF for collagen was +5.0‰ ± 0.1‰ for δ^13^C, and +3.0‰ ± 0.1‰ for δ^15^N^43^. The Bayesian mixing models met the criteria of the Gelman-Rubin and Geweke diagnostics. Results are reported as means and standard deviations as well as percent credible interval range (posterior probabilities) from 0.025 to 0.975.

## Supplementary information

Fish and maize: Bayesian mixing models of fourteenth- through seventeenth-century AD ancestral Wendat diets, Ontario, Canada

## Data Availability

All data generated or analyzed during this study are included in this published article and its Supplementary Information File.
