# Supplementary material for: Fish and maize: Bayesian mixing models of fourteenth- through seventeenth-century AD ancestral Wendat diets, Ontario, Canada
Source: Sci Rep. 2019 Nov 13;9:16658. doi: 10.1038/s41598-019-53076-7 (PMC6853935; doi:10.1038/s41598-019-53076-7)
Supplement: Supplementary file 1 — Fish and maize: Bayesian mixing models of fourteenth- through seventeenth-century AD ancestral Wendat diets, Ontario, Canada [file 41598_2019_53076_MOESM1_ESM.docx]

**Fish and maize: Bayesian mixing models of fourteenth- through seventeenth-century AD ancestral Wendat diets, Ontario, Canada**

Robert S. Feranec and John P. Hart

**Supplementary Information**

**S1. MixSIAR: Inputs, Model Text, Summary Statistics, and Posterior Probability Plots**

Within this study we utilized the MixSIAR GUI v. 3.1 in R v. 3.5.2. Input data are provided below as well as in Supplementary Information S2. and S3. Model text developed by the MixSIAR GUI is provided below as well as the summary statistics and posterior probability plots for each model that was run.

**S1.1. MixSIAR Source Groups:**

Initial models utilized three source groups (maize, fish, and terrestrial species). These groups were chosen based on the hypotheses. These groups are also distinct isotopically in both δ^13^C and δ^15^N values. Analysis of variance (ANOVA) showed significant differences among these three groups in δ^13^C (F(2, 586) = 307.2, p<0.001), and in δ^15^N (F(2, 586) = 302.0, p<0.0001). Post‑hoc Tukey HSD tests showed that all three groups were statistically different in δ^13^C at p<0.05, while only the fish group was statistically significantly different from the maize and terrestrial groups in δ^15^N.

To determine whether a certain type of fish was contributing more to the Wendat populations from the 14^th^ to the 17^th^ centuries AD, we ran additional MixSIAR models ultimately splitting the fish into separate groups based on their δ^15^N values, resulting in a five source MixSIAR model. Fish data were initially split based on the ecological groupings (e.g., catadromous, non‑piscivorous) found in Pfeiffer et al. (2016), and the δ^13^C and δ^15^N values compared among those groups. Following, an analysis of variance (ANOVA) showed no significant differences among these ecological groupings in δ^13^C (F(2, 168) = 0.02, p=0.978), while it did show statistically significant differences among the ecological groups in δ^15^N (F(2, 168) = 102.2, p<0.0001). Post‑hoc Tukey HSD tests showed three statistically different groups based on the δ^15^N values, those ecological groups with high δ^15^N values, those with low δ^15^N values, and those with medium δ^15^N values. Comparison of Atlantic salmon (*Salmo salar*) isotope values from Ontario Ancestral Wendat sites to the remaining values for all remaining salmon samples in the dataset showed no statistically significant differences implying that baseline values for fish sources in the Bayesian models were similar among the various watersheds (Table S1.1).

Table S1.1. Two sample tests for Ontario Ancestral Wendat sites vs. all other salmon isotope values.

| Isotope | Difference in means | Bootstrap range | t | p | F | p | U | z | p |
| --- | --- | --- | --- | --- | --- | --- | --- | --- | --- |
| δ^15^N | 0.3885 | -0.0746-0.9185 | 1.2954 | 0.1945 | 1.7063 | 0.2892 | 507.3 | 1.3377 | 0.1880 |
| δ^13^C | 0.6005 | -0.8041-2.0425 | 0.8164 | 0.4229 | 2.4743 | 0.0174 | 626.5 | 0.0989 | 0.9185 |

All p-values are Monte Caro permutation except δ^13^C t-test, which is for unequal variance. Tests performed in PAST 3.25 (Hammer et al 2001).

**S1.2. MixSIAR Trophic Enrichment Factor (TEF):**

The collagen to collagen source (food) to consumer (human) trophic enrichment factors (TEF) used in the models was +1.1‰ ± 0.2‰ for δ^13^C, and +3.8‰ ± 1.1‰ for δ^15^N as used in Bocherens et al. (2015) and Ledogar et al. (2018). The maize (source) to consumer (human) TEF was +5.0‰ ± 0.1‰ for δ^13^C, and +3.0‰ ± 0.1‰ for δ^15^N. We decided the TEF for δ^15^N from Bocherens et al. (2015) and utilized by Ledogar et al. (2018) in a study on a northeast USA human, is the best suited for this study. The Bocherens et al. (2015) TEF is based on an average from multiple studies that examined collagen (source) to collagen (consumer) TEFs in modern and ancient mammals. This TEF is lower than that proposed by Collins et al. (2012) that suggested a TEF closer to +6.0‰, but the Collins et al. (2012) value is based on extrapolation from a TEF from diet to red blood cells, a tissue that has a much higher isotopic turnover rate than collagen. Even so, because MixSIAR is able to take isotopic uncertainty into account, the +3.8‰ ± 1.1‰ TEF for δ^15^N used in this study should account for higher values in the model such as those posed by Collins et al. (2012).

**S1.3. Model text (developed by the MixSIAR GUI):**

# source$data_type: means

# source$by_factor: NA

# random effects: 0

# fixed effects: 0

# nested factors:

# factors:

# continuous effects: 0

# error structure: Residual * Process

# source$conc_dep: FALSE

model{

for(src in 1:n.sources){

for(iso in 1:n.iso){

src_mu[src,iso] ~ dnorm(MU_array[src,iso], n_array[src]/SIG2_array[src,iso]); # Eqn 3.8 but with precision instead of variance

tmp.X[src,iso] ~ dchisqr(n_array[src]);

src_tau[src,iso] <- tmp.X[src,iso]/(SIG2_array[src,iso]*(n_array[src] - 1)); # Eqn 3.9, following the simulation on p.580

}

}

# draw p.global (global proportion means) from an uninformative Dirichlet,

# then ilr.global is the ILR-transform of p.global

p.global[1:n.sources] ~ ddirch(alpha[1:n.sources]);

for(src in 1:(n.sources-1)){

gmean[src] <- prod(p.global[1:src])^(1/src);

ilr.global[src] <- sqrt(src/(src+1))*log(gmean[src]/p.global[src+1]); # page 296, Egozcue 2003

}

# DON'T generate individual deviates from the global/region/pack mean (but keep same model structure)

for(i in 1:N) {

for(src in 1:(n.sources-1)) {

ilr.ind[i,src] <- 0;

ilr.tot[i,src] <- ilr.global[src] + ilr.ind[i,src]; # add all effects together for each individual (in ilr-space)

}

}

# Inverse ILR math (equation 24, page 294, Egozcue 2003)

for(i in 1:N){

for(j in 1:(n.sources-1)){

cross[i,,j] <- (e[,j]^ilr.tot[i,j])/sum(e[,j]^ilr.tot[i,j]);

}

for(src in 1:n.sources){

tmp.p[i,src] <- prod(cross[i,src,]);

}

for(src in 1:n.sources){

p.ind[i,src] <- tmp.p[i,src]/sum(tmp.p[i,]);

}

}

for(src in 1:n.sources) {

for(i in 1:N){

# these are weights for variances

p2[i,src] <- p.ind[i,src]*p.ind[i,src];

}

}

# for each isotope and population, calculate the predicted mixtures

for(iso in 1:n.iso) {

for(i in 1:N) {

mix.mu[iso,i] <- inprod(src_mu[,iso],p.ind[i,]) + inprod(frac_mu[,iso],p.ind[i,]);

}

}

# Multiplicative residual error

for(iso in 1:n.iso){

resid.prop[iso] ~ dunif(0,20);

}

# Calculate process variance for each isotope and population

for(iso in 1:n.iso) {

for(i in 1:N) {

process.var[iso,i] <- inprod(1/src_tau[,iso],p2[i,]) + inprod(frac_sig2[,iso],p2[i,]);

}

}

# Construct Sigma, the mixture precision matrix

for(ind in 1:N){

for(i in 1:n.iso){

for(j in 1:n.iso){

Sigma.ind[ind,i,j] <- equals(i,j)/(process.var[i,ind]*resid.prop[i]);

}

}

}

# Likelihood

for(i in 1:N) {

X_iso[i,] ~ dmnorm(mix.mu[,i], Sigma.ind[i,,]);

loglik[i] <- logdensity.mnorm(X_iso[i,], mix.mu[,i], Sigma.ind[i,,]);

}

} # end model

**S1.4. Summary statistics and posterior probability plots**

**S1.4.1. 3 Source Models (Maize, Fish, Terrestrial Prey) from Dentine Collagen by Century**

**14^th^ Century:**

**Mean SD 2.5% 5% 25% 50% 75% 95% 97.5%**

**Maize (corn)** 0.479 0.015 0.448 0.454 0.469 0.479 0.489 0.503 0.507

**Fish** 0.517 0.016 0.484 0.490 0.506 0.517 0.527 0.542 0.548

**Terrestrial** 0.005 0.005 0.000 0.000 0.001 0.003 0.007 0.015 0.018


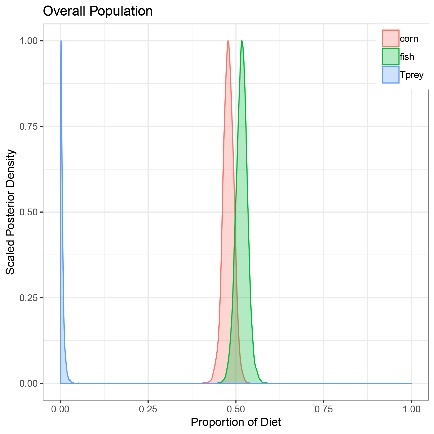


**15^th^ Century:**

**Mean SD 2.5% 5% 25% 50% 75% 95% 97.5%**

**Maize (corn)** 0.508 0.015 0.479 0.484 0.498 0.508 0.518 0.532 0.536

**Fish** 0.482 0.019 0.442 0.449 0.471 0.483 0.494 0.511 0.516

**Terrestrial** 0.010 0.010 0.000 0.000 0.003 0.007 0.015 0.032 0.037


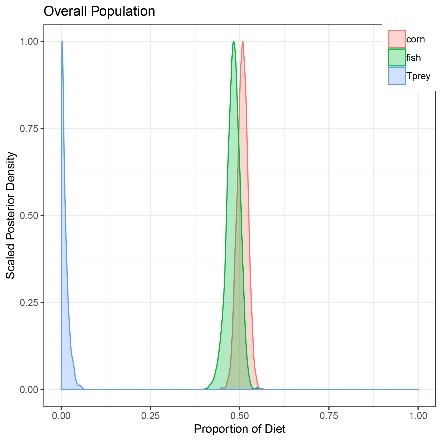


**16^th^ Century:**

**Mean SD 2.5% 5% 25% 50% 75% 95% 97.5%**

**Maize (corn)** 0.564 0.014 0.535 0.540 0.555 0.564 0.573 0.588 0.592

**Fish** 0.424 0.020 0.378 0.387 0.412 0.425 0.437 0.452 0.458

**Terrestrial** 0.012 0.012 0.000 0.001 0.004 0.009 0.017 0.037 0.044


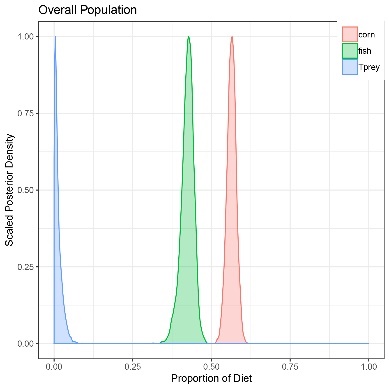


**17^th^ Century:**

**Mean SD 2.5% 5% 25% 50% 75% 95% 97.5%**

**Maize (corn)** 0.581 0.025 0.530 0.539 0.565 0.582 0.597 0.619 0.626

**Fish** 0.368 0.061 0.222 0.252 0.338 0.380 0.410 0.445 0.455

**Terrestrial** 0.051 0.048 0.001 0.002 0.016 0.037 0.072 0.148 0.175


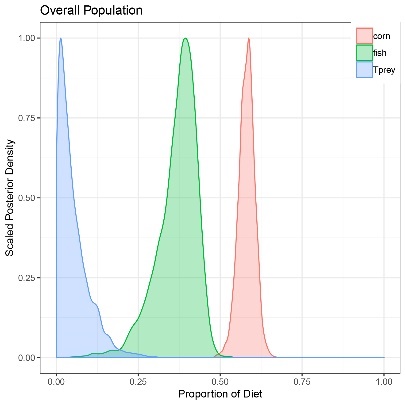


**S1.4.1.1. 3 Source (Maize, Fish, Terrestrial Prey) model on Dentine Collagen for 17^th^ Century excluding Atlantic Salmon (*Salmo salar*) in fish source.**

**17^th^ Century:**

**Mean SD 2.5% 5% 25% 50% 75% 95% 97.5%**

Maize (corn) 0.594 0.024 0.548 0.555 0.579 0.594 0.609 0.633 0.640

Fish 0.328 0.079 0.117 0.170 0.288 0.347 0.385 0.422 0.432

Terrestrial 0.078 0.068 0.002 0.005 0.025 0.060 0.110 0.216 0.260

**S1.4.2. 3 Source (Maize, Fish, Terrestrial Prey) on Bone Collagen by Century**

**14^th^ Century:**

**Mean SD 2.5% 5% 25% 50% 75% 95% 97.5%**

**Maize (corn)** 0.425 0.027 0.367 0.377 0.408 0.426 0.443 0.467 0.475

**Fish** 0.556 0.035 0.486 0.500 0.537 0.558 0.578 0.609 0.619

**Terrestrial** 0.019 0.020 0.000 0.001 0.005 0.013 0.027 0.057 0.069


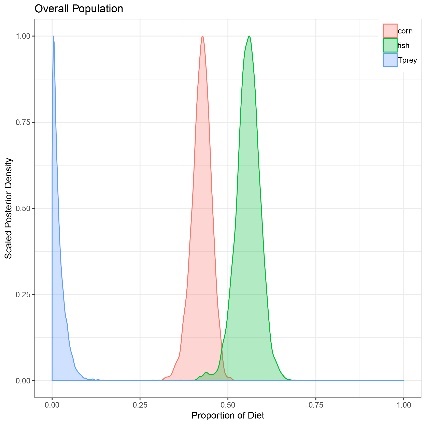


**15^th^ Century:**

**Mean SD 2.5% 5% 25% 50% 75% 95% 97.5%**

**Maize (corn)** 0.470 0.024 0.423 0.431 0.454 0.470 0.485 0.509 0.517

**Fish** 0.450 0.084 0.243 0.285 0.404 0.469 0.513 0.550 0.558

**Terrestrial** 0.080 0.071 0.002 0.004 0.024 0.062 0.118 0.222 0.260


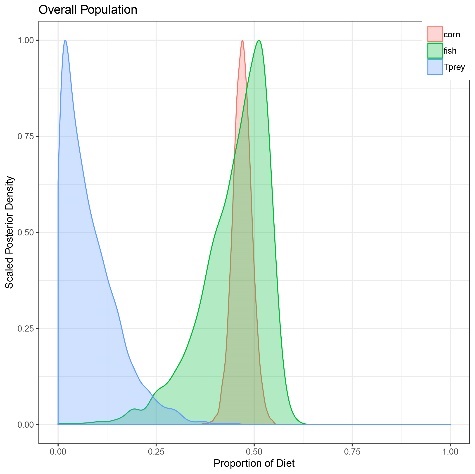


**16^th^ Century:**

**Mean SD 2.5% 5% 25% 50% 75% 95% 97.5%**

**Maize (corn)** 0.474 0.033 0.403 0.420 0.454 0.476 0.495 0.524 0.533

**Fish** 0.435 0.101 0.179 0.232 0.382 0.459 0.509 0.557 0.572

**Terrestrial** 0.092 0.082 0.002 0.004 0.028 0.068 0.134 0.259 0.310


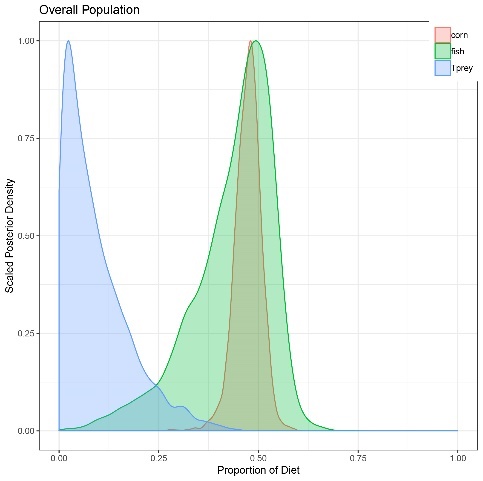


**17^th^ Century:**

**Mean SD 2.5% 5% 25% 50% 75% 95% 97.5%**

**Maize (corn)** 0.469 0.052 0.350 0.387 0.445 0.474 0.501 0.541 0.555

**Fish** 0.384 0.129 0.097 0.141 0.304 0.398 0.481 0.564 0.593

**Terrestrial** 0.147 0.106 0.006 0.011 0.061 0.129 0.212 0.354 0.395


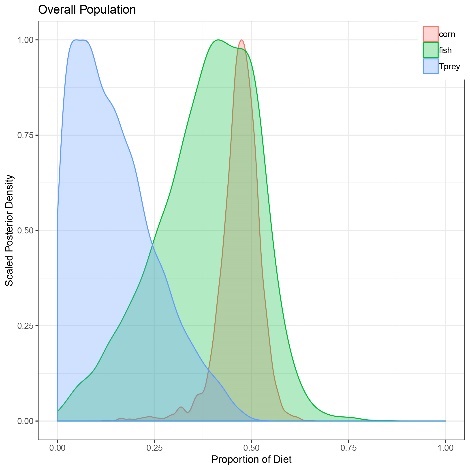


**S1.4.3. Site by Site comparison of bone collagen and dentine collagen**

The results comparing the data by century are largely duplicated in the by-site dentine and bone collagen models as summarized in Fig. S1.4.3. and Table S1.4.3. Departures from the overall trends for the by-century models may be attributed to sampling with many of the sites having <5 samples (Clementz and Koch, 2001) For example Hidden Spring, Damiani, and Milne at the center of the dentine model plot all have <5 samples, with Damiani having a single sample. Milne, which has a single sample, is an obvious outlier in the bone collagen model. Hutchinson early in the sequence and Christian Island at the end of the sequence are also represented by single samples. A plot of the dentine model eliminating sites with <5 samples (Fig. S1.4.3.C) shows the trends clearly. A similar plot for th­­­e bone collagen model would leave only seven sites, with none from the seventeenth century.

Table S1.4.3. Three-source model estimates by individual sites by resource.

| Site | Century | n | Fish | | Maize | | Terrestrial Animal | |
| --- | --- | --- | --- | --- | --- | --- | --- | --- |
|  |  |  | Mean | 95% Range | Mean | 95% Range | Mean | 95% Range |
| Dentine Collagen |  |  |  |  |  |  |  |  |
| Moatfield | Fourteenth | 43 | 0.471±0.022 | 0.425-0.512 | 0.481±0.090 | 0.233-0.611 | 0.011±0.012 | 0.000-0.044 |
| Fairty | Fourteenth | 15 | 0.476±0.056 | 0.363-0.601 | 0.501±0.046 | 0.379-0.568 | 0.023±0.025 | 0.001-0.092 |
| Hutchinson | Fourteenth | 5 | 0.365±0.148 | 0.062-0.683 | 0.481-0.090 | 0.233-0.611 | 0.154±0.112 | 0.007-0.415 |
| Staines | Fourteenth | 15 | 0.477±0.049 | 0.353-0.628 | 0.477±0.049 | 0.378-0.550 | 0.031±0.033 | 0.001-0.121 |
| Damiani | Fifteenth | 1 | 0.329±0.171 | 0.031-0.675 | 0.414-0.123 | 0.113-0.620 | 0.257±0.158 | 0.017-0.603 |
| Hidden Spring | Fifteenth | 4 | 0.357±0.139 | 0.078-0.632 | 0.498±0.085 | 0.268-0.624 | 0.145±0.102 | 0.006-0.375 |
| Bosomworth | Fifteenth | 3 | 0.355±0.188 | 0.038-0.756 | 0.473±0.144 | 0.108-0.669 | 0.172±0.125 | 0.007-0.452 |
| Teston Road | Fifteenth | 10 | 0.417±0.062 | 0.257-0.501 | 0.528±0.024 | 0.475-0.573 | 0.055±0.050 | 0.002-0.189 |
| Uxbridge | Fifteenth | 16 | 0.452±0.042 | 0.356-0.518 | 0.520±0.025 | 0.478-0.564 | 0.028±0.029 | 0.001-0.106 |
| Milne | Sixteenth | 3 | 0.286±0.146 | 0.029-0.619 | 0.559±0.104 | 0.248-0.692 | 0.156±0.110 | 0.008-0.390 |
| Kleinburg | Sixteenth | 16 | 0.393±0.051 | 0.271-0.461 | 0.575±0.031 | 0.529-0.613 | 0.032±0.034 | 0.001-0.127 |
| Mantle Cemetery | Sixteenth | 5 | 0.306±0.114 | 0.060-0.503 | 0.530±0.057 | 0.409-0.632 | 0.164±0.099 | 0.011-0.377 |
| Mantle Village | Sixteenth | 9 | 0.371±0.055 | 0.238-0.457 | 0.582±0.033 | 0.507-0.642 | 0.047±0.043 | 0.001-0.158 |
| Maurice | Seventeenth | 8 | 0.314±0.101 | 0.075-0.459 | 0.576±0.035 | 0.502-0.641 | 0.110±0.085 | 0.003-0.313 |
| Warminster | Seventeenth | 6 | 0.332±0.128 | 0.068-0.630 | 0.550±0.078 | 0.302-0.653 | 0.118±0.089 | 0.004-0.331 |
| Christian Island | Seventeenth | 3 | 0.243±0.150 | 0.021-0.692 | 0.627±0.131 | 0.200-0.777 | 0.129±0.096 | 0.006-0.342 |
| Bone Collagen |  |  |  |  |  |  |  |  |
| Moatfield | Fourteenth | 10 | 0.534±0.066 | 0.287-0.707 | 0.403±0.061 | 0.249-0.499 | 0.063±0.066 | 0.001-0.247 |
| Fairty | Fourteenth | 6 | 0.461±0.121 | 0.174-0.640 | 0.456±0.083 | 0.278-0.596 | 0.084±0.078 | 0.002-0.286 |
| Hutchinson | Fourteenth | 1 | 0.321±0.153 | 0.036-0.614 | 0.456-0.070 | 0.300-0.568 | 0.223±0.133 | 0.014-0.491 |
| Staines | Fourteenth | 4 | 0.386±0.159 | 0.008-0.481 | 0.418±0.083 | 0.225-0.560 | 0.196±0.131 | 0.008-0.481 |
| Hidden Spring | Fifteenth | 2 | 0.470±0.154 | 0.120-0.742 | 0.364±0.084 | 0.162-0.500 | 0.166±0.129 | 0.006-0.493 |
| Teston Road | Fifteenth | 6 | 0.467±0.092 | 0.211-0.566 | 0.457±0.023 | 0.413-0.514 | 0.076±0.078 | 0.002-0.293 |
| Uxbridge | Fifteenth | 7 | 0.386±0.113 | 0.108-0.560 | 0.490±0.047 | 0.391-0.572 | 0.124±0.094 | 0.006-0.358 |
| Milne | Sixteenth | 1 | 0.685±0.113 | 0.485-0.918 | 0.272±0.111 | 0.040-0.475 | 0.043±0.038 | 0.001-0.139 |
| Kleinburg | Sixteenth | 9 | 0.507±0.121 | 0.209-0.729 | 0.425±0.078 | 0.226-0.523 | 0.068±0.073 | 0.001-0.278 |
| Maurice | Seventeenth | 2 | 0.393±0.196 | 0.036-0.785 | 0.367±0.132 | 0.053-0.578 | 0.240±0.166 | 0.012-0.610 |
| Warminster | Seventeenth | 3 | 0.334±0.156 | 0.034-0.631 | 0.461±0.097 | 0.213-0.609 | 0.204±0.130 | 0.012-0.482 |
| Christian Island | Seventeenth | 1 | 0.379±0.119 | 0.105-0.604 | 0.511±0.074 | 0.338-0.631 | 0.110±0.091 | 0.003-0.354 |

**
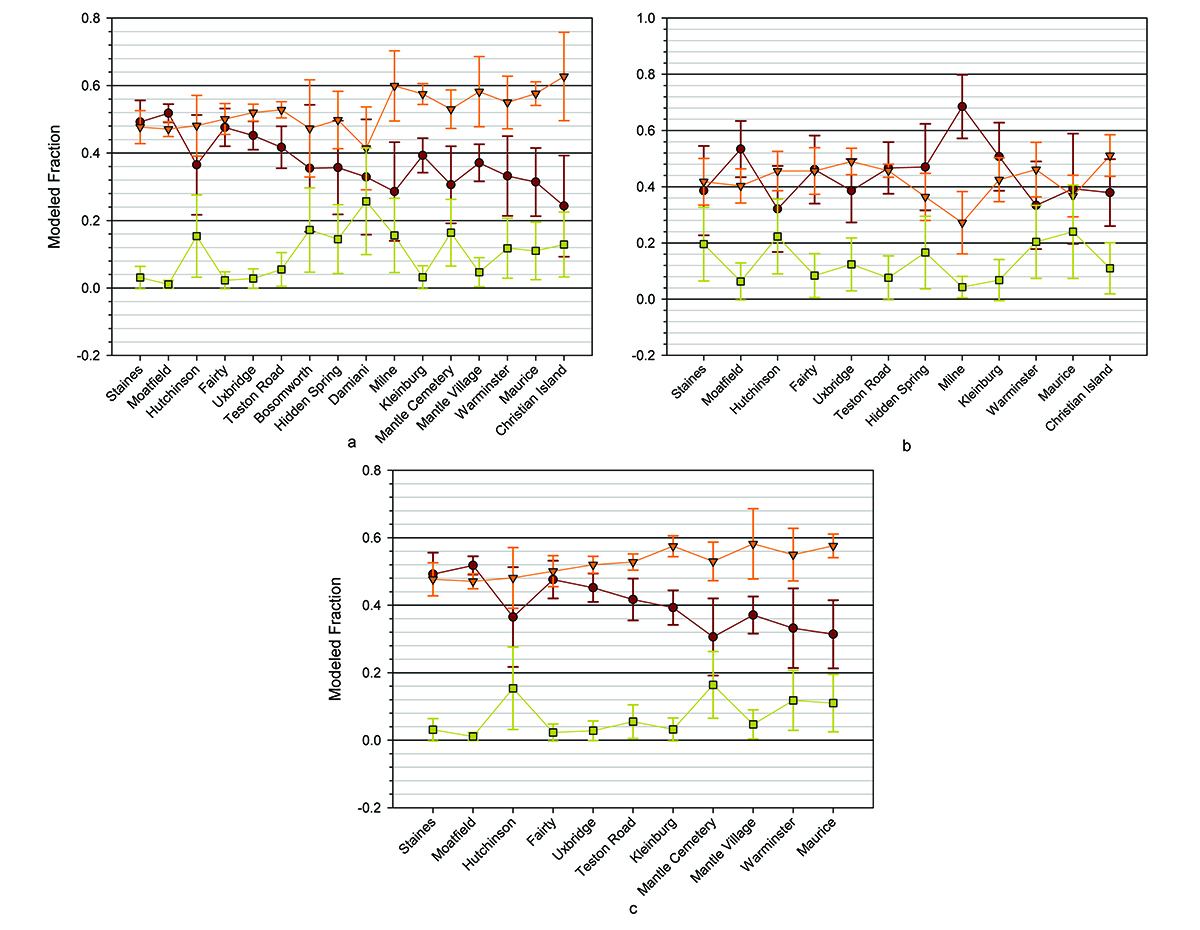
**

Figure 1.4.3. 3 source models of proportion of food in diet by site. A, models using dentine collagen isotope values; B, models using bone collagen isotope values; C, dentine collagen models excluding sites <5 samples. Symbols: orange triangle, maize; red circle, fish; green square, terrestrial prey.

**S1.4.4. 3 Source Models (Maize, Fish, Terrestrial Prey) on Dentine Collagen by Locality**

**Staines**:

**Mean SD 2.5% 5% 25% 50% 75% 95% 97.5%**

**Maize (corn)** 0.477 0.049 0.350 0.378 0.454 0.486 0.510 0.541 0.550

**Fish** 0.492 0.064 0.353 0.384 0.457 0.492 0.528 0.600 0.628

**Terrestrial** 0.031 0.033 0.001 0.001 0.008 0.020 0.041 0.100 0.121


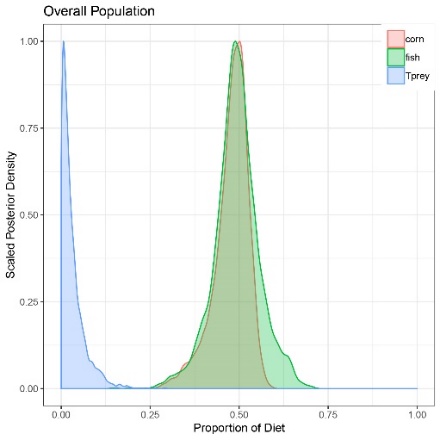


**Moatfield**:

**Mean SD 2.5% 5% 25% 50% 75% 95% 97.5%**

**Maize (corn)** 0.471 0.022 0.425 0.434 0.457 0.472 0.487 0.505 0.512

**Fish** 0.518 0.027 0.464 0.473 0.502 0.518 0.535 0.560 0.568

**Terrestrial** 0.011 0.012 0.000 0.001 0.003 0.007 0.016 0.036 0.044


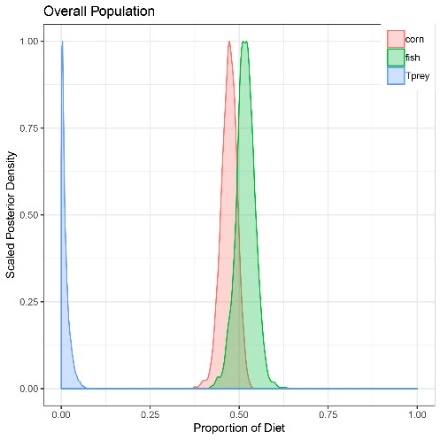


**Hutchinson**:

**Mean SD 2.5% 5% 25% 50% 75% 95% 97.5%**

**Maize (corn)** 0.481 0.090 0.233 0.316 0.449 0.497 0.534 0.588 0.611

**Fish** 0.365 0.148 0.062 0.111 0.273 0.369 0.457 0.594 0.683

**Terrestrial** 0.154 0.112 0.007 0.014 0.064 0.131 0.222 0.366 0.415


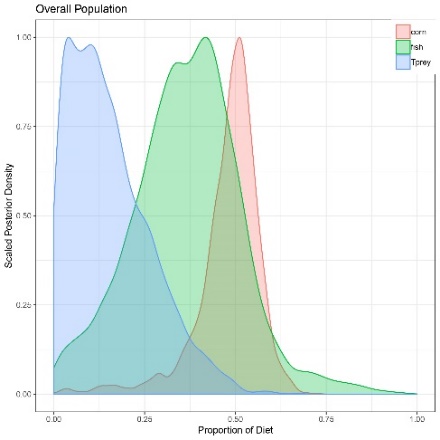


**Fairty**:

**Mean SD 2.5% 5% 25% 50% 75% 95% 97.5%**

**Maize (corn)** 0.501 0.046 0.379 0.407 0.480 0.508 0.532 0.561 0.568

**Fish** 0.476 0.056 0.363 0.386 0.445 0.474 0.506 0.578 0.601

**Terrestrial** 0.023 0.025 0.001 0.001 0.006 0.015 0.032 0.074 0.092


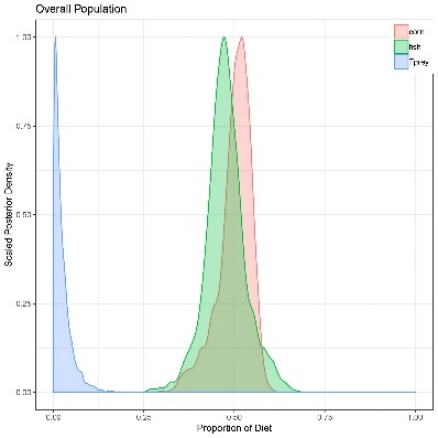


**Uxbridge**:

**Mean SD 2.5% 5% 25% 50% 75% 95% 97.5%**

**Maize (corn)** 0.520 0.025 0.468 0.478 0.505 0.521 0.536 0.558 0.564

**Fish** 0.452 0.042 0.356 0.379 0.433 0.457 0.478 0.507 0.518

**Terrestrial** 0.028 0.029 0.001 0.002 0.008 0.019 0.038 0.084 0.106


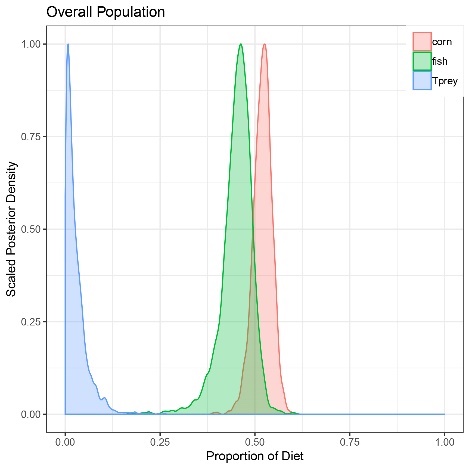


**Teston**:

**Mean SD 2.5% 5% 25% 50% 75% 95% 97.5%**

**Maize (corn)** 0.528 0.024 0.475 0.488 0.514 0.529 0.543 0.565 0.573

**Fish** 0.417 0.062 0.257 0.299 0.390 0.430 0.460 0.492 0.501

**Terrestrial** 0.055 0.050 0.002 0.004 0.018 0.040 0.076 0.154 0.189


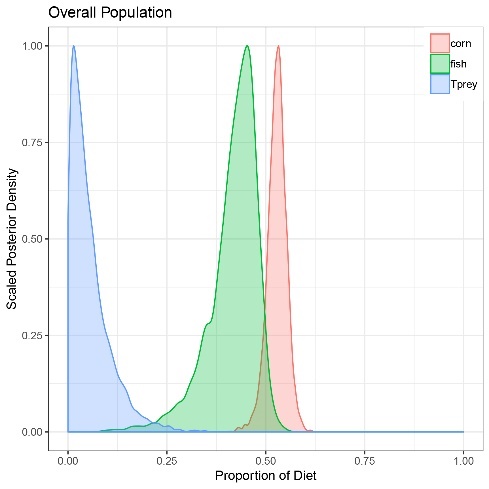


**Bosomworth**:

**Mean SD 2.5% 5% 25% 50% 75% 95% 97.5%**

**Maize (corn)** 0.473 0.144 0.108 0.168 0.410 0.510 0.574 0.646 0.669

**Fish** 0.355 0.188 0.038 0.067 0.224 0.339 0.461 0.721 0.756

**Terrestrial** 0.172 0.125 0.007 0.014 0.070 0.150 0.248 0.403 0.452


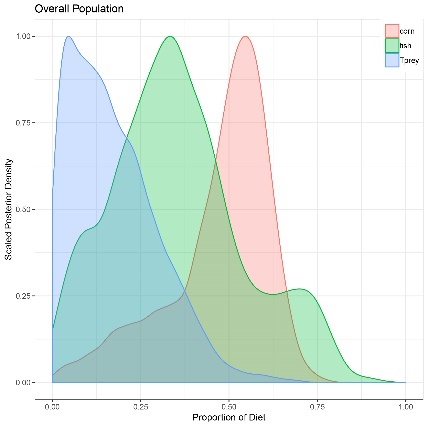


**Hidden Spring**:

**Mean SD 2.5% 5% 25% 50% 75% 95% 97.5%**

**Maize (corn)** 0.498 0.085 0.268 0.325 0.464 0.514 0.552 0.604 0.624

**Fish** 0.357 0.139 0.078 0.119 0.262 0.363 0.454 0.583 0.632

**Terrestrial** 0.145 0.102 0.006 0.012 0.061 0.129 0.214 0.334 0.375


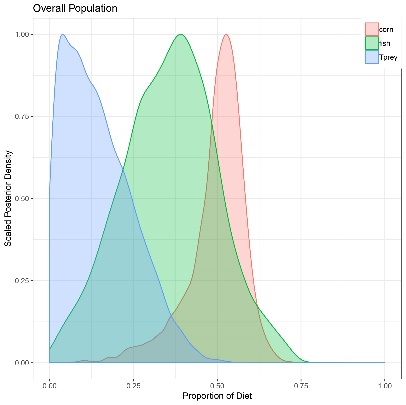


**Damiani**:

**Mean SD 2.5% 5% 25% 50% 75% 95% 97.5%**

**Maize (corn)** 0.414 0.123 0.113 0.162 0.352 0.433 0.496 0.586 0.620

**Fish** 0.329 0.171 0.031 0.056 0.202 0.325 0.446 0.618 0.675

**Terrestrial** 0.257 0.158 0.017 0.029 0.132 0.243 0.358 0.539 0.603

**
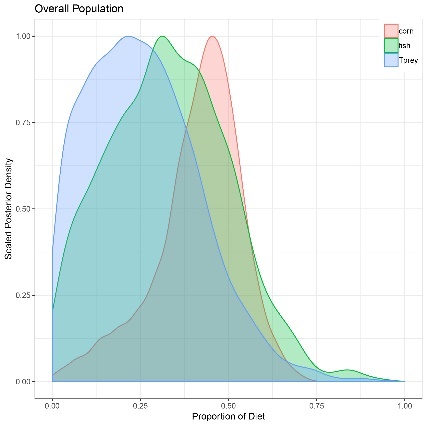
**

**Mantle** **Cemetery**:

**Mean SD 2.5% 5% 25% 50% 75% 95% 97.5%**

**Maize (corn)** 0.530 0.057 0.409 0.432 0.499 0.535 0.567 0.614 0.632

**Fish** 0.306 0.114 0.060 0.101 0.231 0.318 0.391 0.475 0.503

**Terrestrial** 0.164 0.099 0.011 0.021 0.085 0.155 0.229 0.345 0.377


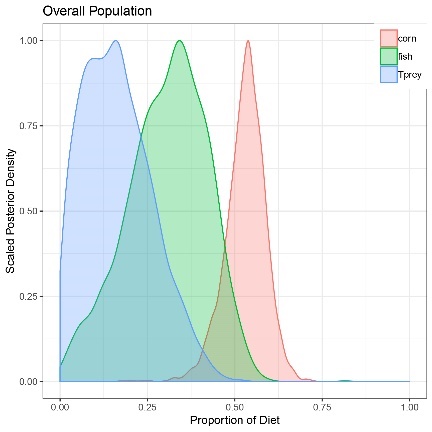


**Mantle** **Village**:

**Mean SD 2.5% 5% 25% 50% 75% 95% 97.5%**

**Maize (corn)** 0.582 0.033 0.507 0.524 0.563 0.584 0.604 0.632 0.642

**Fish** 0.371 0.055 0.238 0.268 0.343 0.380 0.408 0.444 0.457

**Terrestrial** 0.047 0.043 0.001 0.002 0.015 0.035 0.066 0.130 0.158


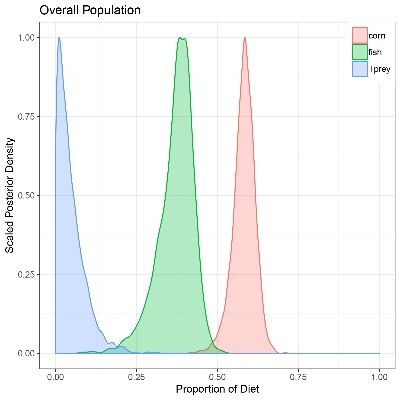


**Milne**:

**Mean SD 2.5% 5% 25% 50% 75% 95% 97.5%**

**Maize (corn)** 0.559 0.104 0.248 0.340 0.532 0.584 0.619 0.673 0.692

**Fish** 0.286 0.146 0.029 0.050 0.181 0.285 0.376 0.530 0.619

**Terrestrial** 0.156 0.110 0.008 0.013 0.066 0.137 0.229 0.350 0.390


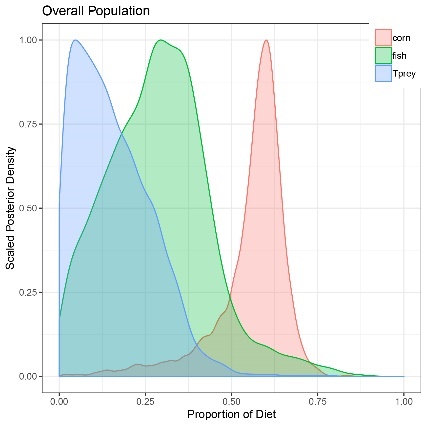


**Kleinburg**:

**Mean SD 2.5% 5% 25% 50% 75% 95% 97.5%**

**Maize (corn)** 0.575 0.031 0.529 0.542 0.566 0.578 0.590 0.607 0.613

**Fish** 0.393 0.051 0.271 0.303 0.372 0.401 0.421 0.447 0.461

**Terrestrial** 0.032 0.034 0.001 0.001 0.008 0.020 0.043 0.102 0.127


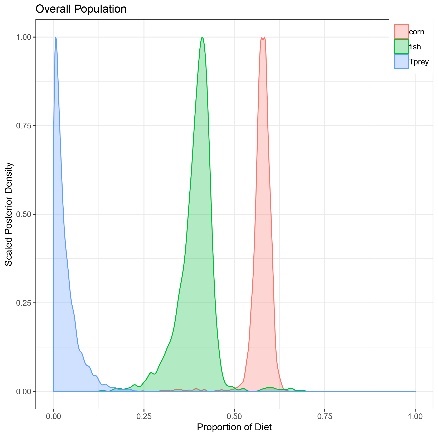


**Warminster**:

**Mean SD 2.5% 5% 25% 50% 75% 95% 97.5%**

**Maize (corn)** 0.550 0.078 0.302 0.393 0.531 0.564 0.594 0.635 0.653

**Fish** 0.332 0.128 0.068 0.113 0.253 0.336 0.408 0.545 0.630

**Terrestrial** 0.118 0.089 0.004 0.009 0.045 0.102 0.170 0.292 0.331


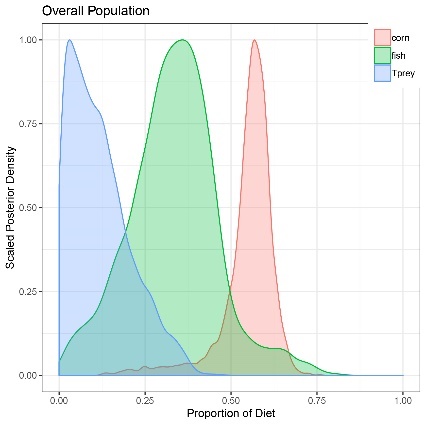


**Maurice:**

**Mean SD 2.5% 5% 25% 50% 75% 95% 97.5%**

**Maize (corn)** 0.576 0.035 0.502 0.519 0.557 0.578 0.598 0.629 0.641

**Fish** 0.314 0.101 0.075 0.117 0.255 0.330 0.391 0.444 0.459

**Terrestrial** 0.110 0.085 0.003 0.007 0.041 0.092 0.160 0.280 0.313


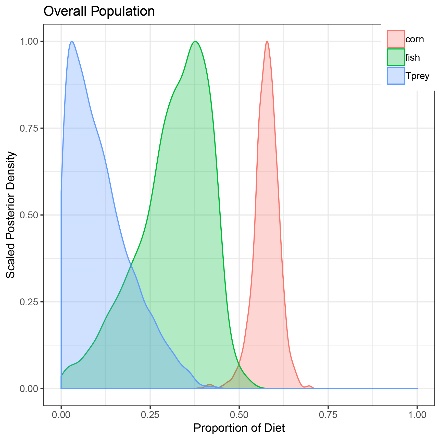


**Christian Island**:

**Mean SD 2.5% 5% 25% 50% 75% 95% 97.5%**

**Maize (corn)** 0.627 0.131 0.200 0.337 0.599 0.664 0.702 0.758 0.777

**Fish** 0.243 0.150 0.021 0.037 0.144 0.228 0.307 0.536 0.692

**Terrestrial** 0.129 0.096 0.006 0.011 0.054 0.113 0.186 0.299 0.342


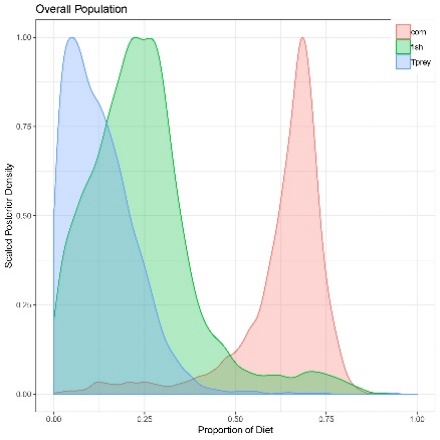


**S1.4.5. 3 Source Models (Maize, Fish, Terrestrial Prey) on Bone Collagen by Locality**

**Staines**:

**Mean SD 2.5% 5% 25% 50% 75% 95% 97.5%**

**Maize (corn)** 0.418 0.083 0.225 0.262 0.377 0.428 0.470 0.535 0.560

**Fish** 0.386 0.159 0.066 0.107 0.273 0.394 0.506 0.625 0.666

**Terrestrial** 0.196 0.131 0.008 0.019 0.089 0.178 0.281 0.438 0.481


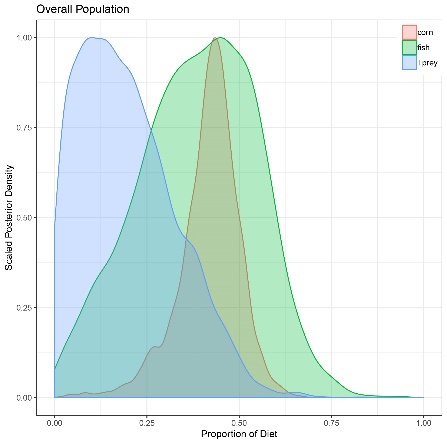


**Moatfield**:

**Mean SD 2.5% 5% 25% 50% 75% 95% 97.5%**

**Maize (corn)** 0.403 0.061 0.249 0.287 0.374 0.413 0.443 0.485 0.499

**Fish** 0.534 0.100 0.287 0.338 0.487 0.546 0.594 0.683 0.707

**Terrestrial** 0.063 0.066 0.001 0.003 0.018 0.041 0.085 0.206 0.247


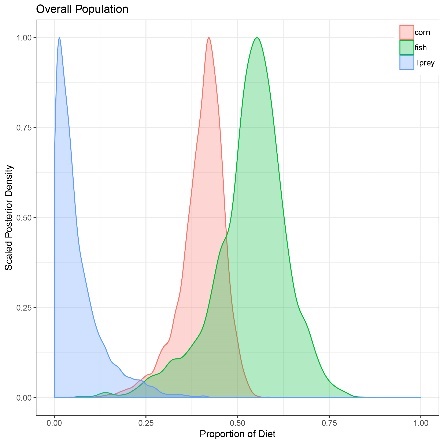


**Hutchinson**:

**Mean SD 2.5% 5% 25% 50% 75% 95% 97.5%**

**Maize (corn)** 0.456 0.070 0.300 0.332 0.417 0.463 0.504 0.555 0.568

**Fish** 0.321 0.153 0.036 0.065 0.213 0.323 0.431 0.573 0.614

**Terrestrial** 0.223 0.133 0.014 0.029 0.116 0.210 0.318 0.459 0.491


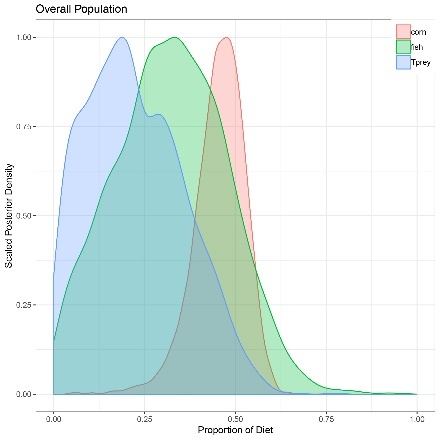


**Mean SD 2.5% 5% 25% 50% 75% 95% 97.5%**

**Maize (corn)** 0.456 0.083 0.278 0.309 0.399 0.464 0.517 0.575 0.596

**Fish** 0.461 0.121 0.174 0.227 0.395 0.479 0.548 0.622 0.640

**Terrestrial** 0.084 0.078 0.002 0.004 0.024 0.060 0.121 0.238 0.286

**
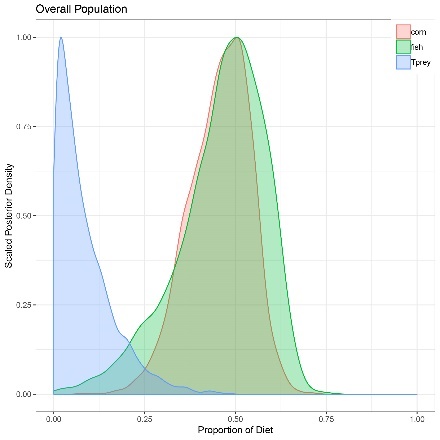
**

**Uxbridge**:

**Mean SD 2.5% 5% 25% 50% 75% 95% 97.5%**

**Maize (corn)** 0.490 0.047 0.391 0.417 0.467 0.494 0.518 0.557 0.572

**Fish** 0.386 0.113 0.108 0.162 0.324 0.403 0.467 0.535 0.560

**Terrestrial** 0.124 0.094 0.006 0.011 0.052 0.105 0.174 0.320 0.358


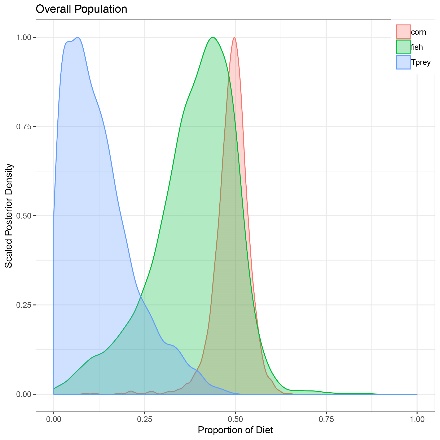


**Teston:**

**Mean SD 2.5% 5% 25% 50% 75% 95% 97.5%**

**Maize (corn)** 0.457 0.023 0.413 0.422 0.444 0.456 0.470 0.495 0.504

**Fish** 0.467 0.092 0.211 0.274 0.434 0.497 0.530 0.558 0.566

**Terrestrial** 0.076 0.078 0.002 0.005 0.021 0.050 0.104 0.241 0.293

**
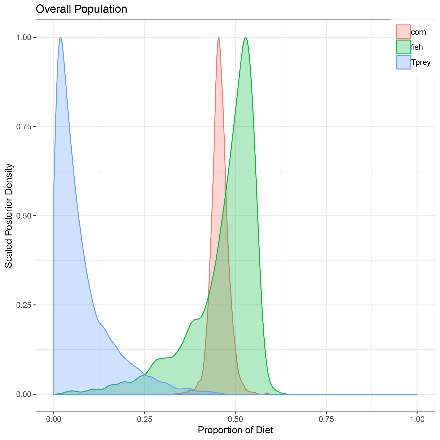
**

**Hidden Spring**:

**Mean SD 2.5% 5% 25% 50% 75% 95% 97.5%**

**Maize (corn)** 0.364 0.084 0.162 0.208 0.316 0.375 0.422 0.482 0.500

**Fish** 0.470 0.154 0.120 0.191 0.380 0.477 0.573 0.701 0.742

**Terrestrial** 0.166 0.129 0.006 0.014 0.068 0.140 0.233 0.423 0.493

**
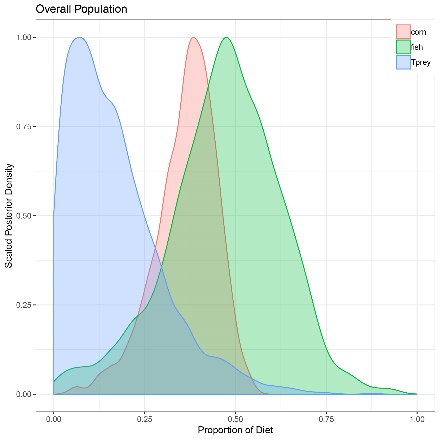
**

**Milne**:

**Mean SD 2.5% 5% 25% 50% 75% 95% 97.5%**

**Maize (corn)** 0.272 0.111 0.040 0.073 0.195 0.282 0.352 0.443 0.475

**Fish** 0.685 0.113 0.485 0.511 0.604 0.678 0.763 0.885 0.918

**Terrestrial** 0.043 0.038 0.001 0.002 0.013 0.032 0.061 0.120 0.139


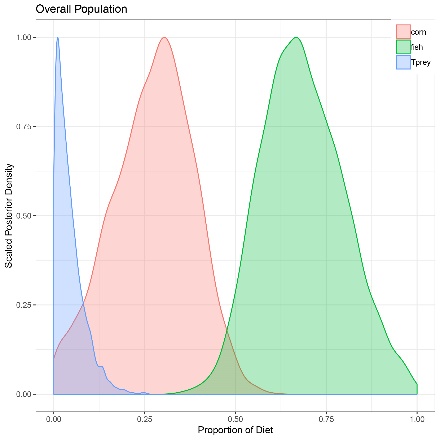


**Kleinburg:**

**Mean SD 2.5% 5% 25% 50% 75% 95% 97.5%**

**Maize (corn)** 0.425 0.078 0.226 0.253 0.400 0.447 0.476 0.513 0.523

**Fish** 0.507 0.121 0.209 0.291 0.446 0.515 0.572 0.706 0.729

**Terrestrial** 0.068 0.073 0.001 0.003 0.017 0.043 0.093 0.222 0.278

**
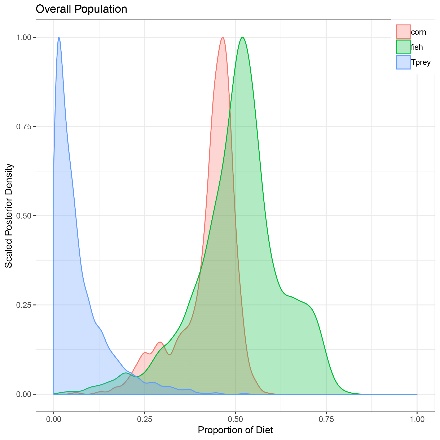
**

**Warminster**:

**Mean SD 2.5% 5% 25% 50% 75% 95% 97.5%**

**Maize (corn)** 0.461 0.097 0.213 0.265 0.422 0.479 0.522 0.586 0.609

**Fish** 0.334 0.156 0.034 0.066 0.224 0.340 0.445 0.589 0.631

**Terrestrial** 0.204 0.130 0.012 0.023 0.100 0.187 0.292 0.430 0.482


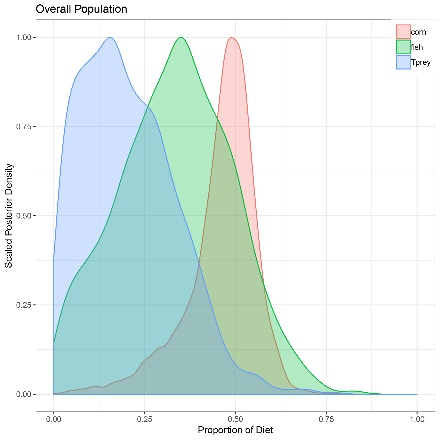


**Maurice**:

**Mean SD 2.5% 5% 25% 50% 75% 95% 97.5%**

**Maize (corn)** 0.367 0.132 0.053 0.100 0.293 0.394 0.459 0.549 0.578

**Fish** 0.393 0.196 0.036 0.066 0.250 0.397 0.528 0.718 0.785

**Terrestrial** 0.240 0.166 0.012 0.023 0.105 0.213 0.346 0.539 0.610


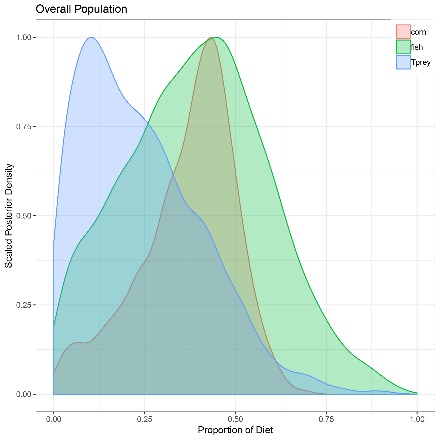


**Christian Island:**

**Mean SD 2.5% 5% 25% 50% 75% 95% 97.5%**

**Maize (corn)** 0.511 0.074 0.338 0.379 0.470 0.521 0.563 0.613 0.631

**Fish** 0.379 0.119 0.105 0.164 0.314 0.386 0.456 0.565 0.604

**Terrestrial** 0.110 0.091 0.003 0.007 0.041 0.088 0.152 0.290 0.354


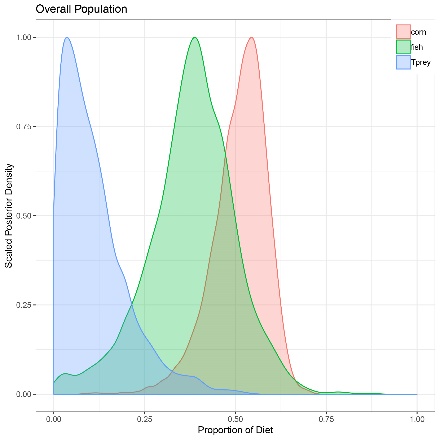


**S1.4.6. 5 Source Models (Maize, Terrestrial Prey, High N Fish, Medium N Fish, Low N Fish) from Dentine Collagen by Century**

**14^th^ Century**:

**Mean SD 2.5% 5% 25% 50% 75% 95% 97.5%**

**Maize** 0.483 0.014 0.454 0.460 0.474 0.483 0.493 0.505 0.510

**HighNFish** 0.490 0.020 0.448 0.456 0.478 0.492 0.504 0.519 0.524

**LowNFish** 0.008 0.008 0.000 0.001 0.003 0.006 0.012 0.024 0.028

**MidNFish** 0.014 0.012 0.001 0.001 0.005 0.010 0.019 0.039 0.047

**Terrestrial** 0.005 0.005 0.000 0.000 0.001 0.003 0.007 0.014 0.017


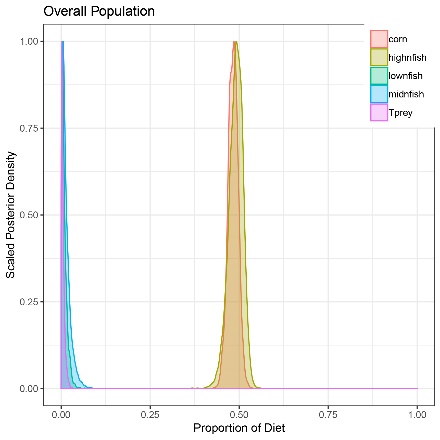


**15^th^ Century:**

**Mean SD 2.5% 5% 25% 50% 75% 95% 97.5%**

**Maize**  **(corn)** 0.515 0.013 0.488 0.493 0.506 0.515 0.524 0.537 0.541

**HighNFish** 0.412 0.038 0.322 0.340 0.391 0.417 0.440 0.466 0.474

**LowNFish** 0.021 0.019 0.000 0.001 0.006 0.016 0.030 0.059 0.072

**MidNFish** 0.039 0.034 0.001 0.002 0.013 0.029 0.056 0.108 0.126

**Terrestrial** 0.013 0.012 0.000 0.001 0.004 0.010 0.019 0.039 0.046


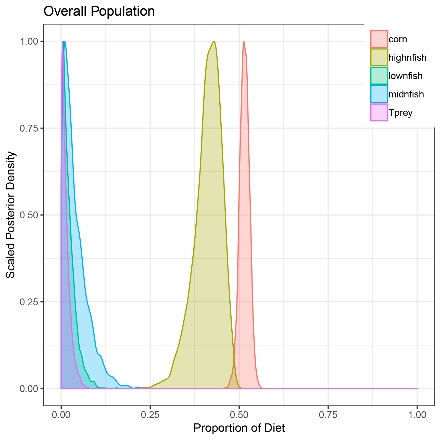


**16^th^ Century:**

**Mean SD 2.5% 5% 25% 50% 75% 95% 97.5%**

**Maize**  **(corn)** 0.567 0.014 0.539 0.544 0.558 0.567 0.576 0.589 0.593

**HighNFish** 0.369 0.037 0.278 0.299 0.349 0.374 0.395 0.419 0.427

**LowNFish** 0.019 0.018 0.001 0.001 0.006 0.014 0.027 0.055 0.067

**MidNFish** 0.033 0.031 0.001 0.002 0.010 0.024 0.047 0.094 0.117

**Terrestrial** 0.012 0.011 0.000 0.001 0.004 0.009 0.017 0.034 0.042


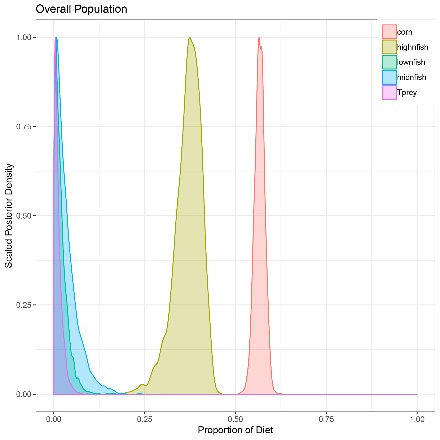


**17^th^ Century:**

**Mean SD 2.5% 5% 25% 50% 75% 95% 97.5%**

**Maize** **(corn)** 0.573 0.025 0.520 0.531 0.559 0.574 0.589 0.611 0.617

**HighNFish** 0.286 0.078 0.110 0.141 0.240 0.293 0.342 0.397 0.412

**LowNFish** 0.044 0.040 0.001 0.003 0.014 0.032 0.061 0.123 0.146

**MidNFish** 0.070 0.061 0.001 0.003 0.022 0.053 0.101 0.191 0.226

**Terrestrial** 0.028 0.028 0.001 0.001 0.007 0.019 0.040 0.086 0.105

**
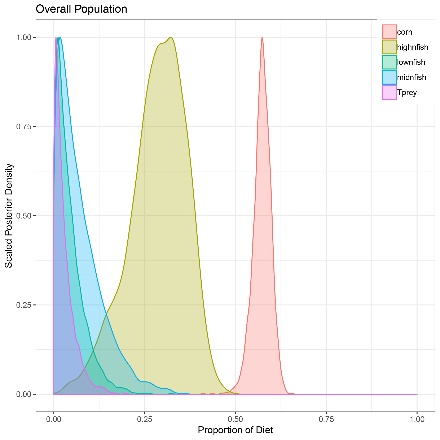
**

**S1.4.6.1. 5 Source (Maize, Terrestrial Prey, High N Fish, Medium N Fish, Low N Fish) model on Dentine Collagen for 17^th^ Century excluding Atlantic Salmon (*Salmo salar*) in fish source.**

**17^th^ Century:**

**Mean SD 2.5% 5% 25% 50% 75% 95% 97.5%**

**Maize (corn)** 0.584 0.024 0.531 0.542 0.570 0.586 0.601 0.621 0.628

**HighNFish** 0.270 0.081 0.067 0.110 0.226 0.280 0.330 0.383 0.398

**LowNFish** 0.044 0.041 0.002 0.003 0.013 0.032 0.062 0.127 0.152

**MidNFish** 0.074 0.064 0.003 0.006 0.026 0.057 0.102 0.202 0.241

**Terrestrial** 0.028 0.029 0.001 0.001 0.007 0.019 0.040 0.087 0.104

**S1.4.7. 5 Source Models (Maize, Terrestrial Prey, High N Fish, Medium N Fish, Low N Fish) from Bone Collagen by Century**

**14^th^ Century:**

**Mean SD 2.5% 5% 25% 50% 75% 95% 97.5%**

**Maize**  **(corn)** 0.433 0.024 0.385 0.394 0.417 0.433 0.449 0.471 0.477

**HighNFish** 0.467 0.054 0.344 0.369 0.437 0.475 0.505 0.542 0.551

**LowNFish** 0.028 0.027 0.001 0.001 0.008 0.021 0.040 0.081 0.097

**MidNFish** 0.051 0.047 0.002 0.003 0.016 0.038 0.073 0.145 0.176

**Terrestrial** 0.020 0.018 0.001 0.001 0.007 0.015 0.029 0.056 0.068


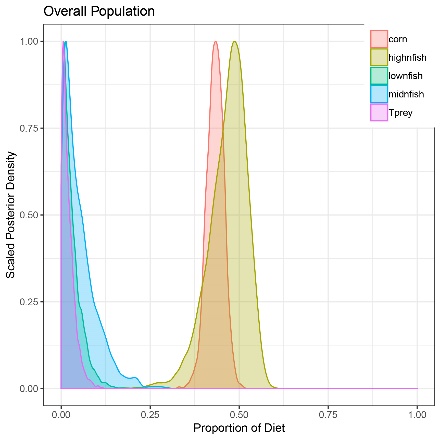


**15^th^ Century:**

**Mean SD 2.5% 5% 25% 50% 75% 95% 97.5%**

**Maize**  **(corn)** 0.463 0.021 0.418 0.426 0.450 0.463 0.476 0.496 0.505

**HighNFish** 0.323 0.102 0.085 0.132 0.262 0.335 0.397 0.469 0.489

**LowNFish** 0.064 0.057 0.002 0.004 0.021 0.048 0.091 0.175 0.206

**MidNFish** 0.102 0.083 0.003 0.007 0.037 0.080 0.146 0.261 0.306

**Terrestrial** 0.049 0.044 0.002 0.003 0.015 0.036 0.069 0.140 0.169


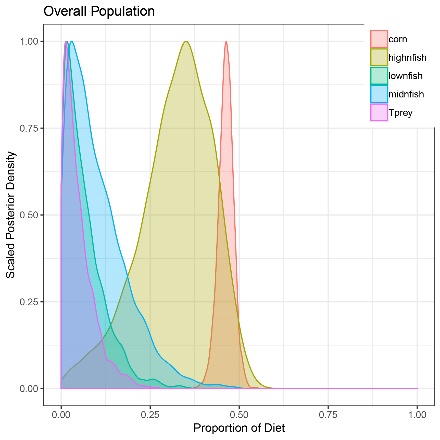


**16^th^ Century:**

**Mean SD 2.5% 5% 25% 50% 75% 95% 97.5%**

**Maize**  **(corn)** 0.460 0.035 0.381 0.403 0.443 0.464 0.482 0.508 0.516

**HighNFish** 0.316 0.111 0.069 0.110 0.246 0.325 0.397 0.480 0.502

**LowNFish** 0.069 0.063 0.002 0.004 0.022 0.051 0.098 0.190 0.232

**MidNFish** 0.104 0.084 0.003 0.007 0.037 0.084 0.150 0.272 0.312

**Terrestrial** 0.051 0.048 0.001 0.003 0.016 0.037 0.071 0.149 0.172


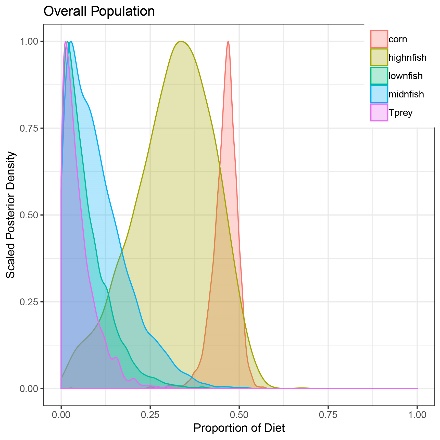


**17^th^ Century:**

**Mean SD 2.5% 5% 25% 50% 75% 95% 97.5%**

**Maize**  **(corn)** 0.439 0.063 0.280 0.322 0.416 0.450 0.476 0.515 0.532

**HighNFish** 0.260 0.123 0.032 0.051 0.173 0.264 0.348 0.460 0.490

**LowNFish** 0.097 0.080 0.004 0.007 0.035 0.075 0.140 0.256 0.300

**MidNFish** 0.127 0.099 0.005 0.009 0.049 0.106 0.183 0.314 0.364

**Terrestrial** 0.076 0.067 0.002 0.005 0.025 0.057 0.110 0.210 0.250


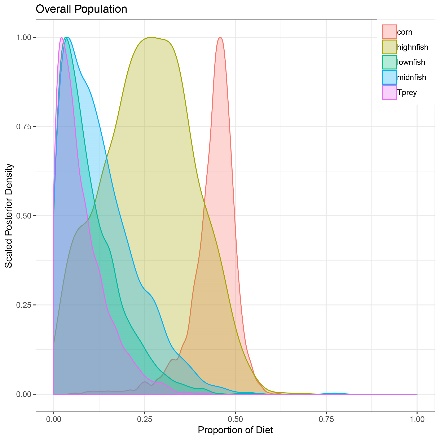


**S1.4.8. 3 Source Models (Maize, Fish, Terrestrial Prey) on Tooth enamel conversion of Dentine collagen δ^13^C by century**

**14^th^ Century:**

**Mean SD 2.5% 5% 25% 50% 75% 95% 97.5%**

**Maize**  **(corn)** 0.577 0.017 0.542 0.548 0.566 0.578 0.589 0.605 0.609

**Fish** 0.418 0.018 0.383 0.388 0.405 0.417 0.429 0.448 0.455

**Terrestrial** 0.005 0.005 0.000 0.000 0.001 0.004 0.007 0.016 0.019


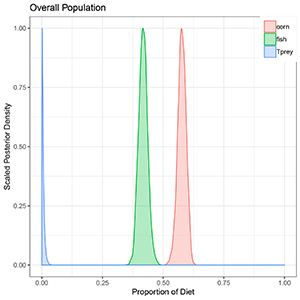


**15^th^ Century:**

**Mean SD 2.5% 5% 25% 50% 75% 95% 97.5%**

**Maize**  **(corn)** 0.634 0.020 0.593 0.601 0.621 0.635 0.647 0.665 0.671

**Fish** 0.356 0.024 0.307 0.317 0.341 0.356 0.371 0.393 0.402

**Terrestrial** 0.010 0.011 0.000 0.001 0.003 0.007 0.015 0.032 0.040


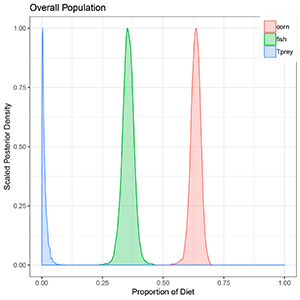


**16^th^ Century:**

**Mean SD 2.5% 5% 25% 50% 75% 95% 97.5%**

**Maize**  **(corn)** 0.632 0.020 0.588 0.596 0.620 0.632 0.646 0.663 0.668

**Fish** 0.356 0.025 0.302 0.314 0.341 0.357 0.372 0.395 0.404

**Terrestrial** 0.012 0.013 0.000 0.001 0.003 0.008 0.016 0.037 0.046


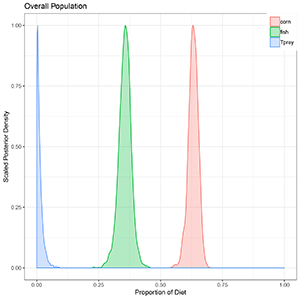


**17^th^ Century**

**Mean SD 2.5% 5% 25% 50% 75% 95% 97.5%**

**Maize**  **(corn)** 0.754 0.019 0.717 0.723 0.742 0.754 0.766 0.784 0.791

**Fish** 0.204 0.049 0.077 0.104 0.179 0.215 0.238 0.264 0.270

**Terrestrial** 0.043 0.040 0.001 0.002 0.012 0.031 0.062 0.124 0.150


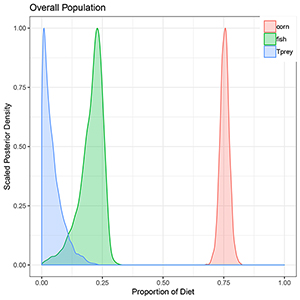


**S1.4.9. 3 Source Models (Maize, Fish, Terrestrial Prey) on Tooth enamel conversion of Bone Collagen δ^13^C by century**

**14^th^ Century:**

Mean SD 2.5% 5% 25% 50% 75% 95% 97.5%

**Maize**  **(corn)** 0.578 0.041 0.479 0.507 0.558 0.584 0.605 0.634 0.642

**Fish** 0.398 0.052 0.293 0.315 0.369 0.398 0.427 0.479 0.506

**Terrestrial** 0.024 0.025 0.001 0.001 0.006 0.015 0.032 0.075 0.096


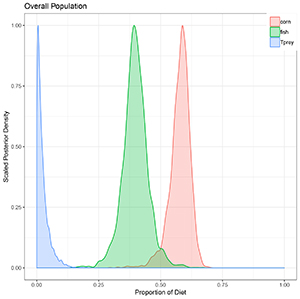


**15^th^ Century:**

**Mean SD 2.5% 5% 25% 50% 75% 95% 97.5%**

**Maize**  **(corn)** 0.696 0.031 0.631 0.644 0.677 0.697 0.716 0.745 0.753

**Fish** 0.252 0.062 0.095 0.132 0.221 0.262 0.295 0.333 0.348

**Terrestrial** 0.052 0.048 0.001 0.003 0.016 0.038 0.075 0.152 0.180


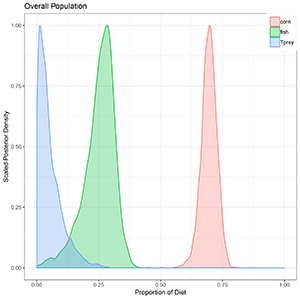


**16^th^ Century:**

**Mean SD 2.5% 5% 25% 50% 75% 95% 97.5%**

**Maize**  **(corn)** 0.675 0.028 0.615 0.629 0.659 0.676 0.694 0.719 0.726

**Fish** 0.255 0.070 0.081 0.118 0.214 0.267 0.305 0.346 0.358

**Terrestrial** 0.070 0.058 0.003 0.005 0.024 0.056 0.103 0.190 0.219


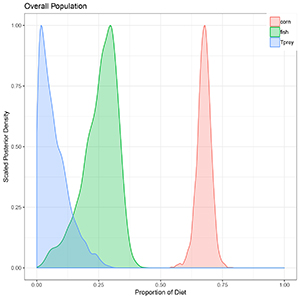


**17^th^ Century:**

**Mean SD 2.5% 5% 25% 50% 75% 95% 97.5%**

**Maize**  **(corn)** 0.750 0.050 0.632 0.664 0.726 0.756 0.781 0.816 0.827

**Fish** 0.173 0.078 0.020 0.035 0.120 0.178 0.224 0.289 0.323

**Terrestrial** 0.077 0.058 0.003 0.006 0.030 0.065 0.113 0.191 0.211


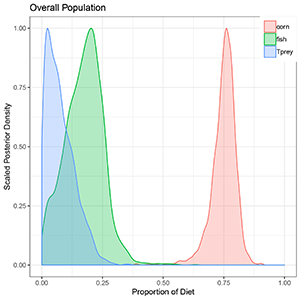


**S2. Isotope data used as sources in the MixSIAR models**

Sample numbers, taxon, stable isotope values, reference from which data derive, as well as the source group for the different models that were run in this study. Maize values below have been adjusted according to the maize roasting experiment explained in the text.

| **Sample** | **Species** | **d15N** | **d13C** | **Reference** | **3 Source Group** | **5 Source Group** |
| --- | --- | --- | --- | --- | --- | --- |
| DOR04.1 | bear | 5.58 | -24.03 | Booth (2014) | Terrestrial Species | Terrestrial Species |
| DOR05.1 | bear | 6.38 | -22.83 | Booth (2014) | Terrestrial Species | Terrestrial Species |
| DOR06.1 | bear | 7.61 | -22.64 | Booth (2014) | Terrestrial Species | Terrestrial Species |
| DOR07.1 | bear | 5.34 | -25.02 | Booth (2014) | Terrestrial Species | Terrestrial Species |
| DOR08.1 | bear | 6.68 | -23.96 | Booth (2014) | Terrestrial Species | Terrestrial Species |
| DOR09.1 | bear | 7.38 | -23.09 | Booth (2014) | Terrestrial Species | Terrestrial Species |
| DOR10.1 | bear | 6.64 | -23.62 | Booth (2014) | Terrestrial Species | Terrestrial Species |
| HOL02.1 | bear | 5.63 | -22.8 | Booth (2014) | Terrestrial Species | Terrestrial Species |
| HOL16.1 | bear | 3.77 | -21.25 | Booth (2014) | Terrestrial Species | Terrestrial Species |
| WIA02.1 | bear | 5.68 | -20.44 | Booth (2014) | Terrestrial Species | Terrestrial Species |
| CLV04.1 | bear | 7.88 | -20.58 | Booth (2014) | Terrestrial Species | Terrestrial Species |
| CLV06.1 | bear | 4.99 | -22.44 | Booth (2014) | Terrestrial Species | Terrestrial Species |
| MCK01.1 | bear | 4.75 | -20.02 | Booth (2014) | Terrestrial Species | Terrestrial Species |
| MCK07.1 | bear | 4.89 | -22.6 | Booth (2014) | Terrestrial Species | Terrestrial Species |
| MCK15.1 | bear | 3.43 | -20.29 | Booth (2014) | Terrestrial Species | Terrestrial Species |
| MCK16.12 | bear | 4.66 | -19.87 | Booth (2014) | Terrestrial Species | Terrestrial Species |
| MCK25.1 | bear | 5.37 | -20.54 | Booth (2014) | Terrestrial Species | Terrestrial Species |
| MCK27.1 | bear | 5.17 | -20.43 | Booth (2014) | Terrestrial Species | Terrestrial Species |
| MCK28.1 | bear | 5.45 | -21.76 | Booth (2014) | Terrestrial Species | Terrestrial Species |
| MCK34.1 | bear | 3.43 | -20.61 | Booth (2014) | Terrestrial Species | Terrestrial Species |
| MCK38.1 | bear | 5.08 | -19.03 | Booth (2014) | Terrestrial Species | Terrestrial Species |
| MCK42.1 | bear | 3.77 | -20.58 | Booth (2014) | Terrestrial Species | Terrestrial Species |
| MCK43.1 | bear | 3.96 | -20.23 | Booth (2014) | Terrestrial Species | Terrestrial Species |
| MCK44.1 | bear | 4.74 | -19.85 | Booth (2014) | Terrestrial Species | Terrestrial Species |
| MCK45.1 | bear | 4.5 | -20.71 | Booth (2014) | Terrestrial Species | Terrestrial Species |
| CAR02.1 | bear | 5.93 | -20.3 | Booth (2014) | Terrestrial Species | Terrestrial Species |
| CAR19.1 | bear | 5.77 | -21.18 | Booth (2014) | Terrestrial Species | Terrestrial Species |
| CAR33.1 | bear | 4.61 | -22.14 | Booth (2014) | Terrestrial Species | Terrestrial Species |
| CAR35.1 | bear | 4.63 | -20.98 | Booth (2014) | Terrestrial Species | Terrestrial Species |
| CAR36.1 | bear | 5.28 | -18.54 | Booth (2014) | Terrestrial Species | Terrestrial Species |
| IWP08.1 | bear | 4.35 | -21.12 | Booth (2014) | Terrestrial Species | Terrestrial Species |
| Bog-033 | bear | 5.97 | -20 | Booth (2014) | Terrestrial Species | Terrestrial Species |
| Bog-043 | bear | 4.75 | -20.46 | Booth (2014) | Terrestrial Species | Terrestrial Species |
| Fon-067 | bear | 4.98 | -20.89 | Booth (2014) | Terrestrial Species | Terrestrial Species |
| Fon-072 | bear | 5.78 | -22.32 | Booth (2014) | Terrestrial Species | Terrestrial Species |
| Ham-024 | bear | 4.9 | -19.92 | Booth (2014) | Terrestrial Species | Terrestrial Species |
| IWP(01)-052 | bear | 5.71 | -22.2 | Booth (2014) | Terrestrial Species | Terrestrial Species |
| IWP(09)-058 | bear | 4.35 | -21.12 | Booth (2014) | Terrestrial Species | Terrestrial Species |
| Lig-012 | bear | 4.47 | -19.98 | Booth (2014) | Terrestrial Species | Terrestrial Species |
| Pri-018 | bear | 4.92 | -20.86 | Booth (2014) | Terrestrial Species | Terrestrial Species |
| Sil-018 | bear | 6.24 | -21.1 | Booth (2014) | Terrestrial Species | Terrestrial Species |
| Sil-020 | bear | 6.21 | -20.71 | Booth (2014) | Terrestrial Species | Terrestrial Species |
| Van-039 | bear | 5.62 | -20.15 | Booth (2014) | Terrestrial Species | Terrestrial Species |
| Van-067 | bear | 6.59 | -22.9 | Booth (2014) | Terrestrial Species | Terrestrial Species |
| Van-071 | bear | 6.19 | -21.45 | Booth (2014) | Terrestrial Species | Terrestrial Species |
| Van-097 | bear | 5.98 | -21.79 | Booth (2014) | Terrestrial Species | Terrestrial Species |
| Van-114 | bear | 4.69 | -21.38 | Booth (2014) | Terrestrial Species | Terrestrial Species |
| Wal-045 | bear | 4.76 | -20.23 | Booth (2014) | Terrestrial Species | Terrestrial Species |
| Wal-046 | bear | 5.87 | -21.85 | Booth (2014) | Terrestrial Species | Terrestrial Species |
| Wal-047 | bear | 5.89 | -20.48 | Booth (2014) | Terrestrial Species | Terrestrial Species |
| Bog-038 | beaver | 4.79 | -21.37 | Booth (2014) | Terrestrial Species | Terrestrial Species |
| Wal-040 | beaver | 1.4 | -19.48 | Booth (2014) | Terrestrial Species | Terrestrial Species |
| Wal-041 | beaver | 6.12 | -22.34 | Booth (2014) | Terrestrial Species | Terrestrial Species |
| DOR16.1 | deer | 5.44 | -23.52 | Booth (2014) | Terrestrial Species | Terrestrial Species |
| DOR17.1 | deer | 6.06 | -23 | Booth (2014) | Terrestrial Species | Terrestrial Species |
| DOR18.1 | deer | 4.9 | -24.76 | Booth (2014) | Terrestrial Species | Terrestrial Species |
| DOR19.1 | deer | 5.86 | -25.31 | Booth (2014) | Terrestrial Species | Terrestrial Species |
| HOL03.1 | deer | 6.12 | -23.13 | Booth (2014) | Terrestrial Species | Terrestrial Species |
| HOL15.1 | deer | 4.62 | -19.4 | Booth (2014) | Terrestrial Species | Terrestrial Species |
| HOL23.1 | deer | 6.35 | -22.03 | Booth (2014) | Terrestrial Species | Terrestrial Species |
| HOL24.1 | deer | 5.46 | -22.31 | Booth (2014) | Terrestrial Species | Terrestrial Species |
| MCK03.1 | deer | 6.63 | -22.81 | Booth (2014) | Terrestrial Species | Terrestrial Species |
| MCK04.1 | deer | 4.83 | -22.01 | Booth (2014) | Terrestrial Species | Terrestrial Species |
| MCK12.1 | deer | 5.08 | -22.53 | Booth (2014) | Terrestrial Species | Terrestrial Species |
| MCK13.1 | deer | 4.82 | -21.78 | Booth (2014) | Terrestrial Species | Terrestrial Species |
| MCK17.1 | deer | 4.77 | -21.95 | Booth (2014) | Terrestrial Species | Terrestrial Species |
| MCK19.1 | deer | 6.21 | -21.31 | Booth (2014) | Terrestrial Species | Terrestrial Species |
| MCK20.1 | deer | 3.92 | -22.19 | Booth (2014) | Terrestrial Species | Terrestrial Species |
| MCK21.1 | deer | 3.74 | -22.77 | Booth (2014) | Terrestrial Species | Terrestrial Species |
| MCK22.1 | deer | 4.3 | -24.28 | Booth (2014) | Terrestrial Species | Terrestrial Species |
| MCK23.1 | deer | 5.11 | -22.43 | Booth (2014) | Terrestrial Species | Terrestrial Species |
| MCK33.1 | deer | 5.75 | -23.22 | Booth (2014) | Terrestrial Species | Terrestrial Species |
| MCK46.1 | deer | 6.49 | -21.91 | Booth (2014) | Terrestrial Species | Terrestrial Species |
| BrB-010 | deer | 4.75 | -23.46 | Morris (2015) | Terrestrial Species | Terrestrial Species |
| BrB-011 | deer | 4.88 | -23.63 | Morris (2015) | Terrestrial Species | Terrestrial Species |
| BrB-012 | deer | 4.35 | -21.6 | Morris (2015) | Terrestrial Species | Terrestrial Species |
| BrB-013 | deer | 4.99 | -23.6 | Morris (2015) | Terrestrial Species | Terrestrial Species |
| Bog-054 | deer | 5.03 | -23.02 | Morris (2015) | Terrestrial Species | Terrestrial Species |
| Clv-015 | deer | 6.12 | -22.05 | Morris (2015) | Terrestrial Species | Terrestrial Species |
| Clv-016 | deer | 5.68 | -22.38 | Morris (2015) | Terrestrial Species | Terrestrial Species |
| Clv-017 | deer | 8.16 | -21.19 | Morris (2015) | Terrestrial Species | Terrestrial Species |
| Clv-019 | deer | 5.13 | -22.65 | Morris (2015) | Terrestrial Species | Terrestrial Species |
| Cra-001 | deer | 4.58 | -23.33 | Morris (2015) | Terrestrial Species | Terrestrial Species |
| Crf-002 | deer | 4.15 | -23.45 | Morris (2015) | Terrestrial Species | Terrestrial Species |
| Crf-095 | deer | 5.17 | -21.85 | Morris (2015) | Terrestrial Species | Terrestrial Species |
| Dav-001 | deer | 3.9 | -23.92 | Morris (2015) | Terrestrial Species | Terrestrial Species |
| Dav-003 | deer | 4.27 | -23.16 | Morris (2015) | Terrestrial Species | Terrestrial Species |
| Dav-004 | deer | 6.16 | -22.01 | Morris (2015) | Terrestrial Species | Terrestrial Species |
| Fon-001 | deer | 4.98 | -22.8 | Morris (2015) | Terrestrial Species | Terrestrial Species |
| Fon-009 | deer | 6.14 | -22.79 | Morris (2015) | Terrestrial Species | Terrestrial Species |
| Fon-014 | deer | 5.81 | -22.19 | Morris (2015) | Terrestrial Species | Terrestrial Species |
| Fon-019 | deer | 5.46 | -22.79 | Morris (2015) | Terrestrial Species | Terrestrial Species |
| Fon-019 mDUP | deer | 5.61 | -22.64 | Morris (2015) | Terrestrial Species | Terrestrial Species |
| Fon-047 | deer | 5.12 | -22.91 | Morris (2015) | Terrestrial Species | Terrestrial Species |
| Fon-047 DUP | deer | 2.83 | -24.88 | Morris (2015) | Terrestrial Species | Terrestrial Species |
| Ham-004 | deer | 4.98 | -22.02 | Morris (2015) | Terrestrial Species | Terrestrial Species |
| IWP(01)-001 | deer | 5.29 | -23.34 | Morris (2015) | Terrestrial Species | Terrestrial Species |
| IWP(01)-001 mDUP | deer | 5.31 | -23.38 | Morris (2015) | Terrestrial Species | Terrestrial Species |
| IWP(01)-009 | deer | 5.68 | -23.36 | Morris (2015) | Terrestrial Species | Terrestrial Species |
| IWP(01)-025 | deer | 5.65 | -24.07 | Morris (2015) | Terrestrial Species | Terrestrial Species |
| IWP(01)-025 DUP | deer | 5.34 | -23.85 | Morris (2015) | Terrestrial Species | Terrestrial Species |
| IWP(01)-036 DUP | deer | 5.47 | -23.64 | Morris (2015) | Terrestrial Species | Terrestrial Species |
| IWP(01)-036 mDUP | deer | 4.96 | -23.68 | Morris (2015) | Terrestrial Species | Terrestrial Species |
| IWP(03)-23 | deer | 4.85 | -23.49 | Morris (2015) | Terrestrial Species | Terrestrial Species |
| IWP(09)-002 | deer | 4.92 | -23.84 | Morris (2015) | Terrestrial Species | Terrestrial Species |
| IWP(09)-047 | deer | 4.78 | -22.95 | Morris (2015) | Terrestrial Species | Terrestrial Species |
| IWP(09)-047 DUP | deer | 4.8 | -23.32 | Morris (2015) | Terrestrial Species | Terrestrial Species |
| IWP(09)-047 DUP | deer | 4.91 | -22.79 | Morris (2015) | Terrestrial Species | Terrestrial Species |
| IWP(09)-054 | deer | 5.07 | -23.31 | Morris (2015) | Terrestrial Species | Terrestrial Species |
| IWP(09)-054 mDUP | deer | 5.25 | -23.54 | Morris (2015) | Terrestrial Species | Terrestrial Species |
| IWP(09)-134 | deer | 5.19 | -23.43 | Morris (2015) | Terrestrial Species | Terrestrial Species |
| IWP(09)-134 mDUP | deer | 5.13 | -23.4 | Morris (2015) | Terrestrial Species | Terrestrial Species |
| IWP(12)-003 | deer | 4.7 | -22.54 | Morris (2015) | Terrestrial Species | Terrestrial Species |
| IWP(12)-004 | deer | 5.35 | -22.14 | Morris (2015) | Terrestrial Species | Terrestrial Species |
| IWP(12)-005 | deer | 5.14 | -22.94 | Morris (2015) | Terrestrial Species | Terrestrial Species |
| Lia-006 | deer | 6.82 | -23.82 | Morris (2015) | Terrestrial Species | Terrestrial Species |
| Lia-010 | deer | 8.62 | -20.72 | Morris (2015) | Terrestrial Species | Terrestrial Species |
| Mon-004 | deer | 5.96 | -22.48 | Morris (2015) | Terrestrial Species | Terrestrial Species |
| Mon-005 | deer | 4.4 | -21.79 | Morris (2015) | Terrestrial Species | Terrestrial Species |
| Mon-006 | deer | 4.78 | -22.66 | Morris (2015) | Terrestrial Species | Terrestrial Species |
| Mon-007 | deer | 5.33 | -23.05 | Morris (2015) | Terrestrial Species | Terrestrial Species |
| Mon-008 | deer | 5.41 | -23.1 | Morris (2015) | Terrestrial Species | Terrestrial Species |
| OLG-001 | deer | 7 | -22.12 | Morris (2015) | Terrestrial Species | Terrestrial Species |
| OLG-002 | deer | 6.07 | -21.7 | Morris (2015) | Terrestrial Species | Terrestrial Species |
| OLG-013 | deer | 5.08 | -23.22 | Morris (2015) | Terrestrial Species | Terrestrial Species |
| Pip(1)-103 | deer | 3.73 | -22.06 | Morris (2015) | Terrestrial Species | Terrestrial Species |
| Pip(1)-157 | deer | 4.54 | -22.43 | Morris (2015) | Terrestrial Species | Terrestrial Species |
| Por-009 | deer | 6.12 | -22.09 | Morris (2015) | Terrestrial Species | Terrestrial Species |
| Por-017 | deer | 5.01 | -22.13 | Morris (2015) | Terrestrial Species | Terrestrial Species |
| Por-017 mDUP | deer | 5.04 | -21.99 | Morris (2015) | Terrestrial Species | Terrestrial Species |
| Pri-008 | deer | 4.95 | -23.05 | Morris (2015) | Terrestrial Species | Terrestrial Species |
| Pri-017 | deer | 4.91 | -22.35 | Morris (2015) | Terrestrial Species | Terrestrial Species |
| Pri-017 DUP | deer | 5.85 | -20.24 | Morris (2015) | Terrestrial Species | Terrestrial Species |
| Pri-019 | deer | 4.41 | -22.54 | Morris (2015) | Terrestrial Species | Terrestrial Species |
| Pri-019 DUP | deer | 2.99 | -22.46 | Morris (2015) | Terrestrial Species | Terrestrial Species |
| Rif-007 | deer | 8.23 | -21.25 | Morris (2015) | Terrestrial Species | Terrestrial Species |
| Rif-007 mDUP | deer | 8.05 | -21.32 | Morris (2015) | Terrestrial Species | Terrestrial Species |
| Rif-077 | deer | 6.8 | -22.65 | Morris (2015) | Terrestrial Species | Terrestrial Species |
| Sil-019 | deer | 5.54 | -22.65 | Morris (2015) | Terrestrial Species | Terrestrial Species |
| Sil-019 DUP | deer | 5.48 | -23.25 | Morris (2015) | Terrestrial Species | Terrestrial Species |
| Sil-026 | deer | 6.09 | -23.81 | Morris (2015) | Terrestrial Species | Terrestrial Species |
| Sil-026 DUP | deer | 6.17 | -23.95 | Morris (2015) | Terrestrial Species | Terrestrial Species |
| Sla-017 | deer | 5.82 | -24.18 | Morris (2015) | Terrestrial Species | Terrestrial Species |
| Tho-002 | deer | 5.73 | -22.07 | Morris (2015) | Terrestrial Species | Terrestrial Species |
| Tho-012 | deer | 5.75 | -22.08 | Morris (2015) | Terrestrial Species | Terrestrial Species |
| Tho-012 mDUP | deer | 5.87 | -22 | Morris (2015) | Terrestrial Species | Terrestrial Species |
| Tho-018 | deer | 5.69 | -22.07 | Morris (2015) | Terrestrial Species | Terrestrial Species |
| Van-001 | deer | 5.52 | -23 | Morris (2015) | Terrestrial Species | Terrestrial Species |
| Van-001 mDUP | deer | 5.82 | -23.03 | Morris (2015) | Terrestrial Species | Terrestrial Species |
| Van-003 | deer | 4.49 | -21.82 | Morris (2015) | Terrestrial Species | Terrestrial Species |
| Van003 mDUP | deer | 4.47 | -21.82 | Morris (2015) | Terrestrial Species | Terrestrial Species |
| Van-018 | deer | 6.33 | -21.45 | Morris (2015) | Terrestrial Species | Terrestrial Species |
| Van-019 | deer | 5.56 | -22.2 | Morris (2015) | Terrestrial Species | Terrestrial Species |
| Van-020 | deer | 5.35 | -23.84 | Morris (2015) | Terrestrial Species | Terrestrial Species |
| Van-022 | deer | 4.92 | -24.06 | Morris (2015) | Terrestrial Species | Terrestrial Species |
| Van-108 | deer | 5.45 | -23.1 | Morris (2015) | Terrestrial Species | Terrestrial Species |
| Wal-003 | deer | 4.18 | -23.79 | Morris (2015) | Terrestrial Species | Terrestrial Species |
| Wal-005 | deer | 4.29 | -23.29 | Morris (2015) | Terrestrial Species | Terrestrial Species |
| Wal-008 | deer | 6.11 | -23.71 | Morris (2015) | Terrestrial Species | Terrestrial Species |
| Wal-009 | deer | 5.86 | -24.32 | Morris (2015) | Terrestrial Species | Terrestrial Species |
| Wal-010 | deer | 5.54 | -23.65 | Morris (2015) | Terrestrial Species | Terrestrial Species |
| Wal-011 | deer | 4.77 | -22.56 | Morris (2015) | Terrestrial Species | Terrestrial Species |
| Wal-013 | deer | 4.49 | -23.54 | Morris (2015) | Terrestrial Species | Terrestrial Species |
| Wal-014 | deer | 5.61 | -24.06 | Morris (2015) | Terrestrial Species | Terrestrial Species |
| Wal-014 | deer | 4.26 | -23.56 | Morris (2015) | Terrestrial Species | Terrestrial Species |
| Wal-016 | deer | 4.26 | -23.84 | Morris (2015) | Terrestrial Species | Terrestrial Species |
| Wal-018 | deer | 5.08 | -22.37 | Morris (2015) | Terrestrial Species | Terrestrial Species |
| Wal-021 | deer | 5.35 | -24.66 | Morris (2015) | Terrestrial Species | Terrestrial Species |
| Wal-036 | deer | 6.13 | -22.4 | Morris (2015) | Terrestrial Species | Terrestrial Species |
| Wal-037 | deer | 4.76 | -23.36 | Morris (2015) | Terrestrial Species | Terrestrial Species |
| Wal-038 | deer | 5.54 | -21.91 | Morris (2015) | Terrestrial Species | Terrestrial Species |
| Win-157 | deer | 8.17 | -21.19 | Morris (2015) | Terrestrial Species | Terrestrial Species |
| Win-159 | deer | 7.29 | -21.93 | Morris (2015) | Terrestrial Species | Terrestrial Species |
| BrB-02 | deer | 5.5 | -20.89 | Morris (2015) | Terrestrial Species | Terrestrial Species |
| BrB-03 | deer | 5.28 | -20.68 | Morris (2015) | Terrestrial Species | Terrestrial Species |
| Clv-033 + | deer | 6.25 | -20.77 | Morris (2015) | Terrestrial Species | Terrestrial Species |
| Crf-043~ | deer | 6.04 | -20.61 | Morris (2015) | Terrestrial Species | Terrestrial Species |
| Crf-044~ | deer | 5.77 | -20.16 | Morris (2015) | Terrestrial Species | Terrestrial Species |
| Crf-045~ | deer | 6.74 | -17.75 | Morris (2015) | Terrestrial Species | Terrestrial Species |
| Crf-046~ | deer | 6.6 | -18.53 | Morris (2015) | Terrestrial Species | Terrestrial Species |
| Crf-047~ | deer | 7.24 | -17.44 | Morris (2015) | Terrestrial Species | Terrestrial Species |
| Crf-048~ | deer | 6.56 | -18.78 | Morris (2015) | Terrestrial Species | Terrestrial Species |
| Crf-051~ | deer | 6.17 | -20.92 | Morris (2015) | Terrestrial Species | Terrestrial Species |
| Fon-020 | deer | 6.31 | -21.59 | Morris (2015) | Terrestrial Species | Terrestrial Species |
| Fon-033 | deer | 6.9 | -21.01 | Morris (2015) | Terrestrial Species | Terrestrial Species |
| Fon-104 | deer | 5.39 | -21.29 | Morris (2015) | Terrestrial Species | Terrestrial Species |
| Ham-05 | deer | 8.17 | -9.93 | Morris (2015) | Terrestrial Species | Terrestrial Species |
| Ham-06 | deer | 6.25 | -19.8 | Morris (2015) | Terrestrial Species | Terrestrial Species |
| Ham-07 | deer | 6.13 | -20.64 | Morris (2015) | Terrestrial Species | Terrestrial Species |
| Ham-08 | deer | 6.75 | -17.08 | Morris (2015) | Terrestrial Species | Terrestrial Species |
| Ham-09~ | deer | 6.39 | -19.59 | Morris (2015) | Terrestrial Species | Terrestrial Species |
| Ham-10~ | deer | 4.88 | -22.83 | Morris (2015) | Terrestrial Species | Terrestrial Species |
| Ham-11~ | deer | 5.55 | -19.13 | Morris (2015) | Terrestrial Species | Terrestrial Species |
| IWP(01)-30 | deer | 6.75 | -22.37 | Morris (2015) | Terrestrial Species | Terrestrial Species |
| IWP(01)-30 DUP | deer | 6.76 | -22.31 | Morris (2015) | Terrestrial Species | Terrestrial Species |
| IWP(03)-02 | deer | 6.76 | -21.88 | Morris (2015) | Terrestrial Species | Terrestrial Species |
| IWP(03)-06 | deer | 6.58 | -21.45 | Morris (2015) | Terrestrial Species | Terrestrial Species |
| IWP(03)-07 | deer | 5.79 | -23.48 | Morris (2015) | Terrestrial Species | Terrestrial Species |
| IWP(03)-08 | deer | 5.47 | -23.18 | Morris (2015) | Terrestrial Species | Terrestrial Species |
| IWP(03)-15 | deer | 6.14 | -21.85 | Morris (2015) | Terrestrial Species | Terrestrial Species |
| IWP(09)-009 | deer | 5.67 | -21.49 | Morris (2015) | Terrestrial Species | Terrestrial Species |
| IWP(09)-012 | deer | 5.75 | -23.56 | Morris (2015) | Terrestrial Species | Terrestrial Species |
| IWP(09)-012 DUP | deer | 6.6 | -23.28 | Morris (2015) | Terrestrial Species | Terrestrial Species |
| IWP(09)-032 | deer | 6.78 | -22.07 | Morris (2015) | Terrestrial Species | Terrestrial Species |
| IWP(09)-048 | deer | 4.72 | -20.21 | Morris (2015) | Terrestrial Species | Terrestrial Species |
| IWP(09)-079 | deer | 6.67 | -23.24 | Morris (2015) | Terrestrial Species | Terrestrial Species |
| IWP(09)-083 | deer | 5.96 | -23.67 | Morris (2015) | Terrestrial Species | Terrestrial Species |
| IWP(09)-083 mDUP | deer | 6.29 | -23.75 | Morris (2015) | Terrestrial Species | Terrestrial Species |
| IWP(09)-088 | deer | 8.49 | -22.45 | Morris (2015) | Terrestrial Species | Terrestrial Species |
| IWP(09)-119 | deer | 5.71 | -23.17 | Morris (2015) | Terrestrial Species | Terrestrial Species |
| IWP(09)-122 | deer | 7.64 | -21.3 | Morris (2015) | Terrestrial Species | Terrestrial Species |
| Pip(1)-010^ | deer | 6.18 | -20.52 | Morris (2015) | Terrestrial Species | Terrestrial Species |
| Pip(1)-023 + | deer | 6.39 | -20.76 | Morris (2015) | Terrestrial Species | Terrestrial Species |
| Pip(1)-024 + | deer | 6.03 | -21.05 | Morris (2015) | Terrestrial Species | Terrestrial Species |
| Pip(1)-024 mDUP | deer | 6.15 | -20.83 | Morris (2015) | Terrestrial Species | Terrestrial Species |
| Pip(1)-024 mDUP DUP | deer | 5.21 | -20.77 | Morris (2015) | Terrestrial Species | Terrestrial Species |
| Pip(1)-025 | deer | 6.03 | -21.52 | Morris (2015) | Terrestrial Species | Terrestrial Species |
| Pip(1)-048 | deer | 6.84 | -19.88 | Morris (2015) | Terrestrial Species | Terrestrial Species |
| Pip(1)-075 | deer | 8.49 | -20.62 | Morris (2015) | Terrestrial Species | Terrestrial Species |
| Pip(1)-179 | deer | 5.97 | -20.4 | Morris (2015) | Terrestrial Species | Terrestrial Species |
| Pip(1)-184 | deer | 5.44 | -22.5 | Morris (2015) | Terrestrial Species | Terrestrial Species |
| Pip(2)-070 | deer | 6.86 | -20.07 | Morris (2015) | Terrestrial Species | Terrestrial Species |
| Pri-007 | deer | 5.24 | -18.33 | Morris (2015) | Terrestrial Species | Terrestrial Species |
| Rif-062 | deer | 7.12 | -20.63 | Morris (2015) | Terrestrial Species | Terrestrial Species |
| Rif-080 | deer | 6.81 | -22.34 | Morris (2015) | Terrestrial Species | Terrestrial Species |
| Rif-092 | deer | 6.78 | -19.8 | Morris (2015) | Terrestrial Species | Terrestrial Species |
| Rif-092 mDUP | deer | 6.72 | -19.74 | Morris (2015) | Terrestrial Species | Terrestrial Species |
| Rif-107 | deer | 5.18 | -22.96 | Morris (2015) | Terrestrial Species | Terrestrial Species |
| Rif-107 DUP | deer | 5.05 | -23.07 | Morris (2015) | Terrestrial Species | Terrestrial Species |
| Tho-035 | deer | 5.03 | -22.36 | Morris (2015) | Terrestrial Species | Terrestrial Species |
| Tho-046 | deer | 5.86 | -22.31 | Morris (2015) | Terrestrial Species | Terrestrial Species |
| Tho-054 | deer | 4 | -21.78 | Morris (2015) | Terrestrial Species | Terrestrial Species |
| Tho-054 DUP | deer | 4.81 | -21.8 | Morris (2015) | Terrestrial Species | Terrestrial Species |
| Tho-058 | deer | 4.87 | -22.22 | Morris (2015) | Terrestrial Species | Terrestrial Species |
| Tho-065 | deer | 6.6 | -22.16 | Morris (2015) | Terrestrial Species | Terrestrial Species |
| Van-011 | deer | 5.56 | -21.33 | Morris (2015) | Terrestrial Species | Terrestrial Species |
| Van-012 | deer | 6.76 | -21.32 | Morris (2015) | Terrestrial Species | Terrestrial Species |
| Van-017 | deer | 6.31 | -20.93 | Morris (2015) | Terrestrial Species | Terrestrial Species |
| Wal-050 | deer | 5.46 | -20.19 | Morris (2015) | Terrestrial Species | Terrestrial Species |
| Wal-050 mDUP | deer | 5.61 | -20.2 | Morris (2015) | Terrestrial Species | Terrestrial Species |
| Wal-051 | deer | 5.63 | -21.95 | Morris (2015) | Terrestrial Species | Terrestrial Species |
| Win-047 | deer | 6.92 | -19.55 | Morris (2015) | Terrestrial Species | Terrestrial Species |
| Win-047 DUP | deer | 6.85 | -19.58 | Morris (2015) | Terrestrial Species | Terrestrial Species |
| Win-221 | deer | 6.11 | -19.02 | Morris (2015) | Terrestrial Species | Terrestrial Species |
| Crf-077 DUP | fox | 10.4 | -19.62 | Morris (2015) | Terrestrial Species | Terrestrial Species |
| Crf-077 | fox | 10.3 | -19.67 | Morris (2015) | Terrestrial Species | Terrestrial Species |
| Pip(2)-016 | fox | 8.51 | -18.59 | Morris (2015) | Terrestrial Species | Terrestrial Species |
| Tho-011 | fox | 7.57 | -17.95 | Morris (2015) | Terrestrial Species | Terrestrial Species |
| Van-070 | fox | 8.9 | -19.53 | Morris (2015) | Terrestrial Species | Terrestrial Species |
| Win-154 | fox | 9.08 | -18.37 | Morris (2015) | Terrestrial Species | Terrestrial Species |
| Cra-015 | groundhog | 4.25 | -23.3 | Morris (2015) | Terrestrial Species | Terrestrial Species |
| Fon-025 | groundhog | 2.53 | -24.22 | Morris (2015) | Terrestrial Species | Terrestrial Species |
| Fon-049 | groundhog | 3.94 | -19.4 | Morris (2015) | Terrestrial Species | Terrestrial Species |
| Lig-004 | groundhog | 2.27 | -23.21 | Morris (2015) | Terrestrial Species | Terrestrial Species |
| Lig-009 | groundhog | 2.79 | -23.05 | Morris (2015) | Terrestrial Species | Terrestrial Species |
| Lig-014 | groundhog | 2.67 | -23.3 | Morris (2015) | Terrestrial Species | Terrestrial Species |
| Tho-007 | groundhog | 2.27 | -23.76 | Morris (2015) | Terrestrial Species | Terrestrial Species |
| Van-044 | groundhog | 3.09 | -25.6 | Morris (2015) | Terrestrial Species | Terrestrial Species |
| Van-056 | groundhog | 3.11 | -23.66 | Morris (2015) | Terrestrial Species | Terrestrial Species |
| Van-069 | groundhog | 3.05 | -25.49 | Morris (2015) | Terrestrial Species | Terrestrial Species |
| Van-072 | groundhog | 3.13 | -26.39 | Morris (2015) | Terrestrial Species | Terrestrial Species |
| Van-080 | groundhog | 2.73 | -25.75 | Morris (2015) | Terrestrial Species | Terrestrial Species |
| Van-093 | groundhog | 3.2 | -25.67 | Morris (2015) | Terrestrial Species | Terrestrial Species |
| Van-095 | groundhog | 2.95 | -26.45 | Morris (2015) | Terrestrial Species | Terrestrial Species |
| Van-113 | groundhog | 4.86 | -22.89 | Morris (2015) | Terrestrial Species | Terrestrial Species |
| Van-119 | groundhog | 3.01 | -25.76 | Morris (2015) | Terrestrial Species | Terrestrial Species |
| Wal-017 | groundhog | 2.13 | -23.17 | Morris (2015) | Terrestrial Species | Terrestrial Species |
| Wal-020 | groundhog | 3.34 | -25.05 | Morris (2015) | Terrestrial Species | Terrestrial Species |
| Lia-014 | muskrat | 4.71 | -20.43 | Morris (2015) | Terrestrial Species | Terrestrial Species |
| Wal-025 | muskrat | 7.28 | -23 | Morris (2015) | Terrestrial Species | Terrestrial Species |
| Wal-052 | muskrat | 6.79 | -20.55 | Morris (2015) | Terrestrial Species | Terrestrial Species |
| Lia-007 | porcupine | 4.93 | -19.86 | Morris (2015) | Terrestrial Species | Terrestrial Species |
| Van-102 | porcupine | 5.6 | -21.41 | Morris (2015) | Terrestrial Species | Terrestrial Species |
| Wal-054 | porcupine | 4.42 | -20.21 | Morris (2015) | Terrestrial Species | Terrestrial Species |
| OLG-015 | rabbit | 4.66 | -19.74 | Morris (2015) | Terrestrial Species | Terrestrial Species |
| Pip(2)-017 | rabbit | 4.11 | -19.49 | Morris (2015) | Terrestrial Species | Terrestrial Species |
| Tho-019 | rabbit | 3.44 | -22.12 | Morris (2015) | Terrestrial Species | Terrestrial Species |
| Tho-023 | rabbit | 3.46 | -22.08 | Morris (2015) | Terrestrial Species | Terrestrial Species |
| Van-068 | rabbit | 3.96 | -27.35 | Morris (2015) | Terrestrial Species | Terrestrial Species |
| Van-118 | rabbit | 4.14 | -23.1 | Morris (2015) | Terrestrial Species | Terrestrial Species |
| Wal-024 | rabbit | 4.08 | -22.05 | Morris (2015) | Terrestrial Species | Terrestrial Species |
| Wal-053 | rabbit | 2.09 | -27.08 | Morris (2015) | Terrestrial Species | Terrestrial Species |
| PRY01.1 | raccoon | 9.36 | -21.34 | Booth (2014) | Terrestrial Species | Terrestrial Species |
| PRY02.1 | raccoon | 10.37 | -20.91 | Booth (2014) | Terrestrial Species | Terrestrial Species |
| PRY03.1 | raccoon | 8.87 | -21.08 | Booth (2014) | Terrestrial Species | Terrestrial Species |
| PRY07.1 | raccoon | 9.35 | -21.82 | Booth (2014) | Terrestrial Species | Terrestrial Species |
| PRY08.1 | raccoon | 9.65 | -23.13 | Booth (2014) | Terrestrial Species | Terrestrial Species |
| PRY09.1 | raccoon | 9.45 | -21.16 | Booth (2014) | Terrestrial Species | Terrestrial Species |
| PRY11.1 | raccoon | 8.8 | -20.56 | Booth (2014) | Terrestrial Species | Terrestrial Species |
| PRY12.1 | raccoon | 9.16 | -20.39 | Booth (2014) | Terrestrial Species | Terrestrial Species |
| PRY13.1 | raccoon | 8.72 | -20.45 | Booth (2014) | Terrestrial Species | Terrestrial Species |
| PRY15.1 | raccoon | 9.18 | -20.69 | Booth (2014) | Terrestrial Species | Terrestrial Species |
| PRY16.1 | raccoon | 9.83 | -21.45 | Booth (2014) | Terrestrial Species | Terrestrial Species |
| PRY17.1 | raccoon | 6.18 | -20.93 | Booth (2014) | Terrestrial Species | Terrestrial Species |
| PRY18.1 | raccoon | 8.81 | -21.23 | Morris (2015) | Terrestrial Species | Terrestrial Species |
| Bog-002 | raccoon | 8.09 | -20.96 | Morris (2015) | Terrestrial Species | Terrestrial Species |
| Crf-039 | raccoon | 6.76 | -13.98 | Morris (2015) | Terrestrial Species | Terrestrial Species |
| Crf-040 | raccoon | 7.51 | -15.52 | Morris (2015) | Terrestrial Species | Terrestrial Species |
| Fon-109 | raccoon | 8.95 | -20.96 | Morris (2015) | Terrestrial Species | Terrestrial Species |
| IWP(01)-017 | raccoon | 9.47 | -20.36 | Morris (2015) | Terrestrial Species | Terrestrial Species |
| IWP(09)-001 | raccoon | 7.84 | -19.88 | Morris (2015) | Terrestrial Species | Terrestrial Species |
| IWP(09)-004 | raccoon | 9.65 | -21.28 | Morris (2015) | Terrestrial Species | Terrestrial Species |
| IWP(09)-010 | raccoon | 9.72 | -20.66 | Morris (2015) | Terrestrial Species | Terrestrial Species |
| IWP(09)-014 | raccoon | 9.03 | -20.07 | Morris (2015) | Terrestrial Species | Terrestrial Species |
| IWP(09)-018 | raccoon | 9.45 | -21.56 | Morris (2015) | Terrestrial Species | Terrestrial Species |
| IWP(09)-040 | raccoon | 10.35 | -21.27 | Morris (2015) | Terrestrial Species | Terrestrial Species |
| IWP(09)-078 | raccoon | 7.68 | -20.13 | Morris (2015) | Terrestrial Species | Terrestrial Species |
| IWP(09)-111 | raccoon | 9.13 | -20.82 | Morris (2015) | Terrestrial Species | Terrestrial Species |
| IWP(09)-116 | raccoon | 5.83 | -20.77 | Morris (2015) | Terrestrial Species | Terrestrial Species |
| IWP(09)-118 | raccoon | 8.49 | -20.93 | Morris (2015) | Terrestrial Species | Terrestrial Species |
| IWP(09)-131 | raccoon | 9.64 | -20.48 | Morris (2015) | Terrestrial Species | Terrestrial Species |
| Lia-001 | raccoon | 9.84 | -23.48 | Morris (2015) | Terrestrial Species | Terrestrial Species |
| Lia-012 | raccoon | 4.62 | -21.36 | Morris (2015) | Terrestrial Species | Terrestrial Species |
| Lia-013 | raccoon | 9.11 | -23.61 | Morris (2015) | Terrestrial Species | Terrestrial Species |
| Lia-013 DUP | raccoon | 9.09 | -23.72 | Morris (2015) | Terrestrial Species | Terrestrial Species |
| Mon-001 | raccoon | 9.25 | -21.29 | Morris (2015) | Terrestrial Species | Terrestrial Species |
| Pip(1)-151 | raccoon | 9.58 | -22.87 | Morris (2015) | Terrestrial Species | Terrestrial Species |
| Sil-006 | raccoon | 9.08 | -20.46 | Morris (2015) | Terrestrial Species | Terrestrial Species |
| Van-103 | raccoon | 9.59 | -20.5 | Morris (2015) | Terrestrial Species | Terrestrial Species |
| Van-106 | raccoon | 8.87 | -20.94 | Morris (2015) | Terrestrial Species | Terrestrial Species |
| Wal-042 | raccoon | 7.44 | -21.27 | Morris (2015) | Terrestrial Species | Terrestrial Species |
| Wal-055 | raccoon | 8.3 | -22.25 | Morris (2015) | Terrestrial Species | Terrestrial Species |
| Wal-056 | raccoon | 8.81 | -21.27 | Morris (2015) | Terrestrial Species | Terrestrial Species |
| Win-218 | raccoon | 7.46 | -19.73 | Morris (2015) | Terrestrial Species | Terrestrial Species |
| Win-218 DUP | raccoon | 7.44 | -19.73 | Morris (2015) | Terrestrial Species | Terrestrial Species |
| Win-233 | raccoon | 9.56 | -21.67 | Morris (2015) | Terrestrial Species | Terrestrial Species |
| Sla-007 | raccoon | 5.04 | -23.18 | Morris (2015) | Terrestrial Species | Terrestrial Species |
| Sla-034 | raccoon | 5.08 | -22.96 | Morris (2015) | Terrestrial Species | Terrestrial Species |
| Wal-026 | raccoon | 7.23 | -25.05 | Morris (2015) | Terrestrial Species | Terrestrial Species |
| Fon-030 | squirrel | 5.54 | -18.53 | Morris (2015) | Terrestrial Species | Terrestrial Species |
| Fon-064 | squirrel | 4.64 | -20.45 | Morris (2015) | Terrestrial Species | Terrestrial Species |
| Fon-091 | squirrel | 5.04 | -19.83 | Morris (2015) | Terrestrial Species | Terrestrial Species |
| Fon-113 | squirrel | 5.15 | -20.02 | Morris (2015) | Terrestrial Species | Terrestrial Species |
| Sla-032 | squirrel | 4.34 | -19.38 | Morris (2015) | Terrestrial Species | Terrestrial Species |
| Tho-005 | squirrel | 5.03 | -19.2 | Morris (2015) | Terrestrial Species | Terrestrial Species |
| Van-041 | squirrel | 4.52 | -20.42 | Morris (2015) | Terrestrial Species | Terrestrial Species |
| Van-042 | squirrel | 4.09 | -20.28 | Morris (2015) | Terrestrial Species | Terrestrial Species |
| Van-052 | squirrel | 6.66 | -18.5 | Morris (2015) | Terrestrial Species | Terrestrial Species |
| Van-085 | squirrel | 4.97 | -19.49 | Morris (2015) | Terrestrial Species | Terrestrial Species |
| Van-090 | squirrel | 6.39 | -19.76 | Morris (2015) | Terrestrial Species | Terrestrial Species |
| Van-091 | squirrel | 4.86 | -19.6 | Morris (2015) | Terrestrial Species | Terrestrial Species |
| Wal-048 | squirrel | 4.54 | -19.32 | Morris (2015) | Terrestrial Species | Terrestrial Species |
| Wal-049 | squirrel | 3.84 | -19.47 | Morris (2015) | Terrestrial Species | Terrestrial Species |
| BrB-02 | turkey | 5.5 | -20.89 | Morris et al (2016) | Terrestrial Species | Terrestrial Species |
| BrB-03 | turkey | 5.28 | -20.68 | Morris et al (2016) | Terrestrial Species | Terrestrial Species |
| Clv-033 + | turkey | 6.25 | -20.77 | Morris et al (2016) | Terrestrial Species | Terrestrial Species |
| Crf-043~ | turkey | 6.04 | -20.61 | Morris et al (2016) | Terrestrial Species | Terrestrial Species |
| Crf-044~ | turkey | 5.77 | -20.16 | Morris et al (2016) | Terrestrial Species | Terrestrial Species |
| Crf-045~ | turkey | 6.74 | -17.75 | Morris et al (2016) | Terrestrial Species | Terrestrial Species |
| Crf-046~ | turkey | 6.6 | -18.53 | Morris et al (2016) | Terrestrial Species | Terrestrial Species |
| Crf-047~ | turkey | 7.24 | -17.44 | Morris et al (2016) | Terrestrial Species | Terrestrial Species |
| Crf-048~ | turkey | 6.56 | -18.78 | Morris et al (2016) | Terrestrial Species | Terrestrial Species |
| Crf-051~ | turkey | 6.17 | -20.92 | Morris et al (2016) | Terrestrial Species | Terrestrial Species |
| Fon-020 | turkey | 6.31 | -21.59 | Morris et al (2016) | Terrestrial Species | Terrestrial Species |
| Fon-033 | turkey | 6.9 | -21.01 | Morris et al (2016) | Terrestrial Species | Terrestrial Species |
| Fon-104 | turkey | 5.39 | -21.29 | Morris et al (2016) | Terrestrial Species | Terrestrial Species |
| Ham-05 | turkey | 8.17 | -9.93 | Morris et al (2016) | Terrestrial Species | Terrestrial Species |
| Ham-06 | turkey | 6.25 | -19.8 | Morris et al (2016) | Terrestrial Species | Terrestrial Species |
| Ham-07 | turkey | 6.13 | -20.64 | Morris et al (2016) | Terrestrial Species | Terrestrial Species |
| Ham-08 | turkey | 6.75 | -17.08 | Morris et al (2016) | Terrestrial Species | Terrestrial Species |
| Ham-09~ | turkey | 6.39 | -19.59 | Morris et al (2016) | Terrestrial Species | Terrestrial Species |
| Ham-10~ | turkey | 4.88 | -22.83 | Morris et al (2016) | Terrestrial Species | Terrestrial Species |
| Ham-11~ | turkey | 5.55 | -19.13 | Morris et al (2016) | Terrestrial Species | Terrestrial Species |
| IWP(01)-30 | turkey | 6.75 | -22.37 | Morris et al (2016) | Terrestrial Species | Terrestrial Species |
| IWP(03)-02 | turkey | 6.76 | -21.88 | Morris et al (2016) | Terrestrial Species | Terrestrial Species |
| IWP(03)-06 | turkey | 6.58 | -21.45 | Morris et al (2016) | Terrestrial Species | Terrestrial Species |
| IWP(03)-07 | turkey | 5.79 | -23.48 | Morris et al (2016) | Terrestrial Species | Terrestrial Species |
| IWP(03)-08 | turkey | 5.47 | -23.18 | Morris et al (2016) | Terrestrial Species | Terrestrial Species |
| IWP(03)-15 | turkey | 6.14 | -21.85 | Morris et al (2016) | Terrestrial Species | Terrestrial Species |
| IWP(09)-009 | turkey | 5.67 | -21.49 | Morris et al (2016) | Terrestrial Species | Terrestrial Species |
| IWP(09)-012 | turkey | 5.75 | -23.56 | Morris et al (2016) | Terrestrial Species | Terrestrial Species |
| IWP(09)-048 | turkey | 4.72 | -20.21 | Morris et al (2016) | Terrestrial Species | Terrestrial Species |
| IWP(09)-079 | turkey | 6.67 | -23.24 | Morris et al (2016) | Terrestrial Species | Terrestrial Species |
| IWP(09)-083 | turkey | 5.96 | -23.67 | Morris et al (2016) | Terrestrial Species | Terrestrial Species |
| IWP(09)-032 | turkey | 6.78 | -22.07 | Morris et al (2016) | Terrestrial Species | Terrestrial Species |
| IWP(09)-088 | turkey | 8.49 | -22.45 | Morris et al (2016) | Terrestrial Species | Terrestrial Species |
| IWP(09)-119 | turkey | 5.71 | -23.17 | Morris et al (2016) | Terrestrial Species | Terrestrial Species |
| IWP(09)-122 | turkey | 7.64 | -21.3 | Morris et al (2016) | Terrestrial Species | Terrestrial Species |
| Pip(1)-010^ | turkey | 6.18 | -20.52 | Morris et al (2016) | Terrestrial Species | Terrestrial Species |
| Pip(1)-023 + | turkey | 6.39 | -20.76 | Morris et al (2016) | Terrestrial Species | Terrestrial Species |
| Pip(1)-024 + | turkey | 6.03 | -21.05 | Morris et al (2016) | Terrestrial Species | Terrestrial Species |
| Pip(1)-025 | turkey | 6.03 | -21.52 | Morris et al (2016) | Terrestrial Species | Terrestrial Species |
| Pip(1)-048 | turkey | 6.84 | -19.88 | Morris et al (2016) | Terrestrial Species | Terrestrial Species |
| Pip(1)-075 | turkey | 8.49 | -20.62 | Morris et al (2016) | Terrestrial Species | Terrestrial Species |
| Pip(1)-179 | turkey | 5.97 | -20.4 | Morris et al (2016) | Terrestrial Species | Terrestrial Species |
| Pip(1)-184 | turkey | 5.44 | -22.5 | Morris et al (2016) | Terrestrial Species | Terrestrial Species |
| Pip(2)-070 | turkey | 6.86 | -20.07 | Morris et al (2016) | Terrestrial Species | Terrestrial Species |
| Pri-007 | turkey | 5.24 | -18.33 | Morris et al (2016) | Terrestrial Species | Terrestrial Species |
| Rif-062 | turkey | 7.12 | -20.63 | Morris et al (2016) | Terrestrial Species | Terrestrial Species |
| Rif-080 | turkey | 6.81 | -22.34 | Morris et al (2016) | Terrestrial Species | Terrestrial Species |
| Rif-092 | turkey | 6.78 | -19.8 | Morris et al (2016) | Terrestrial Species | Terrestrial Species |
| Rif-107 | turkey | 5.18 | -22.96 | Morris et al (2016) | Terrestrial Species | Terrestrial Species |
| Tho-035 | turkey | 0.03 | -22.36 | Morris et al (2016) | Terrestrial Species | Terrestrial Species |
| Tho-046 | turkey | 5.86 | -22.31 | Morris et al (2016) | Terrestrial Species | Terrestrial Species |
| Tho-054 | turkey | 4 | -21.78 | Morris et al (2016) | Terrestrial Species | Terrestrial Species |
| Tho-058 | turkey | 4.87 | -22.22 | Morris et al (2016) | Terrestrial Species | Terrestrial Species |
| Tho-065 | turkey | 6.6 | -22.16 | Morris et al (2016) | Terrestrial Species | Terrestrial Species |
| Van-011 | turkey | 5.56 | -21.33 | Morris et al (2016) | Terrestrial Species | Terrestrial Species |
| Van-012 | turkey | 6.76 | -21.32 | Morris et al (2016) | Terrestrial Species | Terrestrial Species |
| Van-017 | turkey | 6.31 | -20.93 | Morris et al (2016) | Terrestrial Species | Terrestrial Species |
| Wal-050 | turkey | 5.46 | -20.19 | Morris et al (2016) | Terrestrial Species | Terrestrial Species |
| Wal-051 | turkey | 5.63 | -21.95 | Morris et al (2016) | Terrestrial Species | Terrestrial Species |
| Win-047 | turkey | 6.92 | -19.55 | Morris et al (2016) | Terrestrial Species | Terrestrial Species |
| Win-221 | turkey | 6.11 | -19.02 | Morris et al (2016) | Terrestrial Species | Terrestrial Species |
| Anguilla rostrata (American eel) | Catadromous Fish | 7.8 | -15.2 | Pfeiffer et al. (2016) | Fish | Medium N Fish |
| Anguilla rostrata (American eel) | Catadromous Fish | 10.2 | -23.5 | Pfeiffer et al. (2016) | Fish | Medium N Fish |
| Anguilla rostrata (American eel) | Catadromous Fish | 8.6 | -14.6 | Pfeiffer et al. (2016) | Fish | Medium N Fish |
| Anguilla rostrata (American eel) | Catadromous Fish | 9.2 | -17.2 | Pfeiffer et al. (2016) | Fish | Medium N Fish |
| Anguilla rostrata (American eel) | Catadromous Fish | 9.8 | -25.8 | Pfeiffer et al. (2016) | Fish | Medium N Fish |
| Anguilla rostrata (American eel) | Catadromous Fish | 6.4 | -22.5 | Pfeiffer et al. (2016) | Fish | Medium N Fish |
| Anguilla rostrata (American eel) | Catadromous Fish | 8.3 | -15.9 | Pfeiffer et al. (2016) | Fish | Medium N Fish |
| Anguilla rostrata (American eel) | Catadromous Fish | 9.2 | -17.1 | Pfeiffer et al. (2016) | Fish | Medium N Fish |
| Anguilla rostrata (American eel) | Catadromous Fish | 10.5 | -16.9 | Pfeiffer et al. (2016) | Fish | Medium N Fish |
| Anguilla rostrata (American eel) | Catadromous Fish | 10.5 | -16.9 | Pfeiffer et al. (2016) | Fish | Medium N Fish |
| Anguilla rostrata (American eel) | Catadromous Fish | 8.1 | -16.2 | Pfeiffer et al. (2016) | Fish | Medium N Fish |
| Salmonidae (2) | Salmonidae | 10.1 | -19.4 | Pfeiffer et al. (2016) | Fish | High N Fish |
| Salmo salar (Atlantic salmon) | Salmonidae | 10.4 | -19.7 | Pfeiffer et al. (2016) | Fish | High N Fish |
| Salmo salar (Atlantic salmon) | Salmonidae | 10.7 | -19.2 | Pfeiffer et al. (2016) | Fish | High N Fish |
| Salmo salar (Atlantic salmon) | Salmonidae | 10.2 | -19.6 | Pfeiffer et al. (2016) | Fish | High N Fish |
| Salmo salar (Atlantic salmon) | Salmonidae | 9.6 | -19.1 | Pfeiffer et al. (2016) | Fish | High N Fish |
| Salmo salar (Atlantic salmon) | Salmonidae | 10.7 | -19.6 | Pfeiffer et al. (2016) | Fish | High N Fish |
| Salmo salar (Atlantic salmon) | Salmonidae | 9.9 | -19.3 | Pfeiffer et al. (2016) | Fish | High N Fish |
| Salmo salar (Atlantic salmon) | Salmonidae | 10.1 | -19.3 | Pfeiffer et al. (2016) | Fish | High N Fish |
| Salvelinus namaycush (Lake trout) | Salmonidae | 9.7 | -19.5 | Pfeiffer et al. (2016) | Fish | High N Fish |
| Salvelinus namaycush (Lake trout) | Salmonidae | 12.1 | -20.7 | Pfeiffer et al. (2016) | Fish | High N Fish |
| Salvelinus namaycush (Lake trout) | Salmonidae | 11 | -20.3 | Pfeiffer et al. (2016) | Fish | High N Fish |
| Salvelinus namaycush (Lake trout) | Salmonidae | 11.8 | -21 | Pfeiffer et al. (2016) | Fish | High N Fish |
| Coregonus clupeaformis (Lake whitefish) | Salmonidae | 8.8 | -20.6 | Pfeiffer et al. (2016) | Fish | High N Fish |
| Coregonus clupeaformis (Lake whitefish) | Salmonidae | 8.3 | -21.7 | Pfeiffer et al. (2016) | Fish | High N Fish |
| Coregonus clupeaformis (Lake whitefish) | Salmonidae | 8.8 | -21.3 | Pfeiffer et al. (2016) | Fish | High N Fish |
| Coregonus clupeaformis (Lake whitefish) | Salmonidae | 7.6 | -20.9 | Pfeiffer et al. (2016) | Fish | High N Fish |
| 9350 | Salmonidae | 10.06 | -19.89 | Guiry et al. (2016) | Fish | High N Fish |
| 9351 | Salmonidae | 10.23 | -19.67 | Guiry et al. (2016) | Fish | High N Fish |
| 9352 | Salmonidae | 10.44 | -19.73 | Guiry et al. (2016) | Fish | High N Fish |
| 9353 | Salmonidae | 9.97 | -20.28 | Guiry et al. (2016) | Fish | High N Fish |
| 9354 | Salmonidae | 9.59 | -20.65 | Guiry et al. (2016) | Fish | High N Fish |
| 9354 | Salmonidae | 9.7 | -20.71 | Guiry et al. (2016) | Fish | High N Fish |
| 9355 | Salmonidae | 10.42 | -20.1 | Guiry et al. (2016) | Fish | High N Fish |
| 9356 | Salmonidae | 10.79 | -20.28 | Guiry et al. (2016) | Fish | High N Fish |
| 9357 | Salmonidae | 11.01 | -20.35 | Guiry et al. (2016) | Fish | High N Fish |
| 9359 | Salmonidae | 10.18 | -20 | Guiry et al. (2016) | Fish | High N Fish |
| 9361 | Salmonidae | 10.31 | -20.49 | Guiry et al. (2016) | Fish | High N Fish |
| 9364 | Salmonidae | 10.49 | -19.64 | Guiry et al. (2016) | Fish | High N Fish |
| 10254 | Salmonidae | 10.03 | -22.12 | Guiry et al. (2016) | Fish | High N Fish |
| 10255 | Salmonidae | 9.9 | -22.2 | Guiry et al. (2016) | Fish | High N Fish |
| 10256 | Salmonidae | 10.28 | -22.68 | Guiry et al. (2016) | Fish | High N Fish |
| 10257 | Salmonidae | 10.9 | -22.56 | Guiry et al. (2016) | Fish | High N Fish |
| 10258 | Salmonidae | 13.72 | -15.11 | Guiry et al. (2016) | Fish | High N Fish |
| 10259 | Salmonidae | 11.77 | -15.38 | Guiry et al. (2016) | Fish | High N Fish |
| 10260 | Salmonidae | 10.53 | -15.16 | Guiry et al. (2016) | Fish | High N Fish |
| 10261 | Salmonidae | 10.72 | -21.9 | Guiry et al. (2016) | Fish | High N Fish |
| 10262 | Salmonidae | 10.33 | -22.1 | Guiry et al. (2016) | Fish | High N Fish |
| 10642 | Salmonidae | 13.25 | -18.85 | Guiry et al. (2016) | Fish | High N Fish |
| 10643 | Salmonidae | 13.07 | -19.91 | Guiry et al. (2016) | Fish | High N Fish |
| 10649 | Salmonidae | 10.28 | -19.62 | Guiry et al. (2016) | Fish | High N Fish |
| 10650 | Salmonidae | 10.3 | -19.61 | Guiry et al. (2016) | Fish | High N Fish |
| 10651 | Salmonidae | 10 | -20.31 | Guiry et al. (2016) | Fish | High N Fish |
| 10652 | Salmonidae | 10.46 | -20.73 | Guiry et al. (2016) | Fish | High N Fish |
| 10653 | Salmonidae | 10.19 | -19.67 | Guiry et al. (2016) | Fish | High N Fish |
| 10654 | Salmonidae | 10.12 | -20.1 | Guiry et al. (2016) | Fish | High N Fish |
| 10655 | Salmonidae | 10.19 | -20.32 | Guiry et al. (2016) | Fish | High N Fish |
| 10656 | Salmonidae | 10.2 | -20.34 | Guiry et al. (2016) | Fish | High N Fish |
| 10657 | Salmonidae | 10.15 | -19.83 | Guiry et al. (2016) | Fish | High N Fish |
| 10658 | Salmonidae | 12.9 | -19.44 | Guiry et al. (2016) | Fish | High N Fish |
| 10659 | Salmonidae | 14.75 | -18.92 | Guiry et al. (2016) | Fish | High N Fish |
| 10660 | Salmonidae | 12.91 | -19.42 | Guiry et al. (2016) | Fish | High N Fish |
| 10661 | Salmonidae | 12.92 | -18.73 | Guiry et al. (2016) | Fish | High N Fish |
| 10664 | Salmonidae | 10.53 | -20.02 | Guiry et al. (2016) | Fish | High N Fish |
| 10665 | Salmonidae | 10.18 | -20.15 | Guiry et al. (2016) | Fish | High N Fish |
| 10666 | Salmonidae | 9.74 | -20.22 | Guiry et al. (2016) | Fish | High N Fish |
| 10667 | Salmonidae | 10.32 | -20.01 | Guiry et al. (2016) | Fish | High N Fish |
| 10668 | Salmonidae | 10.39 | -19.96 | Guiry et al. (2016) | Fish | High N Fish |
| 10669 | Salmonidae | 10.06 | -20.05 | Guiry et al. (2016) | Fish | High N Fish |
| 10670 | Salmonidae | 10.72 | -20.13 | Guiry et al. (2016) | Fish | High N Fish |
| 10671 | Salmonidae | 10.83 | -19.92 | Guiry et al. (2016) | Fish | High N Fish |
| 10672 | Salmonidae | 9.79 | -20.84 | Guiry et al. (2016) | Fish | High N Fish |
| 10673 | Salmonidae | 10.77 | -20.1 | Guiry et al. (2016) | Fish | High N Fish |
| 10674 | Salmonidae | 10.76 | -19.63 | Guiry et al. (2016) | Fish | High N Fish |
| 10675 | Salmonidae | 10.36 | -19.84 | Guiry et al. (2016) | Fish | High N Fish |
| 10676 | Salmonidae | 10.1 | -19.77 | Guiry et al. (2016) | Fish | High N Fish |
| 10677 | Salmonidae | 10.12 | -20.9 | Guiry et al. (2016) | Fish | High N Fish |
| 10678 | Salmonidae | 10.23 | -19.63 | Guiry et al. (2016) | Fish | High N Fish |
| 10679 | Salmonidae | 10.56 | -19.84 | Guiry et al. (2016) | Fish | High N Fish |
| 10680 | Salmonidae | 10.55 | -19.36 | Guiry et al. (2016) | Fish | High N Fish |
| 10681 | Salmonidae | 10.5 | -19.83 | Guiry et al. (2016) | Fish | High N Fish |
| 10682 | Salmonidae | 10.02 | -19.71 | Guiry et al. (2016) | Fish | High N Fish |
| 10683 | Salmonidae | 10.36 | -20.1 | Guiry et al. (2016) | Fish | High N Fish |
| 10684 | Salmonidae | 10.08 | -19.95 | Guiry et al. (2016) | Fish | High N Fish |
| 10685 | Salmonidae | 10.36 | -19.66 | Guiry et al. (2016) | Fish | High N Fish |
| 10687 | Salmonidae | 10.23 | -19.55 | Guiry et al. (2016) | Fish | High N Fish |
| 10688 | Salmonidae | 10.57 | -20.57 | Guiry et al. (2016) | Fish | High N Fish |
| 10689 | Salmonidae | 10.05 | -20.13 | Guiry et al. (2016) | Fish | High N Fish |
| 11703 | Salmonidae | 9.94 | -15.26 | Guiry et al. (2016) | Fish | High N Fish |
| 11704 | Salmonidae | 9.84 | -15.87 | Guiry et al. (2016) | Fish | High N Fish |
| 11705 | Salmonidae | 9.96 | -16.63 | Guiry et al. (2016) | Fish | High N Fish |
| 11706 | Salmonidae | 9.92 | -16.6 | Guiry et al. (2016) | Fish | High N Fish |
| 11707 | Salmonidae | 9.81 | -15.96 | Guiry et al. (2016) | Fish | High N Fish |
| 10538-1 | Salmonidae | 11.45 | -15.7 | Guiry et al. (2016) | Fish | High N Fish |
| 10538-2 | Salmonidae | 11.47 | -15.53 | Guiry et al. (2016) | Fish | High N Fish |
| 10539-1 | Salmonidae | 11.5 | -15.75 | Guiry et al. (2016) | Fish | High N Fish |
| 10539-2 | Salmonidae | 11.52 | -15.72 | Guiry et al. (2016) | Fish | High N Fish |
| 10540-1 | Salmonidae | 12.53 | -20.06 | Guiry et al. (2016) | Fish | High N Fish |
| 10540-2 | Salmonidae | 12.55 | -19.93 | Guiry et al. (2016) | Fish | High N Fish |
| 10541-1 | Salmonidae | 13.68 | -19.55 | Guiry et al. (2016) | Fish | High N Fish |
| 10541-2 | Salmonidae | 12.47 | -19.98 | Guiry et al. (2016) | Fish | High N Fish |
| 10542-1 | Salmonidae | 13.05 | -19.82 | Guiry et al. (2016) | Fish | High N Fish |
| 10542-2 | Salmonidae | 12.54 | -19.7 | Guiry et al. (2016) | Fish | High N Fish |
| 10642 | Salmonidae | 13.25 | -18.85 | Guiry et al. (2016) | Fish | High N Fish |
| 10643 | Salmonidae | 13.07 | -19.91 | Guiry et al. (2016) | Fish | High N Fish |
| Coregonus clupeaformis (Lake whitefish) | Salmonidae | 8.5 | -21.1 | Pfeiffer et al. (2016) | Fish | High N Fish |
| Amia calva (Bowfin) | Nominally piscivorous Fish | 8 | -23.4 | Pfeiffer et al. (2016) | Fish | Medium N Fish |
| Amia calva (Bowfin) | Nominally piscivorous Fish | 7.6 | -23.7 | Pfeiffer et al. (2016) | Fish | Medium N Fish |
| Ameiurus nebulosus (Brown bullhead) | Nominally piscivorous Fish | 7.1 | -21.1 | Pfeiffer et al. (2016) | Fish | Medium N Fish |
| Ameiurus nebulosus (Brown bullhead) | Nominally piscivorous Fish | 7.2 | -23 | Pfeiffer et al. (2016) | Fish | Medium N Fish |
| Ameiurus nebulosus (Brown bullhead) | Nominally piscivorous Fish | 5.8 | -18.5 | Pfeiffer et al. (2016) | Fish | Medium N Fish |
| Ameiurus nebulosus (Brown bullhead) | Nominally piscivorous Fish | 7 | -19.9 | Pfeiffer et al. (2016) | Fish | Medium N Fish |
| Ameiurus nebulosus (Brown bullhead) | Nominally piscivorous Fish | 6.3 | -20.8 | Pfeiffer et al. (2016) | Fish | Medium N Fish |
| Ameiurus nebulosus (Brown bullhead) | Nominally piscivorous Fish | 5.6 | -18.1 | Pfeiffer et al. (2016) | Fish | Medium N Fish |
| Esox americanus (Grass pickerel) | Piscivorous Fish | 7.5 | -23.6 | Pfeiffer et al. (2016) | Fish | Medium N Fish |
| Esox americanus (Grass pickerel) | Piscivorous Fish | 7.8 | -22.9 | Pfeiffer et al. (2016) | Fish | Medium N Fish |
| Esox lucius (Northern pike) | Piscivorous Fish | 8.8 | -20.8 | Pfeiffer et al. (2016) | Fish | Medium N Fish |
| Esox lucius (Northern pike) | Piscivorous Fish | 9.4 | -18.2 | Pfeiffer et al. (2016) | Fish | Medium N Fish |
| Esox lucius (Northern pike) | Piscivorous Fish | 8.6 | -20.6 | Pfeiffer et al. (2016) | Fish | Medium N Fish |
| Esox lucius (Northern pike) | Piscivorous Fish | 9.3 | -18.3 | Pfeiffer et al. (2016) | Fish | Medium N Fish |
| Esox lucius (Northern pike) | Piscivorous Fish | 9.8 | -18.7 | Pfeiffer et al. (2016) | Fish | Medium N Fish |
| Micropterus dolomieu (Smallmouth bass) | Nominally piscivorous Fish | 8.9 | -17.3 | Pfeiffer et al. (2016) | Fish | Medium N Fish |
| Micropterus dolomieu (Smallmouth bass) | Nominally piscivorous Fish | 9.1 | -17.6 | Pfeiffer et al. (2016) | Fish | Medium N Fish |
| Micropterus salmoides (Largemouth bass) | Nominally piscivorous Fish | 8.5 | -18.8 | Pfeiffer et al. (2016) | Fish | Medium N Fish |
| Micropterus salmoides (Largemouth bass) | Nominally piscivorous Fish | 8 | -20 | Pfeiffer et al. (2016) | Fish | Medium N Fish |
| Micropterus salmoides (Largemouth bass) | Nominally piscivorous Fish | 8.4 | -19.2 | Pfeiffer et al. (2016) | Fish | Medium N Fish |
| Micropterus salmoides (Largemouth bass) | Nominally piscivorous Fish | 7.2 | -23.1 | Pfeiffer et al. (2016) | Fish | Medium N Fish |
| Micropterus salmoides (Largemouth bass) | Nominally piscivorous Fish | 7.9 | -18.9 | Pfeiffer et al. (2016) | Fish | Medium N Fish |
| Perca flavescens (Yellow perch) | Nominally piscivorous Fish | 10.2 | -19 | Pfeiffer et al. (2016) | Fish | Medium N Fish |
| Perca flavescens (Yellow perch) | Nominally piscivorous Fish | 10.2 | -21.4 | Pfeiffer et al. (2016) | Fish | Medium N Fish |
| Perca flavescens (Yellow perch) | Nominally piscivorous Fish | 9.9 | -17.8 | Pfeiffer et al. (2016) | Fish | Medium N Fish |
| Perca flavescens (Yellow perch) | Nominally piscivorous Fish | 8.8 | -19 | Pfeiffer et al. (2016) | Fish | Medium N Fish |
| Perca flavescens (Yellow perch) | Nominally piscivorous Fish | 8.6 | -24.1 | Pfeiffer et al. (2016) | Fish | Medium N Fish |
| Perca flavescens (Yellow perch) | Nominally piscivorous Fish | 9.3 | -19 | Pfeiffer et al. (2016) | Fish | Medium N Fish |
| Perca flavescens (Yellow perch) | Nominally piscivorous Fish | 9.2 | -17.4 | Pfeiffer et al. (2016) | Fish | Medium N Fish |
| Perca flavescens (Yellow perch) | Nominally piscivorous Fish | 9.3 | -18.5 | Pfeiffer et al. (2016) | Fish | Medium N Fish |
| Stizostedion sp. (Walleye or sauger) | Nominally piscivorous Fish | 11.7 | -17.4 | Pfeiffer et al. (2016) | Fish | Medium N Fish |
| Sander vitreus (Walleye) | Nominally piscivorous Fish | 8 | -16.3 | Pfeiffer et al. (2016) | Fish | Medium N Fish |
| Lota lota (Burbot) | Oily liver Fish | 12 | -21 | Pfeiffer et al. (2016) | Fish | High N Fish |
| Lota lota (Burbot) | Oily liver Fish | 12.1 | -19.4 | Pfeiffer et al. (2016) | Fish | High N Fish |
| Lota lota (Burbot) | Oily liver Fish | 14 | -20.2 | Pfeiffer et al. (2016) | Fish | High N Fish |
| Lota lota (Burbot) | Oily liver Fish | 11.3 | -21.1 | Pfeiffer et al. (2016) | Fish | High N Fish |
| Lota lota (Burbot) | Oily liver Fish | 10.5 | -16.9 | Pfeiffer et al. (2016) | Fish | High N Fish |
| Lota lota (Burbot) | Oily liver Fish | 12.7 | -20.6 | Pfeiffer et al. (2016) | Fish | High N Fish |
| Lota lota (Burbot) | Oily liver Fish | 10.2 | -19.4 | Pfeiffer et al. (2016) | Fish | High N Fish |
| Lota lota (Burbot) | Oily liver Fish | 12.8 | -19.5 | Pfeiffer et al. (2016) | Fish | High N Fish |
| Lota lota (Burbot) | Oily liver Fish | 12 | -19.7 | Pfeiffer et al. (2016) | Fish | High N Fish |
| Catostomus sp. (Sucker) | Non-piscivorous fish | 7.1 | -18.8 | Pfeiffer et al. (2016) | Fish | Low N Fish |
| Catostomus sp. (Sucker) | Non-piscivorous fish | 6.6 | -18.4 | Pfeiffer et al. (2016) | Fish | Low N Fish |
| Catostomus sp. (Sucker) | Non-piscivorous fish | 3.6 | -23.1 | Pfeiffer et al. (2016) | Fish | Low N Fish |
| Catostomus catostomus (Longnose sucker) | Non-piscivorous fish | 5.8 | -17.5 | Pfeiffer et al. (2016) | Fish | Low N Fish |
| Catostomus commersoni (White sucker) | Non-piscivorous fish | 6 | -22.2 | Pfeiffer et al. (2016) | Fish | Low N Fish |
| Catostomus commersoni (White sucker) | Non-piscivorous fish | 5.4 | -18.5 | Pfeiffer et al. (2016) | Fish | Low N Fish |
| Catostomus commersoni (White sucker) | Non-piscivorous fish | 5.5 | -18.2 | Pfeiffer et al. (2016) | Fish | Low N Fish |
| Catostomus commersoni (White sucker) | Non-piscivorous fish | 5.1 | -16.7 | Pfeiffer et al. (2016) | Fish | Low N Fish |
| Ictalurus cf. punctatus (probable Channel catfish) | Non-piscivorous fish | 8.1 | -17.4 | Pfeiffer et al. (2016) | Fish | Low N Fish |
| Ictalurus cf. punctatus (probable Channel catfish) | Non-piscivorous fish | 9.1 | -17.4 | Pfeiffer et al. (2016) | Fish | Low N Fish |
| (Sunfish) | Non-piscivorous fish | 9.1 | -18.2 | Pfeiffer et al. (2016) | Fish | Low N Fish |
| Ambloplites rupestris (Rock bass) | Non-piscivorous fish | 8 | -24.9 | Pfeiffer et al. (2016) | Fish | Low N Fish |
| Ambloplites rupestris (Rock bass) | Non-piscivorous fish | 8.3 | -20.9 | Pfeiffer et al. (2016) | Fish | Low N Fish |
| Ambloplites rupestris (Rock bass) | Non-piscivorous fish | 8.6 | -21.6 | Pfeiffer et al. (2016) | Fish | Low N Fish |
| Ambloplites rupestris (Rock bass) | Non-piscivorous fish | 7.8 | -21.1 | Pfeiffer et al. (2016) | Fish | Low N Fish |
| Ambloplites rupestris (Rock bass) | Non-piscivorous fish | 8.7 | -22.4 | Pfeiffer et al. (2016) | Fish | Low N Fish |
| Lepomis gibbosus (Pumpkinseed) | Non-piscivorous fish | 6.6 | -15.8 | Pfeiffer et al. (2016) | Fish | Low N Fish |
| Lepomis gibbosus (Pumpkinseed) | Non-piscivorous fish | 7.2 | -23.2 | Pfeiffer et al. (2016) | Fish | Low N Fish |
| Lepomis gibbosus (Pumpkinseed) | Non-piscivorous fish | 7.1 | -17.6 | Pfeiffer et al. (2016) | Fish | Low N Fish |
| Lepomis gibbosus (Pumpkinseed) | Non-piscivorous fish | 4.4 | -20.3 | Pfeiffer et al. (2016) | Fish | Low N Fish |
| Pomoxis cf. nigromaculatus | Non-piscivorous fish | 7.9 | -20.1 | Pfeiffer et al. (2016) | Fish | Low N Fish |
| Pomoxis cf. nigromaculatus | Non-piscivorous fish | 8 | -21.5 | Pfeiffer et al. (2016) | Fish | Low N Fish |
| Aplodinotus grunniens (Freshwater drum) | Non-piscivorous fish | 6.1 | -11.5 | Pfeiffer et al. (2016) | Fish | Low N Fish |
| UGAMS-32755 | Coulter | 5.47 | -9.47 | This study | Maize | Maize |
| UGAMS-32756 | Coulter | 3.33 | -9.31 | This study | Maize | Maize |
| UGAMS-32991 | Baker | 5.98 | -9.40 | This study | Maize | Maize |
| UGAMS-32992 | Baker | 3.90 | -9.31 | This study | Maize | Maize |
| UGAMS-32994 | McNair | 4.10 | -10.32 | This study | Maize | Maize |
| UGAMS-32995 | McNair | 4.36 | -9.70 | This study | Maize | Maize |
| UGAMS-33005 | Damiani | 4.71 | -9.64 | This study | Maize | Maize |
| UGAMS-33006 | Damiani | 3.97 | -8.89 | This study | Maize | Maize |
| UGAMS-33008 | Parsons | 4.03 | -9.24 | This study | Maize | Maize |
| UGAMS-33009 | Parsons | 6.03 | -9.81 | This study | Maize | Maize |
| UGAMS-37833 | Spang | 4.69 | -9.65 | This study | Maize | Maize |
| UGAMS-37834 | Spang | 6.53 | -8.73 | This study | Maize | Maize |
| UGAMS-38397 | Spang | 5.43 | -9.44 | This study | Maize | Maize |
| UGAMS-38398 | Spang | 6.99 | -8.22 | This study | Maize | Maize |

**S.3. Bone and dentine collagen isotope values for 14^th^ to 17^th^ century Wendat people**

Site name, tooth/bone identification number, stable isotope values, and carbon/nitrogen isotope ratios for all human samples included in this study. All data derive from Pfeiffer et al. (2016).

| **Site Name** | **ID** | **Tooth/Bone** | **δ13C Dentine** | **δ13C Bone** | **δ15N Dentine** | **δ15N Bone** | **C/N Dentine** | **C/N Bone** |
| --- | --- | --- | --- | --- | --- | --- | --- | --- |
| Staines | R max M2 | Sample 1 | -9.7 | -11.9 | 12.0 | 12.0 | 3.20 | 3.40 |
| Staines | R max M1 | Sample 2 | -11.3 |  | 12.6 |  | 3.30 | 4.10 |
| Staines | R mand M1 | Sample 3 | -10.7 | -11.9 | 11.8 | 12.0 | 3.20 | 3.30 |
| Staines | R max M1 | Sample 4 | -9.2 | -12.5 | 10.9 | 12.8 | 3.20 | 3.30 |
| Staines | R mand M1 | Sample 5 | -11.3 |  | 12.0 |  | 3.20 |  |
| Staines | L max M1 | Sample 6 | -11.4 |  | 12.3 |  | 3.20 |  |
| Staines | L mand M1 | Sample 7 | -10.9 | -14.1 | 11.3 | 10.6 | 3.20 | 3.30 |
| Staines | R mand M1 | Sample 8 | -11.9 |  | 12.7 |  | 3.20 |  |
| Staines | R mand M2 | Sample 9 | -13.4 |  | 11.6 |  | 3.30 |  |
| Staines | R max M1 | Sample 10 | -10.2 |  | 11.5 |  | 3.20 |  |
| Staines | L mand M1 | Sample 11 | -10.5 |  | 11.4 |  | 3.30 |  |
| Staines | R max M1 | Sample 12 | -11.4 |  | 12.9 |  | 3.20 |  |
| Staines | R mand M2 | Sample 13 | -9.6 |  | 11.3 |  | 3.20 |  |
| Staines | R max M1 | Sample 14 | -13.2 |  | 11.7 |  | 3.20 |  |
| Staines | R max M1 | Sample 15 | -12.0 |  | 12.5 |  | 3.20 |  |
| Moatfield | LM1 | 6 | -10.3 | -11.6 | 11.5 | 11.6 |  |  |
| Moatfield | LM2 | L8D9 | -11.4 |  | 12.6 |  |  |  |
| Moatfield | RM1 | 1485 | -10.4 |  | 11.0 |  |  |  |
| Moatfield | RM2 | 754 | -12.5 |  | 13.7 |  |  |  |
| Moatfield | RM2 | 1571 | -11.5 |  | 12.8 |  |  |  |
| Moatfield | LM3 | 546 | -10.4 |  | 11.7 |  |  |  |
| Moatfield | RM1 | 2014 | -11.8 |  | 13.0 |  |  |  |
| Moatfield | RM3 | 5 | -10.0 |  | 12.0 |  |  |  |
| Moatfield | LM2 | 8 | -11.3 |  | 12.9 |  |  |  |
| Moatfield | LM3 | 12 | -10.6 |  | 13.5 |  |  |  |
| Moatfield | RM1 | 15 | -9.5 |  | 11.4 |  |  |  |
| Moatfield | RM3 | 20 | -10.3 |  | 12.7 |  |  |  |
| Moatfield | RM3 | 24 | -9.6 |  | 12.3 |  |  |  |
| Moatfield | RM3 | 561 | -9.4 | -12.0 | 12.0 | 11.7 |  |  |
| Moatfield | RM | 2629 | -10.6 |  | 12.7 |  |  |  |
| Moatfield | RM2 | 740a | -9.3 | -10.2 | 11.8 | 11.7 |  |  |
| Moatfield | RM2 | 1000d | -10.4 |  | 13.0 |  |  |  |
| Moatfield | RM2 | 1150 | -13.0 |  | 13.8 |  |  |  |
| Moatfield | RM2 | 1198 | -13.0 | -11.6 | 13.4 | 12.5 |  |  |
| Moatfield | RM2 | 1895 | -11.3 |  | 11.1 |  |  |  |
| Moatfield | RM2 | 1442 | -11.3 |  | 11.7 |  |  |  |
| Moatfield | LM1 | 1652a | -10.0 |  | 11.8 |  |  |  |
| Moatfield | LM1 | 1700a | -11.3 | -10.8 | 13.1 | 11.9 |  |  |
| Moatfield | LP2 | 2044a | -13.8 |  | 13.3 |  |  |  |
| Moatfield | RM3 | 683 | -9.8 | -14.4 | 11.9 | 11.9 |  |  |
| Moatfield | RM3 | 756 | -10.8 | -13.2 | 12.4 | 12.7 |  |  |
| Moatfield | LPM1 | 2 | -10.9 |  | 13.1 |  |  |  |
| Moatfield | LPM1 | 3 | -13.2 |  | 13.4 |  |  |  |
| Moatfield | RM2 | 7 | -10.7 | -12.9 | 11.9 | 12.5 |  |  |
| Moatfield | RM1 | 18 | -15.4 |  | 14.3 |  |  |  |
| Moatfield | RM3 | 595 | -11.3 |  | 12.1 |  |  |  |
| Moatfield | LM1 | 9 | -14.3 | -13.9 | 12.9 | 12.1 |  |  |
| Moatfield | LC | 21 |  |  |  |  |  |  |
| Moatfield | RM3 | 492a | -9.1 |  | 12.4 |  |  |  |
| Moatfield | RM3 | 492b |  |  |  |  |  |  |
| Moatfield | LM2 | 493 | -11.9 |  | 12.8 |  |  |  |
| Moatfield | LM3 | 828 | -11.3 | -11.0 | 13.3 | 13.2 |  |  |
| Moatfield | LM3 | 1430 | -12.8 |  | 13.0 |  |  |  |
| Moatfield | RM1 | 1000e | -12.0 |  | 13.3 |  |  |  |
| Moatfield | LM1 | 1652b | -11.6 |  | 12.5 |  |  |  |
| Moatfield | RM1 | 1666 | -10.4 |  | 15.5 |  |  |  |
| Moatfield | RM1 | 1790a | -12.3 |  | 12.6 |  |  |  |
| Moatfield | LM3 | 1866 | -13.7 | -13.2 | 13.0 | 14.0 |  |  |
| Moatfield | RM1 | 2079 | -10.2 |  | 11.9 |  |  |  |
| Hutchinson | L max M3 | B1 | -11.4 |  | 12.4 |  | 3.20 |  |
| Hutchinson | R max M3 | B2 | -10.5 |  | 13.8 |  | 3.10 |  |
| Hutchinson | R max M2 | B2a | -10.5 | -12.2 | 12.0 | 10.4 | 3.20 | 3.40 |
| Hutchinson | L mand M2 | F88 | -11.6 |  | 13.4 |  | 3.10 |  |
| Hutchinson | R max M3 | F92 | -13.4 |  | 13.2 |  | 3.30 |  |
| Fairty AlGt-3 | cranmand0022 (43) | 14138 | -9.8 |  | 12.7 |  | 3.30 |  |
| Fairty AlGt-3 | cranmand0010 (817) | 14139 | -10.2 |  | 11.4 |  | 3.20 |  |
| Fairty AlGt-3 | cranmand0024 (318) | 114140 | -10.7 |  | 11.4 |  | 3.30 |  |
| Fairty AlGt-3 | cranmand0006 (739) | 14141 | -10.8 | -10.6 | 10.8 | 10.9 | 3.20 | 3.30 |
| Fairty AlGt-3 | cranmand0041 (126) | 14142 | -8.6 | -10.5 | 12.0 | 11.9 | 3.30 | 3.30 |
| Fairty AlGt-3 | cranmand0027 (301) | 14143 | -11.8 |  | 12.0 |  | 3.20 |  |
| Fairty AlGt-3 | cranmand0023 (47) | 14144 | -11.2 | -11.4 | 11.6 | 12.1 | 3.30 | 3.30 |
| Fairty AlGt-3 | cranmand0053 (54) | 14145 | -10.6 | -14.2 | 11.6 | 10.9 | 3.20 |  |
| Fairty AlGt-3 | cranmand0038 (52) | 14146 | -10.0 | -10.2 | 11.8 | 11.9 | 3.20 | 3.30 |
| Fairty AlGt-3 | cranmand0014 (738) | 14147 | -13.4 |  | 11.9 |  | 3.70 |  |
| Fairty AlGt-3 | cranmand0149 (479) | 16045 [3] | -11.2 | -11.2 | 11.5 |  | 3.50 |  |
| Fairty AlGt-3 | cranmand0157 (25) | 16046 [3] | -11.2 | -10.0 | 12.4 | 11.5 | 3.40 | 3.50 |
| Fairty AlGt-3 | cranmand0156 (153) | 16047 [3] | -10.9 | -10.8 | 11.2 | 11.5 | 3.20 | 3.40 |
| Fairty AlGt-3 | cranmand0062 (102) | 16048 [3] | -11.3 |  | 10.8 |  | 3.40 |  |
| Fairty AlGt-3 | cranmand0034 (MUI2) | 16049 [3] | -9.6 |  | 11.6 |  | 3.30 |  |
| Uxbridge | cranmand0168 (U3L9S8 162) | 114173 | -11.3 | -13.2 | 9.9 | 13.8 | 3.20 | 3.40 |
| Uxbridge | cranmand0164 (U2L9S8 369) | 14174 | -10.6 |  | 11.1 |  | 3.20 |  |
| Uxbridge | cranmand0085 (U2L9S6 232) | 14175 | -9.9 | -11.9 | 11.7 | 14.1 | 3.20 | 3.40 |
| Uxbridge | cranmand0063 (U2L5S4 166) | 14176 | -10.8 |  | 11.2 |  | 3.30 |  |
| Uxbridge | cranmand0004 (L19S1 87) | 14177 | -10.3 |  | 12.2 |  | 3.30 |  |
| Uxbridge | cranmand 0114 (U2L11S5 260) | 14178 | -11.8 |  | 11.5 |  | 3.20 |  |
| Uxbridge | cranmand 0125 (U1L12S3 81) | 14179 | -13.4 |  | 12.8 |  | 3.30 |  |
| Uxbridge | cranmand0037 (U3L10S1 389) | 14180 | -11.7 |  | 11.5 |  | 3.30 |  |
| Uxbridge | cranmand0027 (U3L11S8 406) | 14181 | -10.9 |  | 11.8 |  | 3.30 |  |
| Uxbridge | cranmand0007 (L20S3 95) | 14182 | -11.1 | -10.4 | 11.7 | 15.1 | 3.30 | 3.30 |
| Uxbridge | cranmand0040 (U3 L10 S7 397) | 16050 [3] | -9.5 | -12.0 | 10.7 | 12.1 | 3.20 | 3.40 |
| Uxbridge | cranmand0109 (U2 L8 S4) | 16052 [3] | -11.7 |  | 10.8 |  | 3.50 |  |
| Uxbridge | cranmand0069 (U2 L5 S4 A6) | 16054 [3] | -10.7 | -11.8 | 10.7 | 11.9 | 3.20 | 3.30 |
| Uxbridge | cranmand0129 (U1 F2 S2 ind. 2 A79) | 16057 [3] | -10.2 |  | 12.1 |  | 3.20 |  |
| Uxbridge | cranmand0106 (U2 L8 S4 211) | 16058 [3] | -10.0 | -10.3 | 11.4 | 11.6 | 3.30 | 3.50 |
| Uxbridge | cranmand0022 (East wall 106) | 16059 [3] | -10.1 | -11.7 | 10.5 | 15.8 | 3.20 | 3.20 |
| Teston | L max M2 | Sample 1 | -9.8 |  | 11.2 |  | 3.20 |  |
| Teston | R max M2 | Sample 2 | -10.6 |  | 10.9 |  | 3.20 |  |
| Teston | L max M1 | Sample 3 | -11.7 | -11.9 | 11.6 | 11.0 | 3.20 | 3.40 |
| Teston | L max M1 | Sample 4 | -9.9 |  | 10.4 |  | 3.10 | 4.30 |
| Teston | R max M2 | Sample 5 | -11.3 | -12.5 | 10.9 | 11.8 | 3.20 | 3.30 |
| Teston | L max M1 | Sample 6 | -11.0 | -12.0 | 12.4 | 11.6 | 3.30 | 3.20 |
| Teston | L max M1 | Sample 7 | -11.5 | -12.5 | 11.6 | 10.7 | 3.20 | 3.30 |
| Teston | L max M1 | Sample 8 | -11.6 | 11.9 | 11.0 | 11.3 | 3.30 | 3.20 |
| Teston | L max M1 | Sample 9 | -11.0 |  | 9.8 |  | 3.30 | 4.60 |
| Teston | L max M2 | Sample 10 | -11.4 | -11.8 | 10.9 | 10.9 | 3.20 | 3.20 |
| Bosomworth | cranmand0003 (BwC2) | 14188 | -9.7 |  | 12.1 |  | 3.20 |  |
| Bosomworth | cranmand0004 (BwH2) | 14189 | -12.3 |  | 12.4 |  | 3.20 |  |
| Bosomworth | cranmand0001 (BwE2) | 14190 | -10.6 |  | 12.3 |  | 3.20 |  |
| Hidden Spring | L max M3 | B2a | -10.7 |  | 12.3 |  | 3.20 |  |
| Hidden Spring | R mand dm2 | B2b | -12.8 |  | 10.8 |  | 3.40 |  |
| Hidden Spring | L mand M1 | B3a | -11.9 |  | 12.2 |  | 3.10 |  |
| Hidden Spring | L max M2 | B3b | -10.7 | -13.0 | 11.6 | 11.8 | 3.20 | 3.30 |
| Hidden Spring | L max M2 | B2 | -9.7 |  | 12.0 |  | 3.20 |  |
| Damiani | R max dm1 | B4 | -11.9 |  | 12.2 |  | 3.50 |  |
| Damiani | L mand M1 | B8 | -13.1 |  | 10.6 |  |  |  |
| Mantle-cemetery | L max M1 | B2 | -10.8 |  | 10.6 |  |  |  |
| Mantle-cemetery | R max M1 | B24 | -10.6 |  | 10.4 |  |  |  |
| Mantle-cemetery | R max M1 | B7a | -11.6 |  | 9.9 |  |  |  |
| Mantle-cemetery | R mand PM2 | B7b | -12.7 |  | 12.1 |  |  |  |
| Mantle-cemetery | L max M1 | 435-180 surface | -9.8 |  | 10.2 |  |  |  |
| Mantle-village | R mand M1 | 465-155 layer 1 | -9.9 |  | 10.4 |  |  |  |
| Mantle-village | L max M2 | 435-180 Q5, fill | -9.1 |  | 11.1 |  |  |  |
| Mantle-village | L max C | 485-240 trench | -10.0 |  | 10.5 |  |  |  |
| Mantle-village | L mand PM2 | 495-129 fill | -11.6 |  | 10.7 |  |  |  |
| Mantle-village | L max M1 | 522-141 charcoal | -9.5 |  | 9.8 |  |  |  |
| Mantle-village | R max PM1 | 335-160 fill | -8.8 |  | 10.5 |  |  |  |
| Mantle-village | R mand M3 | 465-155 Q4, layer 1 | -10.7 |  | 10.6 |  |  |  |
| Mantle-village | R max M3 | 500-165 fill | -11.2 |  | 11.5 |  |  |  |
| Mantle-village | R mand M1 | 499-133 fill | -9.2 |  | 10.0 |  |  |  |
| Milne BcHb-28 | cranmand0003 (mm-22 #6)/M2 | 14153 | -10.9 |  | 11.9 |  | 3.30 |  |
| Milne BcHb-28 | cranmand0001 (mm-4)/M3 | 14154 | -9.9 |  | 12.3 |  | 3.30 |  |
| Milne BcHb-28 | cranmand0002 (mm-19) | 14152 | -10.0 | -11.2 | 12.6 | 15.2 | 3.30 | 3.30 |
| Kleinburg | #2030 | 14163 | -11.5 | -13.0 | 12.0 | 12.2 | 3.30 | 3.40 |
| Kleinburg | #190 | 14164 | -8.9 | -11.0 | 11.6 | 12.3 | 3.30 | 3.30 |
| Kleinburg | 18:14 05 | 14165 | -10.4 | -12.3 | 12.5 | 12.7 | 3.20 | 3.30 |
| Kleinburg | #35 | 14166 | -9.0 |  | 11.4 |  | 3.20 |  |
| Kleinburg | #213 | 14167 | -11.4 | -12.5 | 11.9 | 11.7 | 3.20 | 3.30 |
| Kleinburg | #2073 | 14168 | -10.4 | -10.1 | 11.0 | 12.3 | 3.20 | 3.20 |
| Kleinburg | #195 | 14169 | -9.8 |  | 11.9 |  | 3.20 |  |
| Kleinburg | #31 | 14170 | -10.5 | -12.2 | 11.3 | 11.7 | 3.20 | 3.20 |
| Kleinburg | #2070 | 14171 | -10.1 |  | 11.8 |  | 3.30 |  |
| Kleinburg | #2130 | 14172 | -10.4 | -12.0 | 12.1 | 11.9 | 3.20 | 3.30 |
| Kleinburg |  | 16029 [3] | -10.2 |  | 11.7 |  | 3.30 |  |
| Kleinburg |  | 16031 [3] | -10.2 |  | 11.6 |  | 3.30 |  |
|  |  |  |  |  |  |  |  |  |
| Kleinburg |  | 16032 [3] | -9.6 | -11.2 | 12.4 | 12.7 | 3.20 | 3.30 |
| Kleinburg |  | 16033 [3] | -10.3 |  | 11.7 |  | 3.30 |  |
| Kleinburg |  | 16036 [3] | -10.6 | -12.6 | 11.7 | 11.7 | 3.20 | 3.20 |
| Kleinburg |  | 16039 [3] | -10.0 |  | 11.8 |  | 3.30 |  |
| Warminster | cranmand0006 (90) | 14155 | -10.9 |  | 12.2 |  | 3.40 |  |
| Warminster | cranmand0001 (91) | 14156 | -8.9 | -11.5 | 11.1 | 12.0 | 3.40 | 3.50 |
| Warminster | cranmand0021 (114) | 14157 | -10.5 | -12.6 | 12.7 | 12.3 | 3.20 | 3.40 |
| Warminster | no provenience | 14158 [3] | -10.8 |  | 11.9 |  | 3.30 |  |
| Warminster | no provenience | 14159 [3] | -10.4 |  | 12.1 |  | 3.30 |  |
| Warminster | cranmand0013 (115) | 16021 [3] | -11.7 | -11.5 | 11.7 | 11.3 | 3.30 | 2.90 |
| Maurice | cranmand0004 (W8 N4 1292) | 14183 | -11.2 | -12.2 | 13.4 | 14.1 | 3.20 | 3.50 |
| Maurice | cranmand0049 (W10 N4 1121) | 14184 | -10.9 | -13.2 | 13.6 | 13.3 | 3.40 | 3.40 |
| Maurice | cranmand0046 (W8 N4 2003) | 14185 | -9.3 |  | 12.5 |  | 3.20 |  |
| Maurice | cranmand0047 (W8 N4 2004) | 14186 | -10.4 |  | 12.2 |  | 3.20 |  |
| Maurice | cranmand0052 (W10 N4 1129) | 14187 | -10.7 |  | 14.4 |  | 3.20 |  |
| Maurice | W10_N4_L13 | 16024 |  |  |  |  |  |  |
| Maurice | cranmand0041 (W10 N4 1133) | 16025 [3] | -11.1 |  | 11.1 |  | 3.30 |  |
| Maurice | cranmand0054 (W10 N6 712) | 16026 [3] | -9.6 |  | 11.3 |  | 3.30 |  |
| Christian Is | cranmand 0001(TPIF8B3)/M3 | 14160 | -9.8 |  | 12.2 |  | 3.30 |  |
| Christian Is | cranmand 002(68W1, 70WIB20)/M_2_ | 14161 | -8.7 |  | 12.4 |  | 3.20 |  |
| Christian Is | maxillary, no provenience/M_2_ | 14162 | -8.4 | -10.8 | 12.4 | 11.4 | 3.20 | 3.40 |

**S3. Maize δ^15^N Adjustments**

δ^15^N and δ^13^C values were obtained by the University of Georgia Center for Applied Isotope Assays on 13 maize kernels and one cob fragment recovered from ancestral Wendat village sites (Table S4.1). The δ^15^N values ranged from 3.8‒7.5‰ higher than the expected <3‰ (Pfeiffer et al. 2016). Experiments on Eastern Hemisphere grains have shown that charring can affect stable isotope values, with mean offsets of 0.31‰ to 1.0‰ for δ^15^N, while changes in δ^13^C are negligible (Nitsch et al. 2015; Styring et al. 2013) We carried out a series of experiments to determine if charring affects stable isotope values for maize kernels. Mean offsets for δ^15^N were 0.06±0.57‰ at 180^◦^C, 0.51±0.59‰ at 220^◦^C, and 0.96±0.20‰ at 260^◦^C. Mean offsets for δ^13^C were 0.00±0.29‰ at 180^◦^C, 0.02±0.36‰ at 220^◦^C, and 0.56±.38‰ at 260^◦^C. Kernels heated at 180^◦^C did not fully carbonize and those heated at 260^◦^C did not maintain their structural integrity (Fig. S4.1) similar to results obtained by others (Dezendorf 2013; King 1987). It is unlikely that either would have survived in the archaeological record. The kernels heated at 220^◦^C fully carbonized and maintained their structural integrity appearing much like charred kernels recovered from archaeological sites. As a result, the mean δ^15^N offset for these kernels was subtracted from the archaeological kernel mean δ^15^N values, and the standard deviation for the archaeological kernel values was adjusted with the standard deviation of the experimental offset mean through error propagation calculation. This resulted in a range in δ^15^N values for the archaeological kernels of 3.33-6.99‰ and a mean of 4.9±0.9‰.

Table S3.1. Stable isotope values for ancestral Wendat maze.

| UGAMS No. | Site | Century | Material | δ^15^N | δ^15^N adj | δ^13^C |
| --- | --- | --- | --- | --- | --- | --- |
| 32755 | Coulter | Fifteenth | kernel | 5.98 | 5.47 | -9.47 |
| 32756 | Coulter | Fifteenth | kernel | 3.84 | 3.33 | -9.31 |
| 32991 | Baker | Fifteenth | kernel | 6.49 | 5.98 | -9.40 |
| 32992 | Baker | Fifteenth | kernel | 4.41 | 3.90 | -9.31 |
| 32994 | McNair | Sixteenth | kernel | 4.61 | 4.10 | -10.32 |
| 32995 | McNair | Sixteenth | cob | 4.87 | 4.36 | -9.70 |
| 33005 | Damiani | Sixteenth | kernel | 5.22 | 4.71 | -9.64 |
| 33006 | Damiani | Sixteenth | kernel | 4.48 | 3.97 | -8.89 |
| 33008 | Parsons | Sixteenth | kernel | 4.54 | 4.03 | -9.24 |
| 33009 | Parsons | Sixteenth | kernel | 6.54 | 6.03 | -9.81 |
| 37833 | Spang | Sixteenth | kernel | 5.20 | 4.69 | -9.65 |
| 37834 | Spang | Sixteenth | kernel | 7.04 | 6.53 | -8.73 |
| 38397 | Spang | Sixteenth | kernel | 5.94 | 5.43 | -9.44 |
| 38398 | Spang | Sixteenth | kernel | 7.50 | 6.99 | -8.22 |
| Mean | |  |  | 5.48 | 4.90 | -9.37 |
| Standard Deviation | |  |  | 1.11 | 0.90 | 0.51 |

| **Uncharred Maize:**  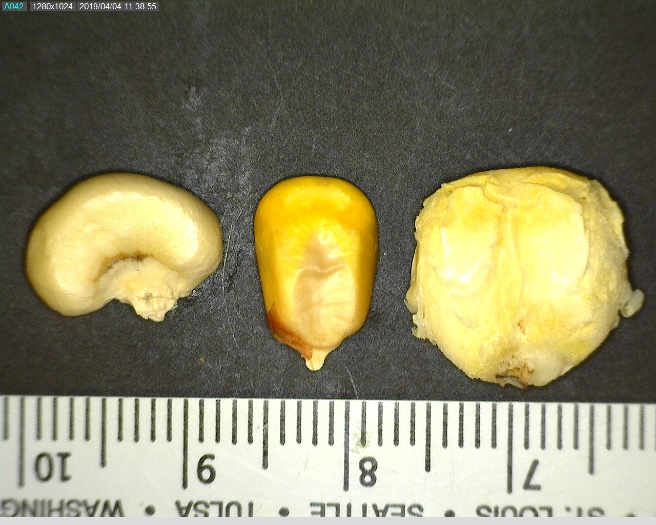 | |
| --- | --- |
| **180°C Charred Maize:**  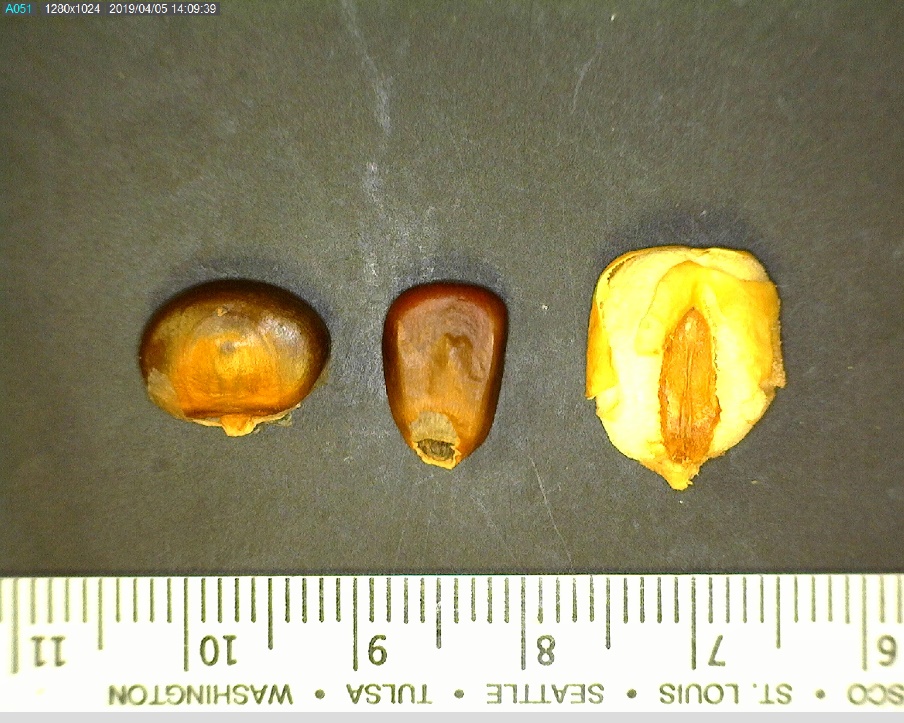 | **220°C Charred Maize**:  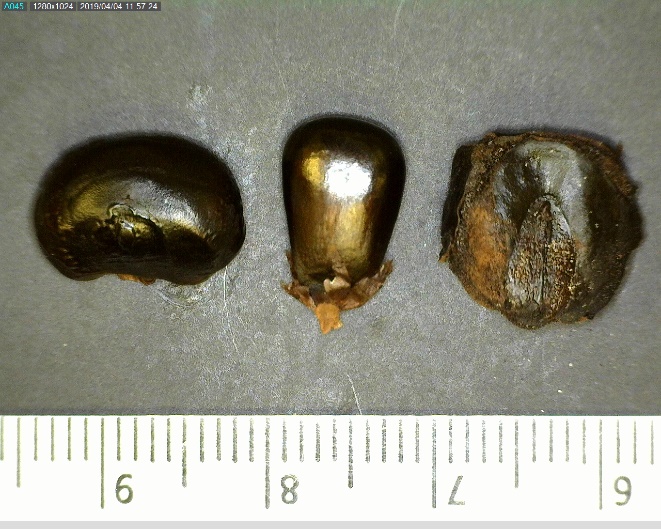 |
| **260°C Charred Maize:**  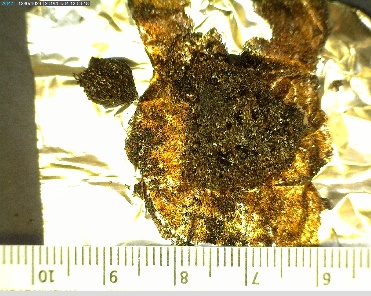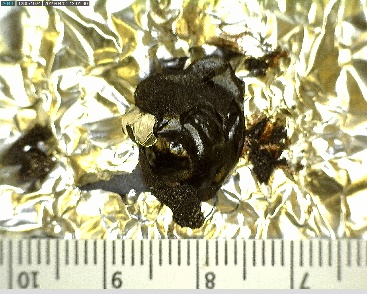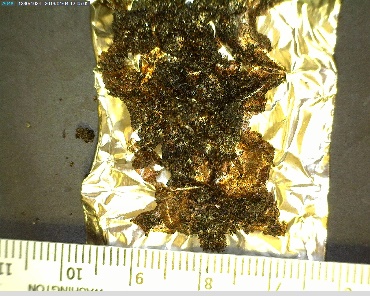 | |

Figure S4.1. Images of whole uncharred and charred maize kernels. In each picture from left to right, Tuscarora white flour maize, Dent maize, and commercial hominy.

**References:**

Booth, L. *A Stable Isotope Analysis of Faunal Remains from Special Deposits on Ontario Iroquoian Tradition Sites* (The University of Western Ontario 2014). <https://ir.lib.uwo.ca/etd/2644>.

Clementz, M.T. & Koch, P.L. Differentiating aquatic mammal habitat and foraging ecology with stable isotopes in tooth enamel. *Oecol.* **129,** 461-472 (2001). <https://doi.org/10.1007/s004420100745>

Dezendorf, C. The effects of food processing on the archaeological visibility of maize: An experimental study of carbonization of lime-treated maize kernels. *Ethnobiol*. *Let*. **4,** 12-20 (2013). <http://dx.doi.org/10.14237/ebl.4.2013.10>

King, F. B. *Prehistoric Maize in Eastern North America: An Evolutionary Evaluation* (University of Illinois at Urbana-Champaign 1987).

Guiry, E. J., et al. Lake Ontario salmon (*Salmo salar*) were not migratory: A long-standing historical debate solved through stable isotope analysis. *Sci. Rep.* **6,** 36249 (2016). <https://doi.org/10.1038/srep36249>.

Hammer, Ø., Harper, D. A. T, Ryan, P. D. PAST: Paleontological Statistics Software Package for education and data analysis. *Palaeontol. Electron.* 2001; 4(1): 9pp. (2001). <http://palaeo-electronica.org/2001_1/past/issue1_01.htm>

Morris, Z. H. *Reconstructing Subsistence Practices of Southwestern Ontario Late Woodland Peoples (A.D. 900-1600) Using Stable Isotopic Analyses of Faunal Material* (The University of Western Ontario 2015). <https://ir.lib.uwo.ca/etd/2921>.

Morris, Z., White, C., Hodgetts, L. & Longstaffe, F. Maize provisioning of Ontario Late Woodland turkeys: isotopic evidence of seasonal, cultural, spatial and temporal variation. *J. Archaeol. Sci. Rep.* **10,** 596-606 (2016). <https://doi.org/10.1016/j.jasrep.2016.06.017>.

Nitsch, E. K., Charles, M., & Bogaard, A. Calculating a statistically robust δ13C and δ15N offset for charred cereal and pulse seeds. *STAR: Sci. Technol. Archaeol. Res.* **1,** 1-8 (2015). <https://doi.org/10.1179/2054892315Y.0000000001>

Pfeiffer, S., Sealy, J. C., Williamson, R. F., Needs-Howarth, S., & Lesage, L. Maize, fish, and deer: investigating dietary staples among ancestral Huron-Wendat villages, as documented from tooth samples. *Amer. Antiq.* **81,** 515-532 (2016). <https://doi.org/10.1017/S0002731600003978>.

Styring, A., et al. The effect of charring and burial on the biochemical composition of cereal grains: investigating the integrity of archaeological plant material. *J. Archaeol. Sci.* **40,** 4767-4779 (2013). <https://doi.org/10.1016/j.jas.2013.03.024>
